# Matrix and graphical representation of the primary headache syndromes in the International Classification of Headache Disorders (ICHD3): a basis for automated diagnosis and analysis of criteria

**DOI:** 10.3389/fneur.2026.1812996

**Published:** 2026-05-11

**Authors:** Pengfei Zhang, Roger Cheng

**Affiliations:** 1 Department of Neurology, Beth Israel Deaconess Medical Center, Boston, MA, United States; 2 Department of Neurology, Harvard Medical School, Boston, MA, United States; 3 Department of Neurology, Rutgers Robert Wood Johnson Medical School, New Brunswick, NJ, United States

**Keywords:** bipartite projection, chronic migraine, migraine, headache classification, Markov clustering algorithm, tension type headache, trigeminal autonomic cephalalgias, ICHD3

## Abstract

**Objective:**

We represent primary headaches within the International Headache Classification (ICHD3) in matrix form and show that this representation allows for automated diagnosis as well as additional insights into headache classification.

**Background:**

ICHD3 has been the gold standard for clinical trials and research in headache medicine. Given its criteria-based language, ICHD3 can be interpreted as a set of logical statements and as a result can be encoded as the biadjacency matrix of a mathematical graph. This paper implements this interpretation of the classification and explores the clinical and theoretical implications of this approach.

**Methods:**

Each diagnosis in the ICHD3 is defined by a list of characteristics. Combinations of characteristics form phenotypes. Multiple phenotypes may fit a given diagnosis. We first translated all characteristics for primary headache diagnoses in the ICHD3 into true/false statements. We then generated a matrix of valid ICHD3 diagnoses as follows:

1. Each row of the matrix represents a phenotype.
2. Each column of the matrix represents a characteristic.
3. If any phenotype contains a characteristic, then that element is encoded as
  1. Otherwise, it is encoded as 0.

From this matrix, we calculated its bipartite projection and Markov cluster. We also row reduced to derive the basis vectors that span the space of all headache phenotypes.

**Results:**

Chronic migraine diagnoses as well as the characteristics “greater than 15 days per month” and “more than 3 months” have the strongest associations based on bipartite projection. Markov clustering yields 64 clusters. These clusters can be organized by ICHD3 diagnoses and demonstrates the level of fragmentation of individual diagnoses within the classification: Migraine is composed of 1 cluster, for example, whereas paroxysmal hemicrania can be broken down into 9 clusters. Finally, row reduction of our matrix yields 63 basis vectors, implying that all headache diagnoses in the ICHD3 can be represented as linear combinations of 63 characteristics. These 63 characteristics correspond to the following: duration, frequency, aura characteristics, size/location, laterality, clearly remembered onset, TAC features, total number of episodes, severity, nausea/vomiting, photophobia, pulsating, alleviation by triptans, and association with awakening, sexual activity, physical activity, temperature, compression or traction, coughing.

**Conclusion:**

Our result demonstrates that ICHD3 is a mathematical entity and that headache diagnoses can exist in a 63-dimensional vector space. This mathematical embodiment of classification allows us to conduct (1) large scale systematic investigations of relationships between headache and phenotypes, (2) generate a graphical representation of characteristics and phenotypes and (3) potentially improve diagnostic accuracy and efficiency.

## Background

The International Classification of Headache Disorders (ICHD3) has been widely used as the standard for headache diagnosis in both clinical and research settings (1). Constructed with criteria-based language, the classification offers precise definitions for each primary and secondary headache disorders. As a result, ICHD3 inherently contains a logical structure (2, 3). This paper attempts to study the logical nature of diagnostic criteria at scale. Specifically, we propose a numerical embodiment of primary headache diagnoses in ICHD3 in the form of a matrix equivalent. Since there is a natural relationship between matrices and networks, we also developed a graphical representation of the classification. We then demonstrate that this matrix/graphical form of the ICHD3 allows us to study how headache phenotypes or diagnoses are correlated due to the classification's inherent structure. We will also show that this matrix formulation allows us to interpret diagnostic criteria, and therefore primary headaches in general, as members within a mathematical vector space. Finally, our matrix formulation of the international criteria allows us to create a method by which to automate ICHD3 diagnosis of primary headaches.

## Methods

We define a headache *characteristic* as a variable that takes on a Boolean value (either True or False). We define a headache *phenotype* as a combination of headache characteristics (More technically: a *phenotype* is a propositional formula with its variables being *characteristics*, involving only conjunction). A *headache disorder*, or simply *disorder* in this paper, is composed of one or more phenotypes.

For example, “photophobia” is a headache *characteristic*. A patient with a combination of *characteristics* including headaches that are “unilateral,” “moderate to severe,” “lasting 4 h to 3 days”, with “photophobia,” “phonophobia,” but “no nausea,” has one of the many headache *phenotypes* which will satisfy the criteria for a *headache disorder* called “migraine without aura.” [We take these definitions from prior modeling of ICHD3 (3, 13)].

### Inclusion/exclusion

In our study, we extracted the criteria for diagnosis of primary headache disorders up to two levels deep in ICHD3. We excluded “Complications of migraine” (1.5) and “Episodic syndrome that may be associated with migraine” (1.6) since these diagnoses depend on a primary diagnosis of migraine. We have retained the criteria for chronic migraine in our study due to its relevance in our field.

### Matrix construction

We constructed a matrix with the following rules:

1. We arranged phenotypes along the row of the matrix. Phenotypes are named by their ICHD3 diagnosis, followed by a numerical designation.
2. We arranged characteristics along the columns of the matrix.
3. If a phenotype on row X contains a specific characteristic on column Y, then the cell (X,Y) is labeled as 1. Otherwise, it is labeled as 0.

Of note, the ubiquitous “not better accounted for by another ICHD-3 diagnosis” in the criteria is not included in our construction to avoid creating a logical impasse. This matrix is included as Supplementary file “data sheet 7.csv” of this paper.

To aid with understanding the generation of the matrix, we will demonstrate how we encode two headache disorders–new daily persistent headache (NDPH) and migraine without aura.

In the ICHD3, NDPH is defined as the following:

A. Persistent headache fulfilling criteria B and C.
B. *Distinct and clearly remembered onset*, with pain becoming *continuous* and *unremitting within 24 h*.
C. Present for >*3 months*.
D. Not better accounted for by another ICHD-3 diagnosis.

We have highlighted the characteristics above with italics and have removed criteria D. Criteria A above simply links criteria A and B with a logical AND. The three phenotypes in these criteria are therefore “distinct and clearly remembered onset,” “continuous,” and “unremitting within 24 h.” ICHD3 uses “continuous” and “constant” as synonym, so the logical equivalent for the above is: “distinct and clearly remembered onset” AND “constant” AND “more than 3 months” AND “unremitting within 24 h.” Since no OR operator is involved, this disorder only contains one phenotype and is represented by the row “ndph1” in our matrix, where a cell is 1 for the above characteristics in the corresponding column and 0 elsewhere.

Migraine without aura is defined as the following by the ICHD3:

A. *At least five attacks* fulfilling criteria B–D.
B. Headache attacks *lasting 4–72 h* (when untreated or unsuccessfully treated).
C. Headache has at least two of the following four characteristics:
  1. *Unilateral location*.
  2. *Pulsating quality*.
  3. *Moderate or severe pain intensity*.
  4. *Aggravation by or causing avoidance of routine physical activity* (e.g., walking or climbing stairs).
D. During headache at least one of the following:
  1. *Nausea and/or vomiting*.
  2. *Photophobia and phonophobia*.
E. Not better accounted for by another ICHD-3 diagnosis.

Again, we take out criteria E and have highlighted the characteristics with italics in our encoding. Since each line above has only 1 characteristic, we will use the shorthand A for “At least 5 attacks,” B for “lasting 4 to 72 h,” C1 for “unilateral location,” etc. Logically, the above can be translated into the following:


$$\begin{array}{l} \begin{array}{c} A\wedge B\wedge [(C1\wedge C2)\vee (C1\wedge C3)\vee (C1\wedge C4)\\ \vee (C2\wedge C3)\vee (C2\wedge C4)\vee (C3\wedge C4)]\wedge (D1\vee D2)\wedge E \end{array} \end{array}$$

One can expand the above into disjunctive normal form:


$$\begin{array}{l} \left(A\wedge B\wedge C1\wedge C2\wedge D1\wedge E\right)\vee \left(A\wedge B\wedge C1\wedge C2\wedge D2\wedge E\right)\vee \\ \left(A\wedge B\wedge C1\wedge C3\wedge D1\wedge E\right)... \end{array}$$

Here, each of the above conjunctive clauses (i.e., AND statements) can be represented as individual rows, i.e., phenotypes, in our matrix. For example, (*A*∧*B*∧*C*1∧*C*2∧*D*1∧*E*), the first conjunctive clause in the disjunctive normal form, represents the phenotype where the following is true: “At least 5 attacks,”, “lasting 4 to 72 h,” “unilateral location,” “pulsatile quality,” “nausea and/or vomiting.” This is therefore row “migraine w/o aura6” in our matrix. All of the above conjunctive statements are enumerated and incorporated as rows in our matrix to represent the totality of the disorder we call migraine without aura [This example is taken, with modification, from our prior paper on prime encoding (3)].

### Our editorial principles

Translation from one language to another is also an act of interpretation; the same can be said for our translation of the ICHD3 classification criteria to Boolean logic. For the sake of transparency and future upkeep of the matrix, we would like to note that we attempt to adhere to the following editorial principles in cases of ambiguity:

First, there are phrases in headache medicine that are considered as parlance to the discipline. These phrases are kept together even if a logical operator is embedded within it. For example, the phrase “distinct and clearly remembered onset” in NDPH is not considered two separate characteristics joined by AND. Instead, we interpret this phrase to describe a particular kind of clinical phenomenon where the patient remembers the onset of a particular headache clearly, as is often the case in the study of NDPH, as opposed to long standing migraine, for example (20).

The ICHD3 can contain “semi-logical” statements that do not correspond to Boolean logic. The most common one is the phrase “and/or” which is by nature logically ambivalent for our purpose—since it is literally read as “AND” OR “OR.” In all cases except for the characteristics of “nausea and/or vomiting” and “no nausea and/or vomiting” of migraine and tension-type headaches—which we considered as headache medicine parlance—we interpret “and/or” as OR unless there is further qualifying condition. The reason is as follows: in primary coughing headaches, for example, notice that if a headache is “brought on by and occurring only with coughing, straining” AND “other Valsalva maneuvers” then it automatically satisfies either “brought on by and occurring only with coughing, straining” OR “other Valsalva maneuvers.” (In other words, if a headache is both brought on by cough AND can be brought on by other Valsalva, then it is simply a concurrent diagnosis of two phenotypes of primary cough headache—one that admits to coughing as a trigger and the other that admits to Valsalva as a trigger, making encoding of a “both” phenotypes redundant).

Often times characteristics are linked with AND in singular occurrences which can in turn be interpreted as simply one unit of characteristics. In these cases, we attempt to respect the language of the ICHD3 and not combine these characteristics. For example, we do not view criteria B of NDPH as 1 characteristic but distinct ones.

We encode intervals of time separately even if they overlap. For example: “15 min to 3 h” overlaps with “2 to 30 min.” We do not encode “2 to 15 min,” “15 min to 30 min,” and “30 min to 3 h” but rather simply leave the original statement as their own independent characteristics.

Finally, negative statements are encoded as either present or absent. For example: “no photophobia” is a distinct characteristic from “photophobia.”

The implication of these editorial choices is further explored below in the limitations section.

### Methods of analysis

Matrix interpretation of ICHD3 allows for numerical analysis of the relationship between phenotypes (the row) and characteristics (the column). We applied two commonly used data science techniques to our matrix: (1) bipartite projection and (2) Markov clustering. We also applied Gaussian elimination to our matrix to obtain the basis vectors in row reduced echelon form (RREF). We will provide a brief overview of each of the above methods for the lay reader; our citations provide more in-depth references for each of the methods:

A bipartite graph is a mathematical graph containing two disjoint groups of vertices where no two vertices in the same group are connected (4). In graphs with this kind of property, one can calculate the graph's “bipartite projection,” a measurement of how individual vertices in one group are associated with each other based on connections to the vertices in the other group (e.g., shared neighbors) (5–7). In our case, headache phenotypes and headache characteristics are two disjoint groups of nodes. An edge exists between a phenotype and a characteristic if that element of the matrix is denoted as “1.” Using bipartite projection, we can determine which phenotypes (i.e., diagnoses) are associated with each other; the strength of these associations (weight) is based on the number of shared connections to corresponding headache characteristics (the other group). The converse can also be done—associating characteristics based on connections to phenotypes, but being a bipartite graph, there can be no connections between two phenotypes, or connection between two characteristics. In this study, we additionally identify the pairings of phenotypes as well as pairings of characteristics with the two highest weightings (6).

Since the ICHD3 matrix is the biadjacency matrix of a mathematical graph, we then apply the Markov clustering algorithm to the matrix to identify groupings within the ICHD3 (8, 9). Conceptually, Markov clustering is used to identify probability distributions that would result from random walks over a given graph (stochastic flow). Markov clustering relies on two parameters: expansion and inflation. To assess optimal parameters, we updated and applied the modularity method in Guy Allard's Markov Clustering Python library for our purpose (9, 10). Briefly, modularity methods refer to the optimization of modularity measures – a measurement of community structure—to pick optimal parameter values in a Markov clustering algorithm (10). We then implemented the Markov clustering algorithm using custom Python code with those optimal parameters.

### The vector space of headache diagnosis

Algebraically, any matrix can be interpreted as a combination of vectors. The technique of Gaussian elimination—transforming matrices into row reduced echelon form (RREF)—is one way of identifying the minimum number of vectors needed to represent the same information presented in the matrix. These vectors are called basis vectors, and their possible combinations, through addition and multiplication, form a *vector space* (14). Our matrix is no different: the ICHD3 (our matrix) can be viewed as a combination of characteristics (vectors). We therefore applied RREF to our matrix to identify the minimum number of characteristics which when used in combination can describe all diagnoses in ICHD3.

### Automated diagnosis

Finally, the matrix representation of ICHD3 can be used for automating diagnosis of headache disorders. One can reformulate a given patient's response (0 for false and 1 for true) to each of the characteristics presented in the matrix as a vector. Multiplication of this vector with our matrix yields a new matrix, such that, if there is a row which is unchanged from the original, then that row is the diagnosis. Mathematically, this is equivalent to calculating the row sum of the original matrix and matching it with that of the resultant vector after matrix multiplication—the row(s) that has the same value for both are the diagnoses. We provide a mathematical proof of this in our Supplementary file data sheet 12.pdf (This proof was first presented at the 18th European Headache Congress under the poster entitled “*Which types of headaches in the migraine and tension-type spectrum cannot be diagnosed by ICHD3?*” and is reproduced here with permission) (17). Of note, this diagnostic technique of matrix representation is equivalent to the prime number encoding which was presented in a prior project (3).

To demonstrate feasibility of this technique of automated diagnosis, we have generated three examples in the Supplementary file. The descriptions of the examples as cases as well as a walkthrough of the automated diagnosis process are in “data sheet 8.pdf”

### Implementation

The generation and extraction of ICHD3 into a matrix form is done manually and through custom code using the programming language Haskell. Once the matrix is generated, we used the Python libraries *pandas, matplotlib*, and *numpy* to solve for the bipartite projection. Markov clustering was accomplished with custom code as well as Allard's *Markov Clustering* Python library (9). Since any adjacent matrix is a mathematical graph, we used custom Python code to translate the matrix into pairings of edges so that *Cytoscape*, version 3.10.4, a graphic/network visualization software package, can be used for graphic visualizations (11). For matrix multiplication in we used SageMath.

## Results

**Figure 1:**
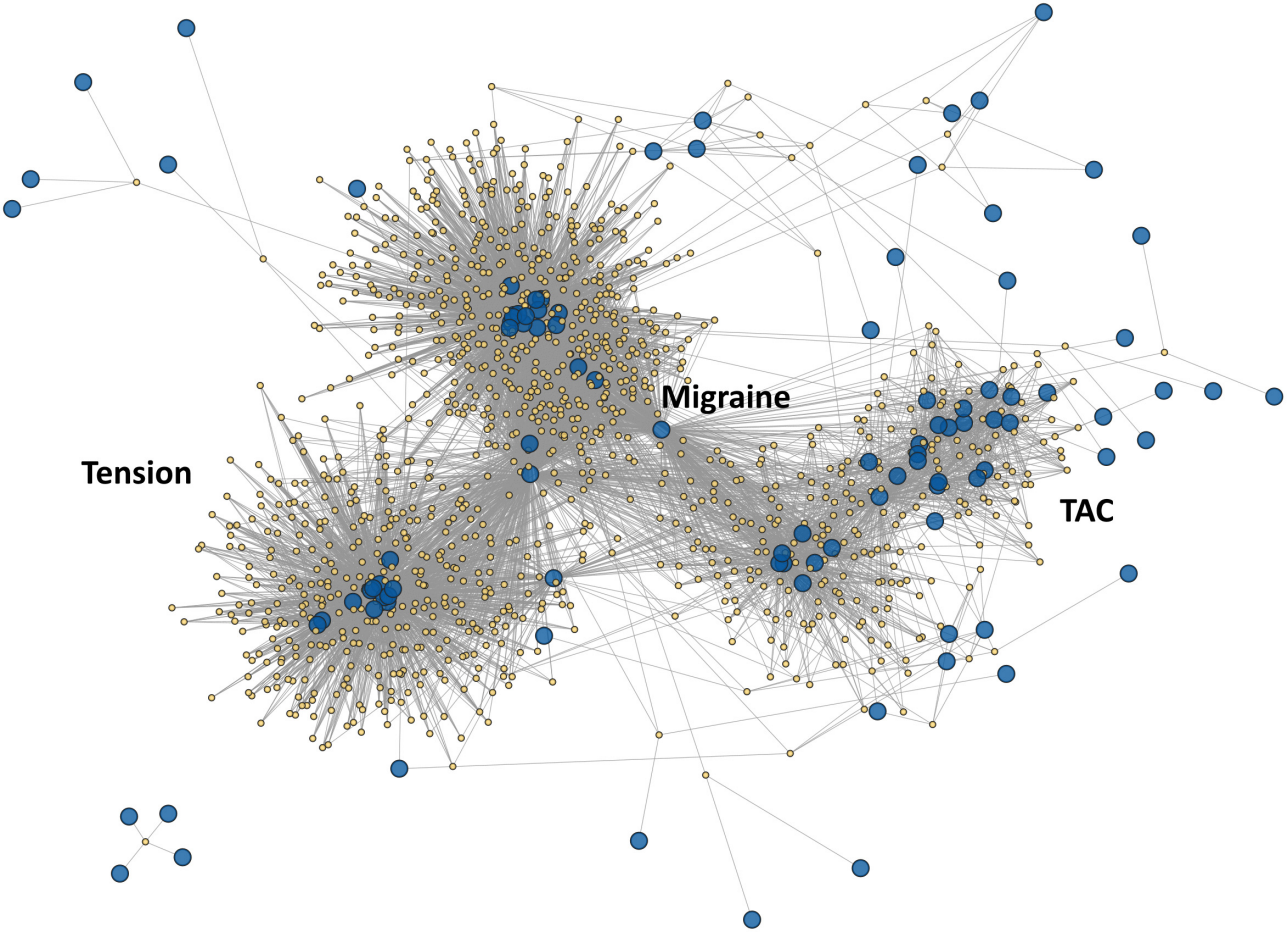
Graphical representation of the ICHD3 primary headache disorders matrix as a network graph. Large blue nodes are headache characteristics (columns), while the smaller yellow nodes are headache phenotypes (rows). Gray lines represent edges connecting each phenotype to its characteristics. Broadly, this “galaxy” map can be described as having a main central axis comprising the migraine disorders, 2 auxiliary high density areas comprising the tension type headaches and trigeminal autonomic cephalalgias, and finally the pathognomonic disorders scattered in the periphery.

**Figure 2:**
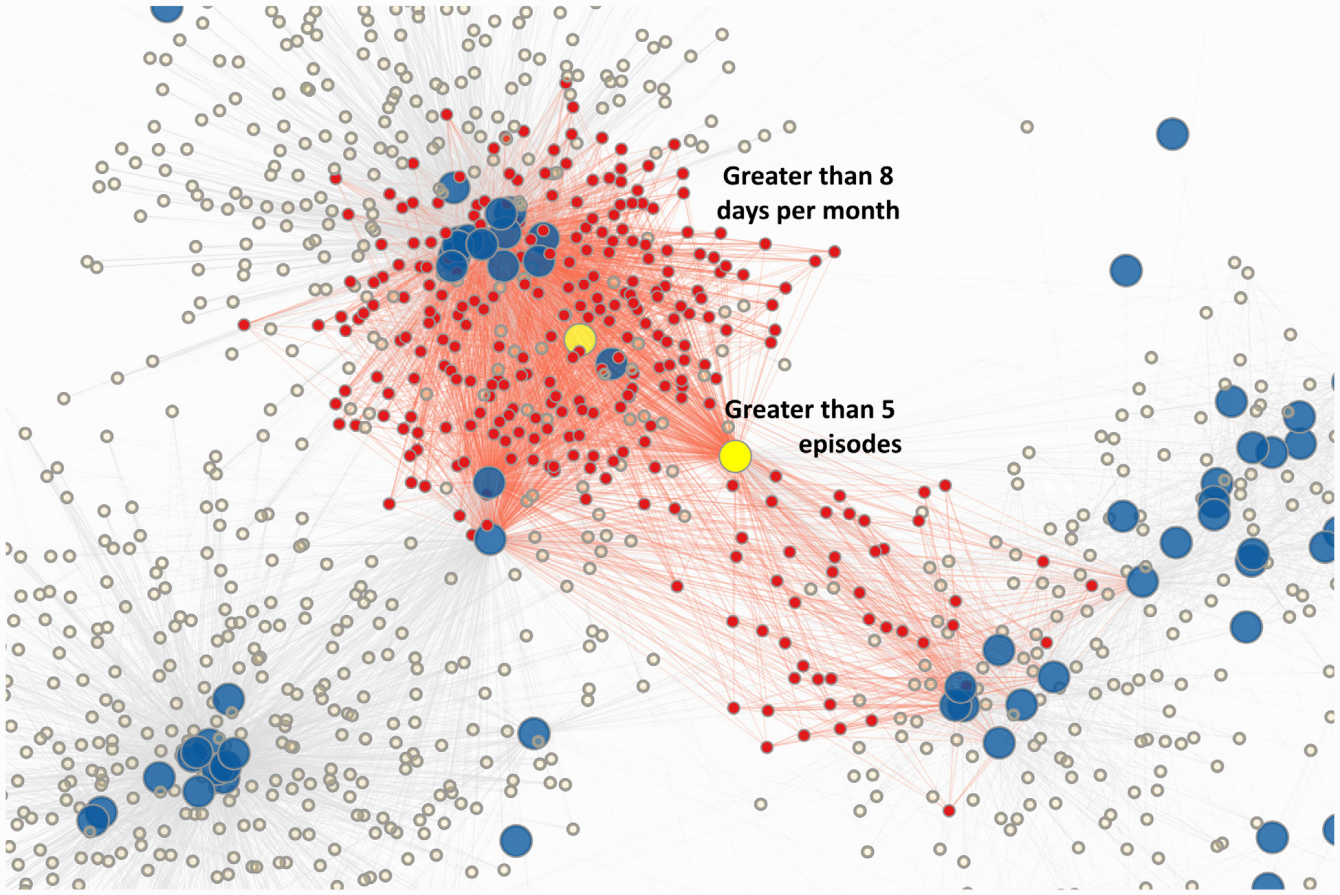
Chronic migraine clusters. In this zoomed in view, phenotypes for chronic migraine and the corresponding edges are highlighted in red. Two main large clusters are formed, with interconnections to the characteristics “Greater than 8 days per month” and “greater than 5 episodes” (yellow).

**Figure 3:**
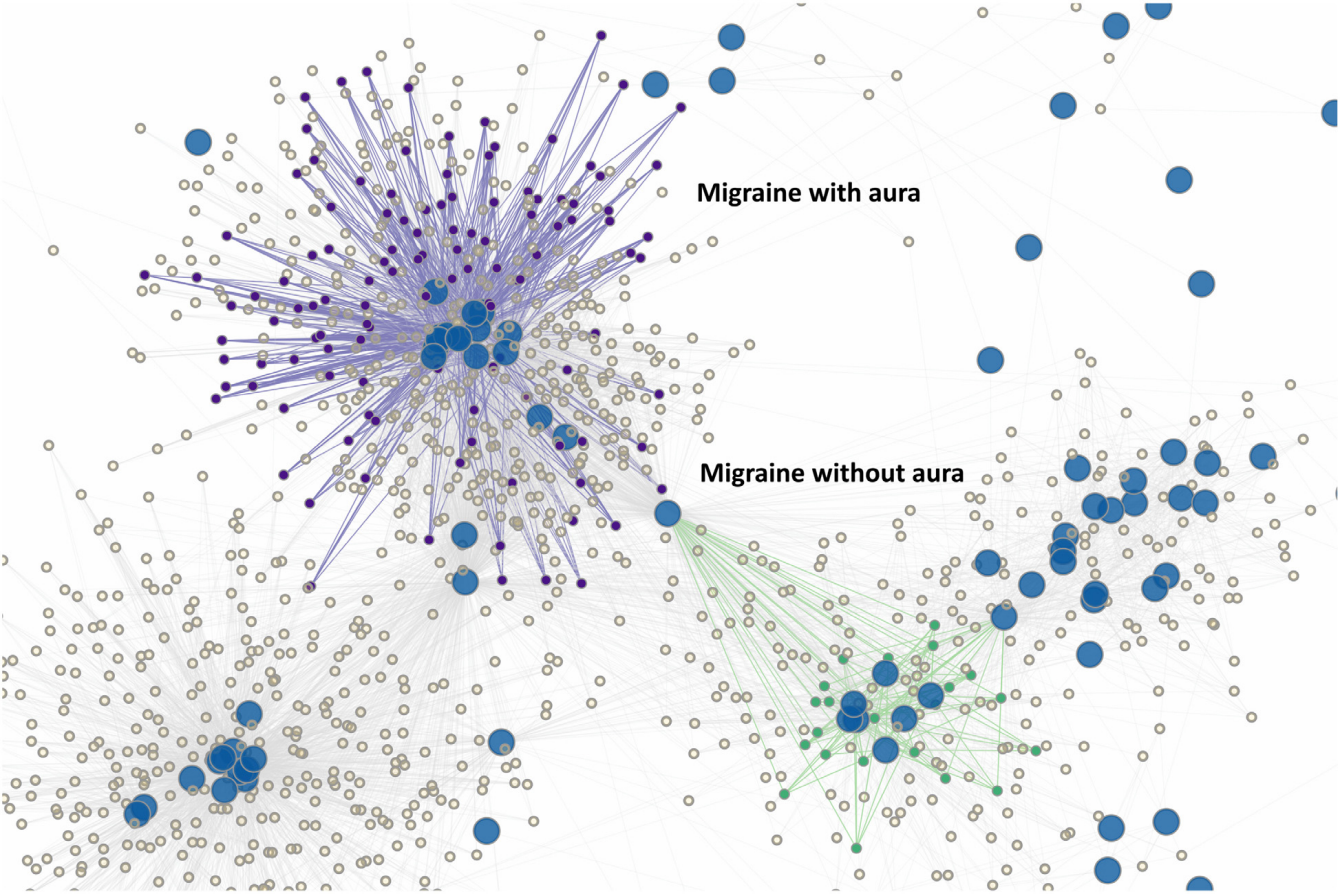
Episodic migraine clusters. Episodic migraine with aura (purple) and without aura (green) as expected overlays the clusters formed by chronic migraine (Figure 2) due to similar symptomatology, but interestingly are completely disjointed due to the diagnosis of migraine with aura being based entirely on aura characteristics rather than quality of headache. Note again that the node “greater than 5 episodes” again is a key characteristic, but only for migraine without aura (as migraine with aura only requires 2 episodes).

**Figure 4:**
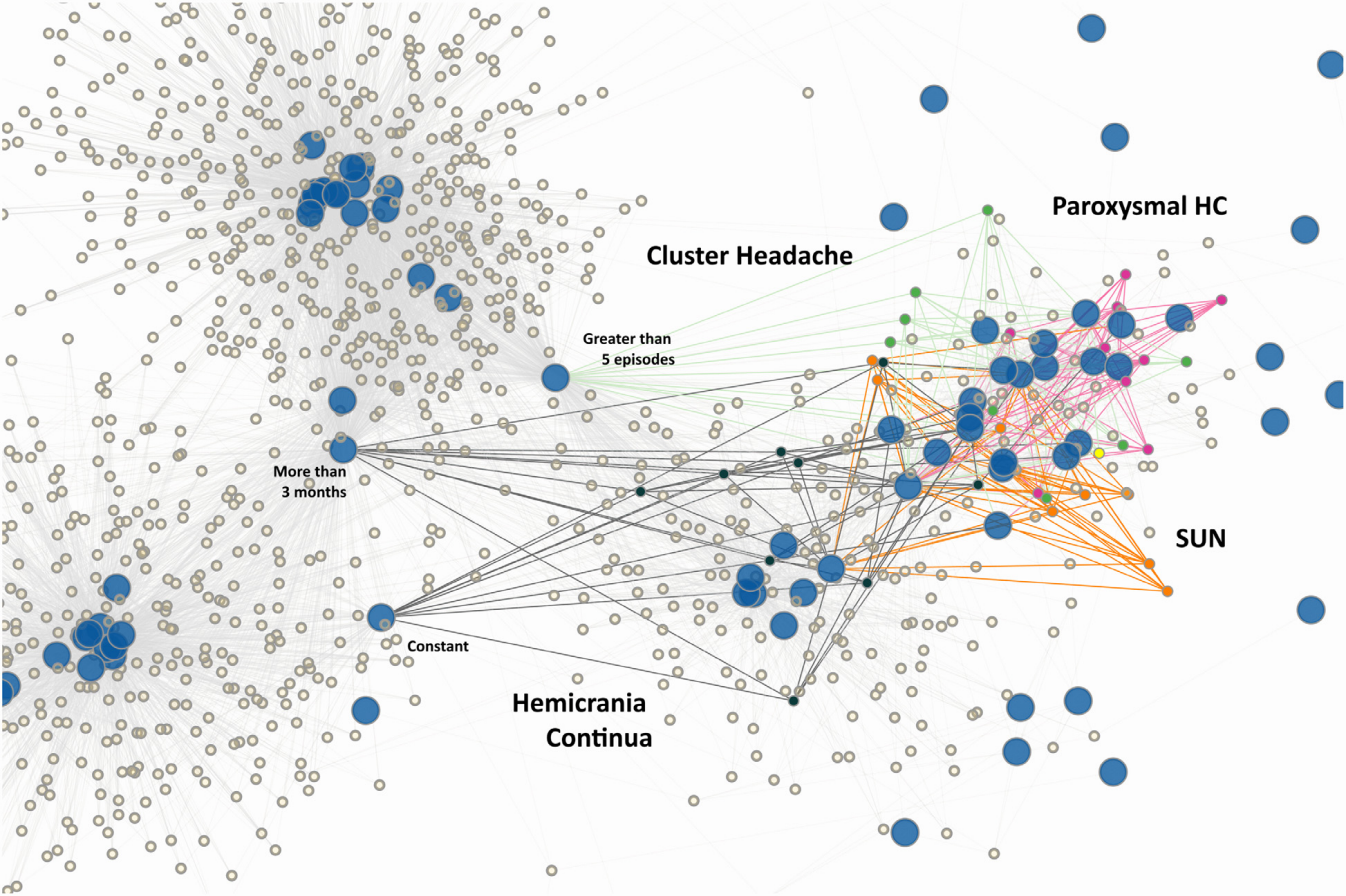
Trigeminal autonomic cephalalgias (TAC). Here, we see that paroxysmal hemicrania (magenta), sudden unilateral neuralgiform headache (orange), hemicrania continua (black), and cluster headache (green) all occupy a loose region, without a distinct group for any given TAC. Note again how timing and frequency characteristics again are strong defining characteristics for cluster headache and hemicrania continua independent of headache quality.

**Figure 5:**
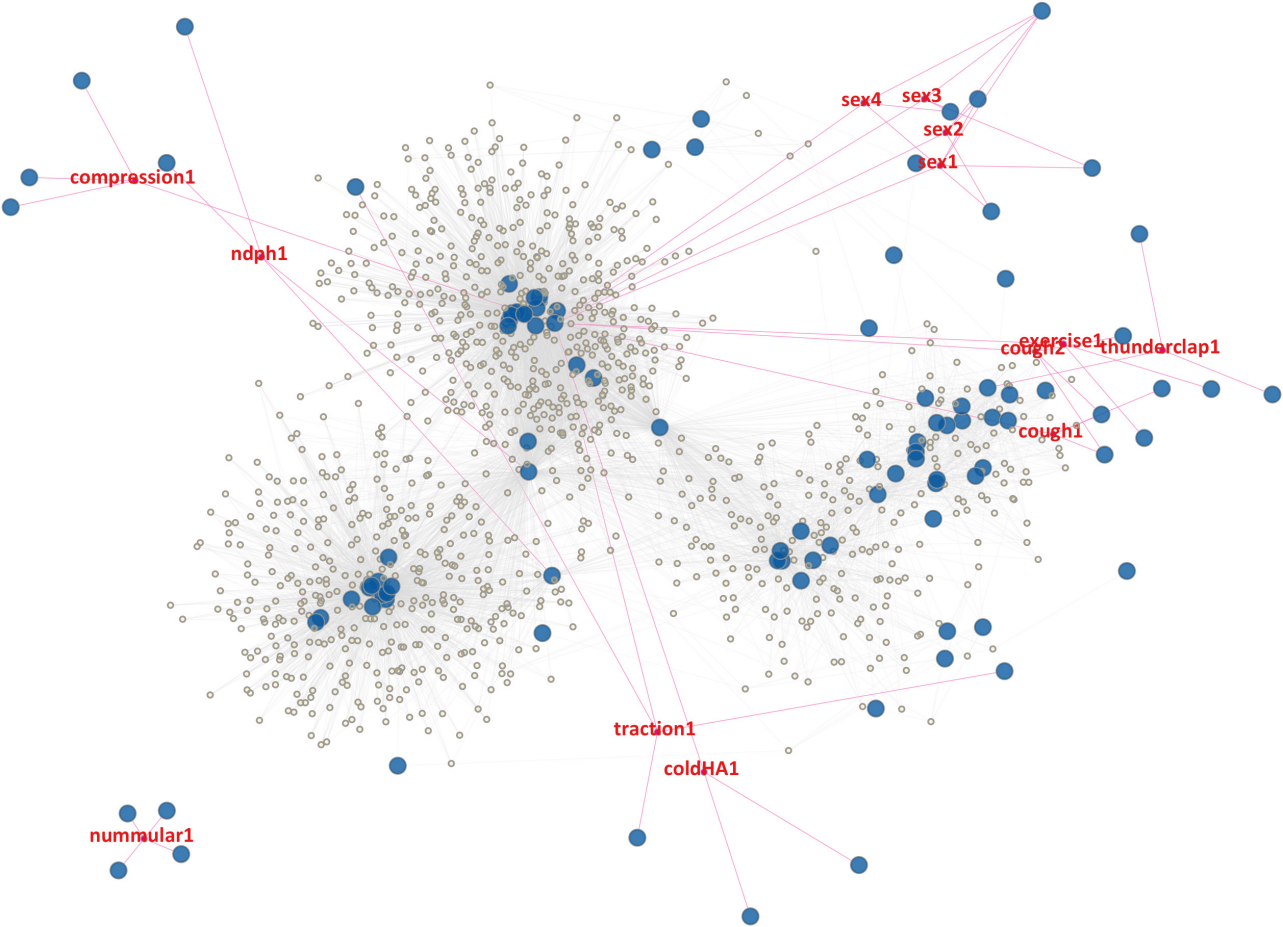
Pathognomonic headache disorders. Located in the periphery are headache disorders which have a small number of extremely specific defining features. Note that nummular headache is disconnected entirely from the remainder of the network due to sharing no characteristics with any other primary headache disorder.

Figure 1 represents a graphical representation of the ICHD3 primary headache disorders in this manuscript. To offer better visualization of the individual components, we offer a “zoomed in” figure of individual major clusters in Figures 2–5.

### Results from bipartite projection

As discussed above, bipartite projections can be done for either phenotypes or characteristics. The highest weight for phenotypes is 11; these phenotypes all consist of pairings of chronic migraine: (“cm27”, “cm24”), (“cm25”, “cm24”), (“cm26”, “cm24”), (“cm29”, “cm24”), (“cm29”, “cm24”). The second highest weight is 10; there are 1,006 pairings, all between various phenotypes of chronic migraine as well.

The highest weight for characteristics is 416; they consist of the pairings between “greater than 15 days per month” and “more than 3 months.” The second highest weight for characteristics is 366 and is between “fully reversible” and “greater than 2 episodes.” We have also obtained the third largest weight for characteristics−284—*post hoc*; the weight belongs to the following pairings: (“greater than 15 days per month,” “greater than 5 episodes”), (“greater than 15 days per month,” “greater than 8 days per month”), (“greater than 5 episodes,” “greater than 8 days per month”), (“greater than 5 episodes,” “more than 3 months”), and (“greater than 8 days per month,” “more than 3 months”).

### Results from Markov clustering

Using modularity measurement, the inflation parameter for Markov clustering is optimal at 3.3. Given this inflation parameter, expansion is then determined—through modularity measurement again—as optimal at 3. (See Supplementary Files under “data sheet 9.pdf” and “data sheet 10.pdf”) Application of these parameters yields 64 clusters (See Supplementary Files. “data sheet 13.pdf”). We manually reviewed each of these clusters and categorized them into those dominated by specific ICHD3 diagnoses. They are as follows:

1. Trigeminal autonomic cephalalgias (TAC).
  a. Clusters 8 to 16 (paroxysmal hemicrania only).
  b. Cluster 17 (SUN, Probable HC, “greater than 1 per day,” “1 to 600 s”).
  c. Cluster 7 (cluster headache).
  d. Cluster 3 (cluster headache, probable cluster headaches, thunderclap headaches, “maximum within 1 min,” “greater than 5 min”).
  e. Cluster 22 (Probable Cluster Headache, Probable Hemicrania Continua, “greater 5 per day,” “miosis”).
  f. Cluster 23 (Probable Hemicrania Continua, Hemicrania Continua, Probable Cluster Headache, “greater than 20 episodes,” “nasal congestion”).
  g. Cluster 20 (Probable Hemicrania Continua, Hemicrania Continua, Probable Cluster Headache, “forehead and facial sweating,” “indomethacin responsive”).
  h. Cluster 21 (Probable Hemicrania Continua13, Probable Cluster Headache4, hc4, “orbital or supraorbital or temporal pain,” “lacrimation”).
  i. Cluster 25 (Probable Cluster Headache, Probable Hemicrania Continua, severe, every other day to 8 per day, 15 to 180 min, restless).
  j. Cluster 19 (Probable Cluster Headache, Probable Hemicrania Continua, hc2, unilateral, and eyelid edema)
  k. Cluster 18 (Probable Cluster Headache1, Probable Hemicrania Continua15, hc1, 2 to 30 min, conjunctival injection)
  l. Cluster 26 (Probable Cluster Headache, hc9, Probable Hemicrania Continua25, rhinorrhea)
  m. Cluster 24 (Probable Hemicrania Continua19, Probable Cluster Headache7, hc7, ptosis)
2. Tension type headaches (TTH)
  a. Cluster 1 (NDPH, frequent TTH, chronic TTH, probable chronic TTH, probable frequent TTH, “constant,” “not aggravated by activity,” “mild to moderate pain,” “no photophobia,” “clearly remembered onset,” “no phonophobia,” “no nausea/vomiting,” “30 min to 7 days in duration,” “1 to 14 days per month,” “more than 3 months,” “non-pulsating,” “unremitting within 24 h,” “hours to days,” “bilateral location”).
  b. Cluster 39 to 60 (infrequent tension type headaches only).
  c. Cluster 61 (probable infrequent tension type headaches, “less than 12 days per year,” “more than 10 episodes”).
  f. Cluster 34 (Probable Chronic Tension Type Headache, stabbing, hypnic, no ptosis).
3. Migraine (with and without aura):
  a. Cluster 0 (chronic migraine, migraine with aura, migraine without aura, probable migraine with aura, probable migraine without aura, “relieve by triptans or ergots,” “moderate to severe,” “at least one aura symptom is unilateral,” “visual aura,” “greater than 15 days per month,” “fully reversible,” “aggravated by physical activities,” “photophobia,” “motor aura,” “phonophobia,” “aura is accompanied, or followed within 60 min by headaches,” “two or more aura symptoms occur in succession,” “pulsating,” “sensory aura,” “at least one aura symptom is positive,” “greater than 2 episodes,” “speech and/or language aura,” “nausea/vomiting,” “4 to 72 h,” “each individual aura symptom lasts 5 to 60 min,” “greater than 5 episodes,” “at least one aura symptom spreads gradually over 5 min,” “brainstem aura,” “greater than 8 days per month,” “retinal aura”).
4. Primary sex headache:
  a. Cluster 27 (probable migraine with aura, sex, “abrupt explosive intensity just before or with orgasm,” “1 min to 72 h with severe,” “up to 72 h with mild,” “increasing in intensity with increasing sexual excitement,” “brought on by sex”).
5. Primary stabbing and hypnic headache:
  a. Cluster 28 (primary stabbing headaches, hypnic headaches, “15 min up to four h after waking,” “more than 10 days per month,” “developing only during sleep and causing wakening,” “no conjunctival injection”).
  b. Cluster 29 (primary stabbing headaches, hypnic headaches, “no eyelid edema”).
  c. Cluster 35 (hypnic, primary stabbing, “no rhinorrhea”).
  d. Cluster 31 (stabbing4, Probable Chronic Tension Type Headache23, hypnic4, “no lacrimation”).
  e. Cluster 30 (hypnic3, stabbing3, Probable Chronic Tension Type Headache20, no forehead and facial sweating).
  f. Cluster 32 (stabbing5, hypnic5, single or series of stabs, no miosis, irregular frequency, up to few seconds).
  g. Cluster 33 (hypnic6, stabbing6, “no nasal congestion”).
6. Cough and Valsalva headache:
  a. Cluster 37(cough1, “sudden,” “between 1 s to 2 h,” “provoke by cough”).
  b. Cluster 38(cough headache, “provoke by Valsalva”).
7. Exercise headaches:
  a. Cluster 2 (exercise headaches, brought on by exercise, less than 48 h).
8. Traction and compression headaches:
  a. Cluster 5 (compression headaches, “brought on within 1 h of compression,” “maximal at site of compression,” “resolve within 1 h after removal of compression”).
  b. Cluster 6 (traction headaches, “brought on within 1 h of traction,” “maximal at site of traction,” “resolve within 1 hour after removal of traction.”
9. Cold induced headaches:
  a. Cluster 4 (cold induced HA, “brought on by cold stimuli,” “resolve within 30 min after removal of cold”).
10. Nummular headache:
  a. Cluster 36 (nummular1, fixed in size and shape, sharply contoured, 1–6 cm in diameter, round or elliptical).
11. Phenotypes only:
  a. Cluster 62 (more than 1 episode per day).
  b. Cluster 63 (no orbital or supraorbital or temporal pain).
  c. Cluster 64 (no restlessness).

Although the above is considered the optimal choice based on modularity, we have also explored Markov clustering at four other sets of parameters: at inflation 1.5 and expansion of 2, inflation 1.5 and expansion of 9, inflation of 3.3 and expansion of 2, inflation of 3.3 and expansion of 9 (The results are presented as Supplemental Files as “data sheet 11.pdf.”). We selected inflations of 1.5 and 3.3 since these represent the minimum and maximum based on modularity; similarly, we selected expansions of 2 and 9 since these represents the lower and upper bound of our testing.

In both cases when expansion is high (i.e., expansion = 9), we obtained dramatically smaller numbers of clusters (3 clusters when inflation is 1.5, and 5 clusters when inflation is 3.3) as compared to when expansion is lower (i.e., expansion = 2): 56 when inflation is 1.5 and 210 clusters when inflation is 3.3. We hypothesize that this is due to the overshooting of the expansion factor beyond the size of the graph, pushing clustering groups to the periphery. In either case when expansions are high, most headache disorders cluster in a single group (cluster group 0 in both settings). Noteworthy is that even with high expansion, nummular headaches and its characteristics forms its own group regardless of inflation–cluster group 3 when inflation is 3.3, cluster group 1 when inflation is 1.5. This is almost true when expansion is low, the characteristics of nummular headache also gathers in cluster 19 and 141 for inflation of 1.5 and 3.3, respectively. Strangely, nummular headache as a diagnosis is in its own cluster–group 11 and 67 for expansion of 1.5 and 3.3, respectively. We hypothesize that this phenomenon occurs because of the pathognomonic nature of nummular headache–its two characteristics are so distinct so as to not be shared by any other disorder and furthermore they are connected by an AND logical operator. As such, it is difficult to separate these characteristics from each other regardless of how random walks are done. Indeed, one can even make the case of putting all of these characteristics as a single characteristic (See limitation section below on translation/interpretation).

Tracking nummular headache is therefore a good way to judge the effect of parameter variations: First, neither expansion nor inflation variation can break the network formed by a logical AND. Second, at low expansion, diagnoses and their characteristics start to come apart and, at extreme cases, cluster groups become too scattered to allow for any meaningful insights to be derived. Third, at high expansion, diagnoses and characteristics merge so much that everything looks the same, subsuming everything under the same group so as to obliterate possibilities for differentiation.

### Row reduction

The pivot columns/basis vectors for all diagnoses (excluding probable diagnoses) are the following 63 characteristics: “1 min to 72 h with severe pain,” “1 to 14 days per month,” “1 to 600 s,” “15 min to up to 4 h after waking,” “15 to 180 min,” “1 to 6 cm in diameter,” “2 to 30 min,” “30 min to 7 days in duration,” “4 to 72 h,” “abrupt explosive intensity just before or with orgasm,” “aggravated by physical activity,” “at least one aura symptom is positive,” “at least one symptom is unilateral,” “at least one symptom spreads gradually,” “between 1 s to 2 h,” “bilateral location,” “brainstem aura,” “brought on by cold stimuli,” “brought on by exercise,” “brought on by sex,” “brought on within 1 h of compression,” “brought on within 1 h of traction,” “clearly remembered onset,” “conjunctival injection,” “constant,” “aura lasting 5 to 60 min,” “eyelid edema,” “forehead and facial sweating,” “fully reversible,” “greater than 15 days per month,” “greater than 5 episodes,” “greater than 5 min,” “greater than 8 days per month,” “hours to days,” “irregular frequency,” “lacrimation,” “mild to moderate,” “miosis,” “moderate to severe,” “motor aura,” “nasal congestion,” “nausea/vomiting,” “no conjunctival injection,” “no eyelid edema,” “no forehead and facial sweating,” “no lacrimation,” “no miosis,” “no nasal congestion,” “no phonophobia,” “no ptosis,” “no restlessness,” “non-pulsating,” “not aggravated by physical activity,” “provoked by coughing,” “ptosis,” “pulsating,” “relieved by triptan,” “restlessness,” “retinal aura,” “sensory aura,” “speech and/or language aura,” “aura is accompanied or followed within 60 min by headache,” and “unilateral headache.”

These characteristics may correspond to the following questions commonly used during headache history taking:

1. Duration.
2. Frequency.
3. Association with awakening.
4. Association with sexual activity.
5. Association with physical activity.
6. Association with temperature.
7. Association with compression or traction.
8. Association with cough.
9. Aura characterization.
10. Size/location.
11. Laterality.
12. Clearly remembered onset.
13. Existence of trigeminal autonomic features.
14. Total number of episodes.
15. Severity.
16. Nausea/vomiting.
17. Photophobia.
18. Pulsating.
19. Alleviation by triptan.

The pivot columns/basis vectors for all diagnoses (including probable diagnoses) are the following: “1 min to 72 h,” “1 to 14 days per month,” “1 to 600 s,” “15 min up to 4 h,” “15 to 180 min,” “1–6 cm in diameter,” “2 to 30 min,” “30 min to 7 days in duration,” “4 to 72 h,” “abrupt explosive intensity just before or with orgasm,” “aggravated by physical activity,” “at least one aura symptom is positive,” “at least one aura symptom is unilateral,” “at least one aura symptom spreads gradually over 5 min,” “between 1 s to 2 h,” “bilateral location,” “brainstem aura,” “brought on by cold stimuli,” “brought on by exercise,” “brought on by sex,” “brought on within 1 h of compression,” “brought on within 1 h of traction,” “clearly remembered onset,” “conjunctival injection,” “constant,” “each individual aura symptom lasts 5–60 min,” “every other day to 8 per day,” “eyelid edema,” “forehead and facial sweating,” “fully reversible,” “greater than 1 per day,” “greater than 15 days per month,” “greater than 2 episodes,” “greater than 20 episodes,” “greater than 5 episodes,” “greater than 5 min,” “greater than 5 per day,” “greater than 8 days per month,” “hours to days,” “indomethacin responsive,” “irregular frequency,” “lacrimation,” “less than 12 days per year,” “mild to moderate pain,” “miosis,” “moderate to severe,” “more than 3 months,” “motor aura,” “nasal congestion,” “nausea/vomiting,” “no conjunctival injection,” “no eyelid edema,” “no forehead and facial sweating,” “no lacrimation,” “no miosis,” “no nasal congestion,” “no nausea/vomiting,” “no phonophobia,” “no ptosis,” “no restless,” “nonpulsating,” “not aggravated by activity,” “phonophobia,” “provoked by cough,” “ptosis,” “pulsating,” “relieve by triptan or ergot,” “restless,” “retinal aura,” “rhinorrhea,” “sensory aura,” “speech and/or language aura,” “the aura is accompanied or followed within 60 min by headache,” “two or more aura symptoms occur in succession,” and “unilateral.” This includes all but 28 columns in the matrix.

## Discussion

In this paper, we demonstrate that the international classification can be translated into matrix form, a mathematical entity that allows for large-scale numerical manipulations and investigation. This numerical version of the classification guideline enables us to study the ICHD3 in the following ways: (1) Identification of correlations between phenotypes, characteristics, and headache disorders, (2) Identification of a headache vector space as defined by characteristics, (3) The generation of automated diagnosis at scale through the use of linear algebra, and (4) Production of a graphical version of the criteria.

We should note that this method is not the only way to produce a mathematical version of ICHD3. Previous work by Costabile et al. (2) shows that ICHD3 can be interpreted logically. Our own works showed that it is possible to map ICHD3 into a number system through a version of Godel numbering (3, 13). Furthermore, this method is also not the only way to describe ICHD3 graphically: the hierarchical nature of ICHD3 as well as its comment section allows for graphical representation of both primary and secondary headaches, which then in turn can be represented as set/subset relationships between differential diagnoses (11, 15).

The study of the structure of headache classification, although theoretical, is still immediately relevant to clinicians and researchers. ICHD3 guides diagnosis and research; any inherent structural bias directly influences patient care. Study of the classification is the study of our contemporary perspective of headache disorders. Our goal in this study is not only to put forth a method of studying classification as well as a method of automated diagnosis, but also to point out potential diagnostic biases–both desirable and undesirable—in primary headache diagnosis.

First, the overabundance of chronic migraine in the bipartite projection highlights the structural importance of the various phenotypes of chronic migraine in the current classification. This also implies that chronic migraine is formed by two tightly clustered and massive groups, as evident in Figure 2. These two groups are held together by two characteristics–“greater than 5 episodes” and “greater than 8 days per month.” This likely explains why various characteristics of chronic migraine also cluster together in the bipartite projection of characteristics: cf “greater than 15 days per month” and “more than 3 months,” “greater than 15 days per month” and “greater than 8 days per month,” “greater than 15 days per month” and “greater than 5 episodes,” “greater than 5 episodes” and “greater than 8 days per month,” “greater than 5 episodes” and “more than 3 months,” as well as “greater than 8 days per month” and “more than 3 months.”

Even though photophobia, phonophobia, and nausea/vomiting are also major characteristics that form the chronic migraine/migraine cluster, it is duration and frequency clusters that exert a stronger relationship. This leads to the question of whether these two characteristics should hold such a powerful role in defining chronic migraine. Although at first glance it may appear surprising why “more than 8 days per month” is an equally relevant characteristic as “more than 15 days per month,” a study of the chronic migraine criteria in the ICHD3 makes this clear–whereas “more than 15 days per month” allows for unification of migraine diagnoses under chronic migraine, “more than 8 days per month” actually allows headaches with tension type phenotype to also be grouped under the classification of “migraine.” In other words, the way the “8 days” rule in the ICHD3—“*On 8 days/month for* >*3 months, fulfilling any of the following 2:1. Criteria C and D for 1.1 Migraine without aura 2. Criteria B and C for 1.2 Migraine with aura 3. Believed by the patient to be migraine at onset and relieved by a triptan or ergot derivative*”–allows the rest of the headache days to be counted toward migraine even if they are not “migrainous” may be a significant factor in the diagnosis of (or at least our perception of) how we should diagnose migraine.

Although characteristics of migraine with aura are unsurprisingly strongly associated–for example “fully reversible” and “greater than 2 episodes” is present as a highly correlated pairing–our study also highlights that the cardinal features of aura are reversibility and reproducibility as opposed to the specific forms of aura present. We believe it is an open question of whether this is desirable.

Markov clustering can be thought of as random walks on Figure 1. Firstly, we should make explicit our assumption of classifying Markov clusters with respect to primary headache diagnoses/phenotypes rather than the characteristics. This is an editorial decision on our part, and it may be equally valid to attempt to classify clusters with characteristics instead. However, we deem our approach more appropriate given that ICHD3 is intended to describe coherent clinically observable entities–i.e., migraines–rather than inspecting how each characteristic can be described by its disease process. So therefore, we preference grouping of phenotypes over grouping with respect to characteristics.

An obvious question is, “Why are some diagnoses composed of a few big clusters, but others a variety of small clusters?” For example, paroxysmal hemicrania (PH) is broken up into 9 small clusters (clusters 8 to 16) whereas migraine can be described as simply 1 big cluster (cluster 0). This fragmentation also occurs for infrequent tension type headache, which occupied 22 cluster groups (clusters 39 to 60). An explanation can be gained by looking into the mathematical graph generated by the adjacency matrix (Figure 1). Whereas the characteristics of “more than 3 months,” “nausea/vomiting,” “greater than 5 episodes,” and other migrainous characteristics appears to have connected 2 major clusters of migraine together like spokes to a wheel, paroxysmal hemicrania of different phenotypes do not appear to share a common characteristic (Figure 4). Rather, the shared characteristics of TACs are a set of characteristics–rhinorrhea, miosis, etc. Since any one of those characteristics is sufficient to identify a phenotype as a TAC, the graphical properties will be more divergent compared to a phenotype where one since mandatory characteristics–such as “more than 3 months”–is required for diagnosis (Indeed, given the result from the bipartite projection–which highlights to close correlation between various phenotypes of chronic migraine–there is no surprise that migraine subtypes are so closely intertwined—as opposed to tension type headache—for example).

In other words, the logical AND statement in the criteria is more unifying than OR statements. In some sense, this should not be surprising: after all, there must be some fundamental pathophysiological differences between a PH patient with rhinorrhea as compared to one with miosis, even if they are both classifiable under the same umbrella condition.

Paroxysmal hemicrania appears to exist in its own distinct clusters (clusters 8 to 16). The same can be said of short-acting unilateral neuralgiform headache (SUN), which only occupies cluster 17. Aside from that, cluster headaches, hemicrania continua (HC), as well as their associated ICHD3 diagnoses (probable cluster headache and probable hemicrania continua), occupy multiple clusters concurrently [3, 20, 21, 22, 23, 25]. The differences among these clusters appear to be only secondary to their associated autonomic features as well as indomethacin responsiveness. There is also one instance where cluster headache shares a common cluster with thunderclap headache. The distribution/clustering of various TAC here is not surprising but does highlight the potential ambiguity in separating between cluster and HC. Furthermore, the finding that there are multiple cluster groups as opposed to 1 major cluster group in migraine suggests that TAC as a class is more “fractured” in its organization–at least in our perception of it–as compared to migraine (Figures 3, 4). For example, migraine with aura presents itself with multiple different kinds of subtypes also – that is, aura phenomenon—and yet remain as 1 group which is unified by, as previously noted, “reversibility” and “producibility” (i.e., more than 2 episodes). This kind of unification is not as strong in the TACs which are not only separated by the kind of autonomic features–miosis, rhinorrhea, etc.–but also by their durations.

Definitive diagnoses of frequent and chronic TTH reside in cluster 1 along with new daily persistent headache (NDPH). This is a peculiar fact but we hypothesize that this is due to that chronic TTH and NDPH share the characteristics of being able to be “unremitting” and “> 3 months.” “Unremitting” is not part of the migraine definition, indeed the “4 h to 72 h” definition eliminates this possibility and therefore eliminates the possibility of a NDPH connection. This reflects an important peculiarity of the current classification system, as NDPH has been classically separated into “migrainous” and “tension type” phenotypes (16). Furthermore, we believe it is a clinically self-evident fact that there are migraine sufferers who have unremitting headaches. Therefore, we believe that allowing migraine to be “unremitting” appears to be appropriate.

Other so-called miscellaneous primary headaches can be organized into their own groups–cough/Valsalva headaches, exercise headaches, traction/compression headaches, cold-induced headaches, and nummular headaches. This is to be expected since these phenomena have pathognomonic “signs” (Figure 5). Indeed, in a study of differential diagnosis classification, these headaches also stand out as unique (12).

Finally, primary stabbing headaches and hypnic headaches appear to share a continuum due to their clustering in multiple groups [28, 29, 30, 31, 32, 33, 35]. This can likely be explained by their criteria sharing the requirement of a lack of TAC features. This is evidenced by the fact that TAC features are broken down into the lack of miosis, rhinorrhea, etc., and these are characteristics present in the various clusters in the stabbing/hypnic headache clusters.

### Vector space

Row reduction of our matrix yields 63 characteristics as basis vector once all probable headache diagnoses are removed. This implies that those 63 characteristics, in various combinations, are sufficient to describe all definitive diagnoses of primary headaches. There are two implications of this result:

Firstly, on a clinical and practical level: since those 63 characteristics can be described by inquiring about 19 characteristics of headaches, this means that one should be able to diagnose/differentiate primary headaches from each other by using 19 questions. Of note, this is not the minimum number of questions needed to diagnose/differentiate headaches from each other. Our previous paper shows that this number can be further trimmed down (13). However, this result improves upon our prior result since the previous result relies heavily on duration of headaches as a means of differentiation. Duration, and frequency, as discussed previously, may not accurately describe clinical encounters; the current result, relying less so on duration, may offer a more accurate tool. Of course, our study does not include secondary headaches and so as a result, we do not recommend using the 19 questions here for new patients with red flags or potential secondary causes. Furthermore, this technique does not appear to be very beneficial for identification of probable headache disorders, as the number of characteristics in RREF for probable headaches are significant. This follows with our caveat for secondary headaches: if one is unsure about the potential diagnosis, the short cut of using 19 questions should not be used.

Secondly, the 19 questions and 63 characteristics yield an important theoretical concept: since the matrix can be reduced to linear combinations of 63 pivot/vectors of characteristics, this means that definite (not probable) diagnosis of headaches lives in 63-dimensional vector space. This observation allows for a theoretical framework for measuring the complexity of ICHD3, or any classification paradigm for that matter, and allows for the potential of reimaging of headaches as a Euclidean mathematical phenomenon.

Finally, we believe that envisioning the 63 basis characteristics as 19 questions has some empirical justification in clinical practice. In typical instructions for headache history taking in both the US and Europe, as well as standardized headache intake questionnaires in the United States, many incorporate questions similar to our 19 questions (21–23). We believe our clinical contribution here is not so much a reaffirming of the standard history taking paradigm, but a suggestion to also ask questions that are not commonly included in intake forms–such as precise date of onset, features of nummular headaches, as well as specific kinds of triggers. Doing so may allow for improved diagnostic accuracy for less common headache disorders. Of course, an important limitation is that we should verify the effectiveness of our 19 questions in clinical practice.

## Limitations and future directions

Firstly, our project is a study of the classification of headache disorder as it stands and not the studying of the disorders themselves. As such, we recommend readers take care to differentiate insights on headache classifications from our manuscript from clinical insights. Of course, how headache specialists interpret headache cannot be separated from the disease entity themselves–one of our goals in this manuscript is to point out precisely what may be the discordance between our clinical experience and the implicit assumptions of the structure of classification: this is best exemplified in the case of our hypothesis involving NDPH vs. migraine and TTH above.

Similarly, our observation of “fragmentations” in disorders (such as trigeminal autonomic cephalalgia) is only a representation of “fragmentation” in our interpretation of those disorders as an entity. Whether this is an actual differentiation pathophysiologically is a separate question. However, we should note that how we view disorders through a classification guideline necessarily influences how we study the actual disorder. For example, the conceptualization of episodic cluster headaches as its own disease entity allows for the discovery and generation of efficacious targeted therapy– viz high dosing of galcanezumab. Conversely, unifying TACs by frequency/timing of attacks rather than indomethacin responsiveness implies that paroxysmal hemicrania and hemicrania continua are often studied as separate disease entities, rightly or wrongly.

Our matrix representation is not intended to usurp clinical judgement. The existence of secondary headaches makes the prior statement particularly important. Indeed, we have intentionally not included secondary headaches in our matrix so that our algorithm may not be used in this fashion. We do not believe the world and all of its circumstances fit into a 63 dimensional, or any dimensional vector space. Yet that is precisely the nature of secondary headaches—the potential of inflicting damage from an external source to the brain to trigger headache is not likely to be countably finite. As such, all secondary headaches should be ruled out carefully. This is of course, not to suggest that secondary headaches do not contain their own order and structure. Indeed, differential diagnosis of primary and secondary headaches can be interpreted as a graph as well as through set/subset relationships based in order theory; these two observations are discussed in two separate papers (12, 15).

While we are confident that our matrix represents the ICHD3 faithfully, translation of any kind–including those from criteria to logical statements—involves interpretation on behalf of the encoder. So future users of our matrix should take it upon themselves to verify vital points of interpretation for their particular use case. For example, ICHD3 can be vague logically, using phrases such as “and/or” in criteria, such as “nausea and/or vomiting” in migraine. The phrase “and/or” is, of course, impossible to translate logically since it literally means “AND” OR “OR.” For migraine, for example, “nausea and/or vomiting” is considered 1 criterium in our matrix; yet we do not think it would be incorrect to encode “nausea” as a distinct entity as “vomiting” and combine both in the matrix through either an AND or an OR. Doing so, of course, would dramatically expand the number of rows in the matrix; however, in future iterations of headache classification, this may prove to be an important distinction. Alternatively, in specific use cases, such as advancing subclassifications of cyclical vomiting, for example, a distinction between nausea vs. vomiting may prove to be useful.

Similarly, cases can be made to combine pathognomonic characteristics linked by an AND operator as simply one larger characteristic. For example, as seen in nummular headaches, neither expansion nor inflation at extreme values are able to separate the 4 cardinal characteristics of the disease linked by AND. If so, one may choose to unify all of them under the same characteristics without fundamentally modifying the structure of the network.

In a sense, our exclusion of complications of migraine and “episodic syndromes associated with migraine from our matrix is a variant of the above observations: our editorial choice here is not likely to influence the result on a macroscopic level–how migraine structurally relates to cluster structurally and how our automated diagnosis separate between the two, for example, has little to do with subclassification of migraine's complications. However, the potential future inclusion of the above subclassifications will, of course, be vital to those researchers who studies cyclical vomiting. We advise users who studies these particular disorders to modify our matrix for their purpose.

In summary, translation from text to logical structures, just as interpretation of the classification itself, inherently contains hermeneutic choices that are unavoidable. Yet we do not think that interpretation, simply because it contains subjective nature, needs to be viewed undesirably. Indeed, following Hans-Georg Gadamer, we believe that hermeneutic problems in both the sciences and humanities to be the fundamental nature, the condition of possibilities, of human understanding (18). The answer, as Heidegger would say, is to enter into the hermeneutic circle correctly–which, in our case, resides with the user of our methodology and the matrix/logical/graphical interpretation they generate.

Finally, future direction to our project may include incorporating techniques in judging whether bipartite projections are statistically significant–either by comparing it to different forms of random graph (i.e., Erdos–Rényi-Gilbert model, configuration models, or exponential random graph model) or to adopt novel techniques in graph/network analysis (19).

## Conclusions

The mathematical embodiment of headache classification as a matrix representation offers us three potential benefits: (1) the large-scale systematic investigations of relationships between characteristics of headaches and phenotypes, (2) the graphical representation and analysis of these characteristics and phenotypes, and (3) the potential improvement in clinical diagnosis of headaches both by automation and elimination of redundancy in history taking.

## Data Availability

The original contributions presented in the study are included in the article/supplementary material, further inquiries can be directed to the corresponding author.
