# Supplementary material for: Matrix and graphical representation of the primary headache syndromes in the International Classification of Headache Disorders (ICHD3): a basis for automated diagnosis and analysis of criteria
Source: Front Neurol. 2026 May 11;17:1812996. doi: 10.3389/fneur.2026.1812996 (PMC13200560; doi:10.3389/fneur.2026.1812996)
Supplement: Supplementary file 8 [file Data_Sheet_8.pdf]

In the supplement, we include the headache phenotype encoded as a vector for each of the patients (data sheet 1.csv, data sheet 2.csv, data sheet 3.csv) and the result of the matrix multiplication (data sheet 4.csv, data sheet 5.csv, data sheet 6.csv), all in CSV format. An CSV file entitled data sheet 14.csv (corresponding to the notation Sk in the proof), shows the row sum of the original matrix with each row's phenotype labeled. We copied the corresponding row of Sk into each output file for easy verification: each output file is labeled with the first column being the result of the matrix multiplication, the second column being the Sk label, the third column being the row sum of the Sk and the fourth column yielding TRUE or FALSE depending on whether the first and third column agree for that row.

The descriptions of the cases are as follows:

Example case 1:

This is a case of migraine without aura where the presenting symptoms are photophobia, nausea, no phonophobia in a patient with unilateral headaches lasting 4 to 6 hours that can be aggravated with activities. The headache is not of pulsating quality. The pain is moderate to severe. There have been more than 5 attacks.

In this case, 1 is placed for the following characteristics and 0 elsewhere: "photophobia", "nausea and/or vomiting", "no phonophobia", "unilateral", "4 to 72 hours duration", "moderate to severe", "nonpulsating", "aggravated with activities", "greater than 5 episodes". This is reflected in data sheet 1.csv.

Here, the output matrix matches Sk at "migraine w/o aura1", "migraine w/o aura3", "migraine w/o aura5", "migraine w/o aura8". This is due to the fact that there are multiple ways that the above case satisfies the logical conditions of migraine without aura. Furthermore, it also satisfies

a number of “probable migraine” diagnoses; this is to be expected since any firm diagnosis of a disorder automatically satisfies the weaker criteria for probable diagnosis of the same. Noticed that this case does not satisfy any other headache diagnoses. Therefore, the diagnosis is migraine without aura.

Example case 2:

This is a case of infrequent tension-type headache where the presenting symptoms are no photophobia, no phonophobia, no nausea and/or vomiting, lasting 60 minutes, with bilateral mild to moderate headaches, that is not aggravated by physical activity. It is pulsating. More than 10 episodes has occurred and also less than 12 days per year.

In this case, 1 is placed for the following characteristics and 0 elsewhere: “no photophobia”, “no phonophobia”, “no nausea and/or vomiting”, “30 minutes to 7 days in duration”, “more than 10 episodes”, “less than 12 days per year”, “not aggravated by physical activity”, “pulsating”, “mild to moderate pain”. This is reflected in data sheet 2.csv.

Here, the output matrix matches Sk at “infrequent tension type headache9” and “infrequent tension type headache10”. This similarly satisfies a number of probable infrequent tension type headache phenotypes. The diagnosis is tension type headache. Again, notice that this does not satisfy any other headache disorders.

Example case 3:

This is a case of episodic cluster headache where the presenting symptom is restlessness in a patient who has 2 hours of unilateral headaches with orbital/supraorbital pain. More than 5 attacks have occurred and it is severe. It occurs every other day.

In this case, 1 is placed for the following characteristics and 0 elsewhere: “restless”, “15 to 180 minutes”, “severe”, “every other day to 8 per day”, “greater than 5 episodes”, “orbital or supraorbital or temporal pain”, “unilateral”. This is reflected in data sheet 3.csv.

Here, the output matrix matches Sk at “cluster8” as well as probable cluster headache diagnoses. The diagnosis is therefore cluster headaches.
