# Supplementary material for: Matrix and graphical representation of the primary headache syndromes in the International Classification of Headache Disorders (ICHD3): a basis for automated diagnosis and analysis of criteria
Source: Front Neurol. 2026 May 11;17:1812996. doi: 10.3389/fneur.2026.1812996 (PMC13200560; doi:10.3389/fneur.2026.1812996)
Supplement: Supplementary file 9 [file Data_Sheet_9.pdf]

```
expansion max out at 3 when given inflation of 3.3

expansion: 2 modularity: 0.7104363643165259
expansion: 3 modularity: 0.7135201862918308
expansion: 4 modularity: 0.6016468871868201
expansion: 5 modularity: 0.4618566993051382
expansion: 6 modularity: 0.06993542444051558
expansion: 7 modularity: 0.05823957953408398
expansion: 8 modularity: 0.05823957953408398
expansion: 9 modularity: 0.050892414592862235
```
