# Supplementary material for: Matrix and graphical representation of the primary headache syndromes in the International Classification of Headache Disorders (ICHD3): a basis for automated diagnosis and analysis of criteria
Source: Front Neurol. 2026 May 11;17:1812996. doi: 10.3389/fneur.2026.1812996 (PMC13200560; doi:10.3389/fneur.2026.1812996)
Supplement: Supplementary file 10 [file Data_Sheet_10.pdf]

Max at 3.3. See below.

```
inflation: 1.5 modularity: 0.0699354244405152
inflation: 1.6 modularity: 0.0699354244405152
inflation: 1.7 modularity: 0.6034670201722652
inflation: 1.8 modularity: 0.6232149370142689
inflation: 1.9 modularity: 0.6318830719449335
inflation: 2.0 modularity: 0.65680848974554
inflation: 2.1 modularity: 0.6947776094417828
inflation: 2.2 modularity: 0.6967251634261894
inflation: 2.3 modularity: 0.6999364064005144
inflation: 2.4 modularity: 0.7001889103984923
inflation: 2.5 modularity: 0.7011194343910362
inflation: 2.6 modularity: 0.7013462203892202
inflation: 2.7 modularity: 0.7017986233856186
inflation: 2.8 modularity: 0.7014023323887965
inflation: 2.9 modularity: 0.7014561063883589
inflation: 3.0 modularity: 0.7076892143385045
inflation: 3.1 modularity: 0.707250839342011
inflation: 3.2 modularity: 0.7065926923472694
inflation: 3.3 modularity: 0.7104363643165259
inflation: 3.4 modularity: 0.7045749983633862
inflation: 3.5 modularity: 0.6947846234417192
inflation: 3.6 modularity: 0.671546072627624
inflation: 3.7 modularity: 0.6648114636815018
inflation: 3.8 modularity: 0.6549848497601147
inflation: 3.9 modularity: 0.6520857297833021
inflation: 4.0 modularity: 0.6460536898315583
inflation: 4.1 modularity: 0.6431896398544757
inflation: 4.2 modularity: 0.6386796378905557
inflation: 4.3 modularity: 0.6280090059759158
inflation: 4.4 modularity: 0.6248784240009577
inflation: 4.5 modularity: 0.6230068550159509
inflation: 4.6 modularity: 0.6196658530426796
inflation: 4.7 modularity: 0.6177054400583647
inflation: 4.8 modularity: 0.614398339084821
inflation: 4.9 modularity: 0.6097422121220687
inflation: 5.0 modularity: 0.6082470611340297
inflation: 5.1 modularity: 0.606253916149976
inflation: 5.2 modularity: 0.6040900971672859
inflation: 5.3 modularity: 0.6020279811837732
inflation: 5.4 modularity: 0.5993135632054921
inflation: 5.5 modularity: 0.5969381552244933
```
