# Supplementary material for: Matrix and graphical representation of the primary headache syndromes in the International Classification of Headache Disorders (ICHD3): a basis for automated diagnosis and analysis of criteria
Source: Front Neurol. 2026 May 11;17:1812996. doi: 10.3389/fneur.2026.1812996 (PMC13200560; doi:10.3389/fneur.2026.1812996)
Supplement: Supplementary file 11 [file Data_Sheet_11.pdf]

Parameter exploration for inflation = 1.5 and expansion = 2

cluster_16
retinal aura
speech and/or language aura
the aura is accompanied, or followed within 60 minutes, by headache
greater than 15 days per month
1 to 600 seconds
motor aura
fully reversible
visual aura
sensory aura
each individual aura symptom lasts 5–60 minutes
at least one aura symptom spreads gradually over 5 minutes
brainstem aura
two or more aura symptoms occur in succession
greater than 8 days per month
at least one aura symptom is positive
relieve by triptan or ergot
at least one aura symptom is unilateral

cluster_17
15 minutes up to four hours after waking

cluster_29
conjunctival injection
greater than 1 per day
greater than 5 episodes
nausea/vomiting
pulsating
moderate to severe
greater than 5 per day
greater than 20 episodes
orbital or supraorbital or temporal pain

cluster_15
more than 10 episodes
less than 12 days per year
no nausea/vomiting
1 to 14 days per month
no photophobia
not aggrevated by activity
no phonophobia
bilateral location
hours to days
nonpulsating
30 min to 7 days in duration
mild to moderate pain

cluster_14
up to 72 hours with mild
brought on by sex
increasing in intensity with increasing sexual excitement
1 minute to 72 hours with severe
abrupt explosive intensity just before or with orgasm

cluster_28
unremitting within 24 hours
constant
clearly remembered onset

cluster_10
hypnic3
hypnic9
hypnic8
stabbing5
hypnic6
hypnic4
stabbing2
stabbing3
hypnic5
hypnic1
hypnic7
stabbing1
stabbing7
stabbing8
stabbing6
hypnic2
stabbing4

cluster_38
miosis

cluster_39
more than 1 episode per day

cluster_11
nummular1

cluster_13
Probable Chronic Tension Type Headache78
Probable Infrequent Episodic Tension Type Headache8
Probable Chronic Tension Type Headache13
Probable Frequent Episodic Tension Type Headache29
Probable Chronic Tension Type Headache101
chronic tension type headache11
Probable Chronic Tension Type Headache60
Probable Infrequent Episodic Tension Type Headache26
Probable Chronic Tension Type Headache71

frequent tension type headache6
Probable Frequent Episodic Tension Type Headache4
chronic tension type headache22
Probable Chronic Tension Type Headache45
Probable Infrequent Episodic Tension Type Headache55
Probable Chronic Tension Type Headache100
Probable Infrequent Episodic Tension Type Headache1
Probable Frequent Episodic Tension Type Headache68
Probable Chronic Tension Type Headache127
Probable Chronic Tension Type Headache44
Probable Infrequent Episodic Tension Type Headache15
Probable Chronic Tension Type Headache29
Probable Chronic Tension Type Headache62
Probable Infrequent Episodic Tension Type Headache54
Probable Chronic Tension Type Headache31
Probable Chronic Tension Type Headache19
Probable Infrequent Episodic Tension Type Headache14
Probable Chronic Tension Type Headache49
Probable Chronic Tension Type Headache14
chronic tension type headache6
Probable Frequent Episodic Tension Type Headache75
Probable Chronic Tension Type Headache82
Probable Chronic Tension Type Headache87
Probable Chronic Tension Type Headache58
Probable Infrequent Episodic Tension Type Headache23
chronic tension type headache9
Probable Infrequent Episodic Tension Type Headache57
Probable Infrequent Episodic Tension Type Headache75
chronic tension type headache32
Probable Infrequent Episodic Tension Type Headache24
infrequent tension type headache21
Probable Chronic Tension Type Headache18
Probable Frequent Episodic Tension Type Headache77
chronic tension type headache37
Probable Infrequent Episodic Tension Type Headache25
frequent tension type headache18
Probable Infrequent Episodic Tension Type Headache38
Probable Chronic Tension Type Headache124
Probable Frequent Episodic Tension Type Headache9
chronic tension type headache30
Probable Frequent Episodic Tension Type Headache70
Probable Chronic Tension Type Headache55
frequent tension type headache20
Probable Chronic Tension Type Headache72
Probable Infrequent Episodic Tension Type Headache73
chronic tension type headache10
Probable Infrequent Episodic Tension Type Headache19
Probable Infrequent Episodic Tension Type Headache41
frequent tension type headache4
chronic tension type headache20

chronic tension type headache19
Probable Chronic Tension Type Headache81
Probable Frequent Episodic Tension Type Headache20
Probable Infrequent Episodic Tension Type Headache74
frequent tension type headache7
chronic tension type headache18
frequent tension type headache9
frequent tension type headache22
chronic tension type headache1
frequent tension type headache19
Probable Frequent Episodic Tension Type Headache47
Probable Frequent Episodic Tension Type Headache33
Probable Frequent Episodic Tension Type Headache7
Probable Chronic Tension Type Headache73
Probable Frequent Episodic Tension Type Headache71
frequent tension type headache8
Probable Infrequent Episodic Tension Type Headache47
Probable Chronic Tension Type Headache66
chronic tension type headache12
Probable Chronic Tension Type Headache112
Probable Frequent Episodic Tension Type Headache40
Probable Chronic Tension Type Headache102
chronic tension type headache17
infrequent tension type headache3
frequent tension type headache21
Probable Chronic Tension Type Headache110
Probable Infrequent Episodic Tension Type Headache45
Probable Frequent Episodic Tension Type Headache56
Probable Frequent Episodic Tension Type Headache5
Probable Chronic Tension Type Headache4
Probable Frequent Episodic Tension Type Headache30
Probable Chronic Tension Type Headache83
Probable Chronic Tension Type Headache42
chronic tension type headache5
Probable Frequent Episodic Tension Type Headache44
Probable Infrequent Episodic Tension Type Headache69
Probable Chronic Tension Type Headache7
Probable Chronic Tension Type Headache99
Probable Chronic Tension Type Headache20
Probable Chronic Tension Type Headache76
Probable Frequent Episodic Tension Type Headache23
Probable Frequent Episodic Tension Type Headache24
Probable Chronic Tension Type Headache36
Probable Chronic Tension Type Headache91
chronic tension type headache8
Probable Frequent Episodic Tension Type Headache2
Probable Frequent Episodic Tension Type Headache57
Probable Chronic Tension Type Headache109
Probable Chronic Tension Type Headache23
chronic tension type headache33

infrequent tension type headache10
Probable Chronic Tension Type Headache27
Probable Chronic Tension Type Headache114
Probable Chronic Tension Type Headache86
Probable Infrequent Episodic Tension Type Headache28
Probable Frequent Episodic Tension Type Headache18
Probable Frequent Episodic Tension Type Headache54
Probable Infrequent Episodic Tension Type Headache21
Probable Chronic Tension Type Headache107
frequent tension type headache17
Probable Chronic Tension Type Headache105
chronic tension type headache27
infrequent tension type headache13
Probable Chronic Tension Type Headache108
Probable Infrequent Episodic Tension Type Headache49
Probable Frequent Episodic Tension Type Headache31
Probable Chronic Tension Type Headache9
Probable Chronic Tension Type Headache84
Probable Infrequent Episodic Tension Type Headache61
Probable Chronic Tension Type Headache129
Probable Chronic Tension Type Headache104
Probable Chronic Tension Type Headache74
Probable Frequent Episodic Tension Type Headache34
Probable Chronic Tension Type Headache3
Probable Chronic Tension Type Headache17
Probable Infrequent Episodic Tension Type Headache60
Probable Infrequent Episodic Tension Type Headache67
Probable Chronic Tension Type Headache70
Probable Frequent Episodic Tension Type Headache13
Probable Chronic Tension Type Headache131
Probable Frequent Episodic Tension Type Headache36
Probable Frequent Episodic Tension Type Headache32
Probable Chronic Tension Type Headache118
Probable Chronic Tension Type Headache34
chronic tension type headache14
Probable Frequent Episodic Tension Type Headache19
infrequent tension type headache17
chronic tension type headache23
Probable Frequent Episodic Tension Type Headache58
Probable Frequent Episodic Tension Type Headache63
Probable Infrequent Episodic Tension Type Headache56
Probable Chronic Tension Type Headache40
Probable Infrequent Episodic Tension Type Headache40
chronic tension type headache21
Probable Frequent Episodic Tension Type Headache35
Probable Chronic Tension Type Headache68
Probable Chronic Tension Type Headache11
Probable Infrequent Episodic Tension Type Headache70
Probable Infrequent Episodic Tension Type Headache13
frequent tension type headache16

Probable Chronic Tension Type Headache24
Probable Infrequent Episodic Tension Type Headache39
Probable Frequent Episodic Tension Type Headache52
infrequent tension type headache8
Probable Frequent Episodic Tension Type Headache49
Probable Chronic Tension Type Headache77
infrequent tension type headache9
chronic tension type headache3
Probable Infrequent Episodic Tension Type Headache53
Probable Chronic Tension Type Headache94
Probable Chronic Tension Type Headache53
Probable Chronic Tension Type Headache52
Probable Chronic Tension Type Headache117
infrequent tension type headache15
Probable Infrequent Episodic Tension Type Headache46
Probable Frequent Episodic Tension Type Headache76
Probable Chronic Tension Type Headache121
Probable Frequent Episodic Tension Type Headache37
Probable Frequent Episodic Tension Type Headache25
chronic tension type headache43
Probable Chronic Tension Type Headache93
Probable Chronic Tension Type Headache30
infrequent tension type headache16
Probable Infrequent Episodic Tension Type Headache10
Probable Chronic Tension Type Headache106
Probable Chronic Tension Type Headache126
Probable Infrequent Episodic Tension Type Headache36
Probable Infrequent Episodic Tension Type Headache22
Probable Chronic Tension Type Headache47
Probable Chronic Tension Type Headache22
infrequent tension type headache4
Probable Frequent Episodic Tension Type Headache48
Probable Frequent Episodic Tension Type Headache69
infrequent tension type headache14
Probable Chronic Tension Type Headache41
Probable Chronic Tension Type Headache16
infrequent tension type headache18
Probable Frequent Episodic Tension Type Headache16
chronic tension type headache28
Probable Infrequent Episodic Tension Type Headache71
Probable Chronic Tension Type Headache111
Probable Chronic Tension Type Headache130
Probable Chronic Tension Type Headache61
Probable Chronic Tension Type Headache95
Probable Infrequent Episodic Tension Type Headache44
Probable Chronic Tension Type Headache122
frequent tension type headache1
Probable Chronic Tension Type Headache65
Probable Frequent Episodic Tension Type Headache42
Probable Chronic Tension Type Headache5

Probable Chronic Tension Type Headache125
Probable Infrequent Episodic Tension Type Headache62
Probable Infrequent Episodic Tension Type Headache51
Probable Chronic Tension Type Headache43
Probable Chronic Tension Type Headache38
chronic tension type headache41
Probable Infrequent Episodic Tension Type Headache37
Probable Chronic Tension Type Headache120
frequent tension type headache5
Probable Frequent Episodic Tension Type Headache6
Probable Infrequent Episodic Tension Type Headache64
Probable Frequent Episodic Tension Type Headache51
Probable Chronic Tension Type Headache116
chronic tension type headache24
Probable Chronic Tension Type Headache67
Probable Chronic Tension Type Headache8
Probable Infrequent Episodic Tension Type Headache50
Probable Infrequent Episodic Tension Type Headache33
Probable Chronic Tension Type Headache63
Probable Chronic Tension Type Headache28
Probable Frequent Episodic Tension Type Headache50
Probable Chronic Tension Type Headache51
Probable Chronic Tension Type Headache48
chronic tension type headache7
Probable Frequent Episodic Tension Type Headache45
Probable Infrequent Episodic Tension Type Headache17
Probable Infrequent Episodic Tension Type Headache12
Probable Infrequent Episodic Tension Type Headache66
Probable Frequent Episodic Tension Type Headache15
Probable Infrequent Episodic Tension Type Headache58
Probable Infrequent Episodic Tension Type Headache5
Probable Chronic Tension Type Headache64
Probable Chronic Tension Type Headache123
chronic tension type headache34
Probable Frequent Episodic Tension Type Headache60
Probable Frequent Episodic Tension Type Headache43
Probable Chronic Tension Type Headache37
Probable Chronic Tension Type Headache88
Probable Chronic Tension Type Headache59
Probable Chronic Tension Type Headache79
Probable Frequent Episodic Tension Type Headache64
Probable Frequent Episodic Tension Type Headache66
Probable Chronic Tension Type Headache57
Probable Infrequent Episodic Tension Type Headache48
Probable Frequent Episodic Tension Type Headache53
Probable Infrequent Episodic Tension Type Headache31
frequent tension type headache14
Probable Chronic Tension Type Headache2
infrequent tension type headache5
chronic tension type headache39

frequent tension type headache13
Probable Infrequent Episodic Tension Type Headache20
infrequent tension type headache2
Probable Infrequent Episodic Tension Type Headache65
chronic tension type headache15
chronic tension type headache16
Probable Frequent Episodic Tension Type Headache8
frequent tension type headache10
Probable Infrequent Episodic Tension Type Headache72
Probable Chronic Tension Type Headache97
Probable Frequent Episodic Tension Type Headache11
Probable Infrequent Episodic Tension Type Headache11
Probable Infrequent Episodic Tension Type Headache16
chronic tension type headache35
infrequent tension type headache12
Probable Frequent Episodic Tension Type Headache74
Probable Frequent Episodic Tension Type Headache12
chronic tension type headache25
frequent tension type headache3
Probable Chronic Tension Type Headache75
Probable Chronic Tension Type Headache10
infrequent tension type headache1
chronic tension type headache31
Probable Infrequent Episodic Tension Type Headache77
Probable Chronic Tension Type Headache92
Probable Infrequent Episodic Tension Type Headache18
infrequent tension type headache11
Probable Infrequent Episodic Tension Type Headache68
Probable Chronic Tension Type Headache85
infrequent tension type headache6
Probable Infrequent Episodic Tension Type Headache29
Probable Frequent Episodic Tension Type Headache26
chronic tension type headache38
Probable Chronic Tension Type Headache46
Probable Chronic Tension Type Headache33
Probable Chronic Tension Type Headache90
Probable Infrequent Episodic Tension Type Headache7
Probable Chronic Tension Type Headache26
Probable Infrequent Episodic Tension Type Headache59
Probable Infrequent Episodic Tension Type Headache27
chronic tension type headache42
Probable Infrequent Episodic Tension Type Headache34
Probable Chronic Tension Type Headache1
Probable Frequent Episodic Tension Type Headache61
frequent tension type headache12
chronic tension type headache44
Probable Frequent Episodic Tension Type Headache55
Probable Frequent Episodic Tension Type Headache72
Probable Infrequent Episodic Tension Type Headache4
infrequent tension type headache19

Probable Infrequent Episodic Tension Type Headache2
infrequent tension type headache22
Probable Chronic Tension Type Headache115
frequent tension type headache15
Probable Frequent Episodic Tension Type Headache28
Probable Frequent Episodic Tension Type Headache46
Probable Chronic Tension Type Headache128
chronic tension type headache13
Probable Chronic Tension Type Headache113
Probable Chronic Tension Type Headache103
Probable Infrequent Episodic Tension Type Headache35
Probable Chronic Tension Type Headache12
frequent tension type headache2
Probable Infrequent Episodic Tension Type Headache76
Probable Infrequent Episodic Tension Type Headache9
Probable Frequent Episodic Tension Type Headache39
Probable Infrequent Episodic Tension Type Headache63
chronic tension type headache26
Probable Chronic Tension Type Headache25
Probable Chronic Tension Type Headache119
Probable Chronic Tension Type Headache69
Probable Chronic Tension Type Headache50
Probable Chronic Tension Type Headache80
Probable Frequent Episodic Tension Type Headache65
Probable Chronic Tension Type Headache35
Probable Chronic Tension Type Headache96
chronic tension type headache36
Probable Infrequent Episodic Tension Type Headache32
Probable Frequent Episodic Tension Type Headache38
Probable Frequent Episodic Tension Type Headache27
chronic tension type headache4
Probable Frequent Episodic Tension Type Headache73
Probable Infrequent Episodic Tension Type Headache30
Probable Frequent Episodic Tension Type Headache1
Probable Frequent Episodic Tension Type Headache10
Probable Frequent Episodic Tension Type Headache41
infrequent tension type headache20
Probable Frequent Episodic Tension Type Headache17
Probable Infrequent Episodic Tension Type Headache43
Probable Chronic Tension Type Headache21
infrequent tension type headache7
Probable Frequent Episodic Tension Type Headache21
frequent tension type headache11
Probable Chronic Tension Type Headache56
Probable Chronic Tension Type Headache89
Probable Infrequent Episodic Tension Type Headache6
Probable Chronic Tension Type Headache15
Probable Chronic Tension Type Headache54
Probable Frequent Episodic Tension Type Headache59
chronic tension type headache29

Probable Frequent Episodic Tension Type Headache14
Probable Chronic Tension Type Headache6
Probable Infrequent Episodic Tension Type Headache3
chronic tension type headache40
Probable Frequent Episodic Tension Type Headache22
Probable Chronic Tension Type Headache132
chronic tension type headache2
Probable Frequent Episodic Tension Type Headache67
Probable Infrequent Episodic Tension Type Headache52
Probable Chronic Tension Type Headache98
Probable Frequent Episodic Tension Type Headache62
Probable Chronic Tension Type Headache32
Probable Frequent Episodic Tension Type Headache3
Probable Infrequent Episodic Tension Type Headache42
Probable Chronic Tension Type Headache39

cluster_12
cough2
cough1

cluster_49
no orbital or supraorbital or temporal pain

cluster_6
traction1

cluster_7
Probable Cluster Headache36
Probable Hemicrania Continua22
sun1
Probable Cluster Headache16
Probable Cluster Headache10
Probable Hemicrania Continua27
ph8
hc7
Probable Cluster Headache11
Probable Cluster Headache29
Probable Hemicrania Continua18
Probable Cluster Headache22
Probable Cluster Headache37
Probable Cluster Headache25
Probable Hemicrania Continua9
Probable Cluster Headache20
hc5
Probable Hemicrania Continua21
Probable Hemicrania Continua13
Probable Hemicrania Continua19
Probable Cluster Headache6
hc4
Probable Hemicrania Continua5

cluster6
ph9
Probable Cluster Headache23
Probable Cluster Headache27
Probable Hemicrania Continua28
cluster3
hc2
Probable Cluster Headache1
cluster7
Probable Hemicrania Continua16
cluster8
ph4
Probable Cluster Headache28
Probable Hemicrania Continua14
hc9
Probable Hemicrania Continua25
Probable Cluster Headache32
hc1
sun7
sun5
Probable Hemicrania Continua26
Probable Hemicrania Continua4
Probable Hemicrania Continua10
sun6
Probable Hemicrania Continua7
Probable Cluster Headache13
Probable Cluster Headache7
hc8
Probable Cluster Headache2
Probable Hemicrania Continua6
Probable Cluster Headache34
Probable Cluster Headache9
Probable Hemicrania Continua24
Probable Cluster Headache26
ph6
Probable Cluster Headache14
cluster2
Probable Hemicrania Continua3
Probable Cluster Headache35
sun2
ph2
cluster4
ph1
hc6
Probable Hemicrania Continua12
cluster1
ph5
Probable Hemicrania Continua11
Probable Hemicrania Continua17
Probable Hemicrania Continua1

Probable Cluster Headache31
Probable Hemicrania Continua15
Probable Cluster Headache4
Probable Hemicrania Continua20
Probable Hemicrania Continua23
ph3
Probable Cluster Headache24
Probable Hemicrania Continua8
Probable Cluster Headache15
Probable Cluster Headache17
Probable Cluster Headache18
sun4
Probable Cluster Headache12
Probable Hemicrania Continua2
Probable Cluster Headache30
Probable Cluster Headache8
Probable Cluster Headache5
Probable Cluster Headache19
sun8
hc3
Probable Cluster Headache21
Probable Cluster Headache33
sun3
cluster5
Probable Cluster Headache3
ph7
cluster9

cluster_48
no nasal congestion

cluster_5
compression1

cluster_4
coldHA1

cluster_0
cm279
probable migraine without aura32
 migraine w/o aura4
probable migraine without aura65
probable migraine without aura47
probable migraine without aura25
probable migraine without aura51
probable migraine without aura18
cm20
probable migraine without aura46
cm153
cm235

probable migraine without aura31
probable migraine without aura20
probable migraine without aura73
cm21
 migraine w/o aura21
probable migraine without aura7
probable migraine without aura57
 migraine w/o aura8
probable migraine without aura58
probable migraine without aura54
probable migraine without aura76
probable migraine without aura48
cm13
probable migraine without aura59
probable migraine without aura39
 migraine w/o aura13
cm9
cm92
probable migraine without aura1
probable migraine without aura21
probable migraine without aura71
probable migraine without aura62
 migraine w/o aura10
cm114
probable migraine without aura52
probable migraine without aura8
probable migraine without aura40
probable migraine without aura77
cm259
probable migraine without aura72
probable migraine without aura44
probable migraine without aura66
probable migraine without aura49
probable migraine without aura3
probable migraine without aura10
cm236
cm10
probable migraine without aura24
cm97
probable migraine without aura16
 migraine w/o aura19
 migraine w/o aura22
probable migraine without aura28
cm93
cm130
probable migraine without aura75
probable migraine without aura33
probable migraine without aura35
probable migraine without aura2
cm11

cm284
migraine w/o aura1
cm128
probable migraine without aura4
cm31
cm94
probable migraine without aura29
cm109
probable migraine without aura9
migraine w/o aura2
cm4
probable migraine without aura67
cm6
cm1
probable migraine without aura19
probable migraine without aura55
migraine w/o aura9
cm7
cm156
migraine w/o aura11
cm24
probable migraine without aura43
migraine w/o aura3
cm3
cm157
probable migraine without aura50
probable migraine without aura27
probable migraine without aura64
cm28
probable migraine without aura70
migraine w/o aura15
migraine w/o aura6
migraine w/o aura12
probable migraine without aura68
probable migraine without aura13
probable migraine without aura53
probable migraine without aura38
probable migraine without aura37
probable migraine without aura60
probable migraine without aura61
probable migraine without aura69
probable migraine without aura14
cm14
migraine w/o aura20
cm2
migraine w/o aura14
probable migraine without aura45
migraine w/o aura7
probable migraine without aura74
probable migraine without aura17

cm155
probable migraine without aura23
probable migraine without aura41
probable migraine without aura11
probable migraine without aura42
cm15
 migraine w/o aura18
cm103
probable migraine without aura22
probable migraine without aura6
cm5
probable migraine without aura36
cm23
probable migraine without aura12
cm25
cm84
cm226
probable migraine without aura63
cm12
cm8
 migraine w/o aura5
probable migraine without aura30
probable migraine without aura56
 migraine w/o aura17
probable migraine without aura26
cm166
cm227
 migraine w/o aura16
probable migraine without aura5
probable migraine without aura15
probable migraine without aura34

cluster_1
ndph1

cluster_3
thunderclap1

cluster_2
exercise1

cluster_54
ptosis

cluster_40
more than 3 months

cluster_41
more than 10 days per month

cluster_55
restless

cluster_43
single or series of stabs
no conjunctival injection
no restless
up to few seconds

cluster_56
rhinorrhea

cluster_42
nasal congestion

cluster_46
no lacrimation

cluster_52
phonophobia

cluster_9
sex3
sex1
sex4
sex2

cluster_8
cm52
cm16
cm172
cm238
mwa5
probable migraine with aura83
probable migraine with aura65
mwa96
probable migraine with aura41
probable migraine with aura7
cm256
probable migraine with aura14
cm230
probable migraine with aura120
mwa91
mwa25
probable migraine with aura10
mwa45
probable migraine with aura40
mwa112
cm165
probable migraine with aura68

cm143
mwa14
mwa107
probable migraine with aura80
probable migraine with aura129
probable migraine with aura71
mwa71
cm163
probable migraine with aura58
probable migraine with aura62
cm160
probable migraine with aura76
probable migraine with aura92
mwa75
probable migraine with aura118
probable migraine with aura82
cm111
probable migraine with aura29
cm213
mwa109
cm106
cm18
mwa88
cm129
cm273
probable migraine with aura11
probable migraine with aura136
probable migraine with aura140
mwa66
probable migraine with aura36
probable migraine with aura85
cm276
cm72
probable migraine with aura124
cm152
probable migraine with aura70
cm150
cm266
cm76
cm133
cm277
cm250
cm121
cm248
cm145
cm173
mwa61
mwa68
mwa24
probable migraine with aura94

probable migraine with aura101
cm87
cm95
mwa15
cm27
cm282
probable migraine with aura23
probable migraine with aura52
probable migraine with aura79
probable migraine with aura104
cm219
mwa10
cm189
cm100
probable migraine with aura31
mwa105
cm42
cm191
cm242
cm119
cm147
probable migraine with aura109
mwa106
probable migraine with aura51
mwa21
cm251
cm137
probable migraine with aura143
cm220
cm159
mwa47
cm196
probable migraine with aura91
mwa59
cm176
probable migraine with aura6
cm69
mwa27
probable migraine with aura95
cm116
cm199
cm190
cm215
mwa34
cm79
mwa20
cm80
cm221
probable migraine with aura108
cm158

probable migraine with aura105
cm249
cm122
cm240
probable migraine with aura2
cm46
mwa6
mwa37
probable migraine with aura19
probable migraine with aura39
mwa74
cm56
cm65
cm134
cm260
cm22
cm261
mwa87
mwa16
cm281
cm168
cm71
mwa33
cm38
cm175
probable migraine with aura127
mwa119
cm62
cm19
mwa38
cm74
probable migraine with aura119
probable migraine with aura74
cm154
probable migraine with aura100
probable migraine with aura116
cm101
mwa113
mwa1
cm194
cm182
probable migraine with aura25
cm59
mwa13
mwa79
probable migraine with aura44
probable migraine with aura115
probable migraine with aura37
probable migraine with aura123
cm241

cm61
cm216
mwa50
cm139
probable migraine with aura126
probable migraine with aura145
probable migraine with aura87
mwa64
mwa18
cm258
cm183
mwa100
cm225
cm123
probable migraine with aura111
cm86
cm115
cm37
mwa117
cm107
cm136
cm50
cm268
cm57
cm124
cm207
mwa104
cm254
probable migraine with aura78
probable migraine with aura128
mwa72
cm68
mwa51
probable migraine with aura21
cm233
cm36
probable migraine with aura84
mwa22
mwa78
cm247
cm118
probable migraine with aura103
cm44
cm252
mwa63
probable migraine with aura53
probable migraine with aura117
cm85
probable migraine with aura114
probable migraine with aura142

mwa60
cm82
cm149
cm120
cm269
cm181
probable migraine with aura60
cm198
cm218
cm222
cm204
mwa65
mwa111
probable migraine with aura8
cm212
cm148
cm208
cm265
cm90
probable migraine with aura64
cm239
mwa70
cm217
mwa99
probable migraine with aura139
cm205
mwa54
cm30
cm141
probable migraine with aura107
probable migraine with aura56
mwa44
probable migraine with aura59
probable migraine with aura88
cm47
cm180
cm78
cm110
cm132
cm245
cm41
cm135
cm283
mwa41
probable migraine with aura110
probable migraine with aura5
mwa95
probable migraine with aura99
cm267
cm210

cm200
probable migraine with aura16
cm29
probable migraine with aura90
probable migraine with aura22
mwa42
mwa57
cm81
probable migraine with aura106
probable migraine with aura30
mwa3
cm98
cm187
cm169
cm243
cm237
probable migraine with aura113
cm77
cm35
cm64
probable migraine with aura61
mwa80
mwa90
cm99
mwa120
cm126
mwa8
mwa23
mwa93
cm70
probable migraine with aura72
cm188
cm17
mwa56
cm244
cm83
cm131
mwa17
cm224
cm264
cm164
probable migraine with aura125
cm223
probable migraine with aura89
probable migraine with aura20
mwa102
probable migraine with aura96
mwa69
mwa76
cm178

mwa114
cm105
cm209
cm255
cm231
mwa35
mwa98
cm193
mwa97
mwa101
probable migraine with aura4
probable migraine with aura9
cm151
cm75
cm177
cm161
mwa73
cm197
probable migraine with aura38
mwa89
probable migraine with aura69
cm140
mwa19
mwa29
cm43
cm195
cm274
mwa32
probable migraine with aura135
cm73
probable migraine with aura17
probable migraine with aura54
mwa82
probable migraine with aura43
cm89
probable migraine with aura35
cm54
cm45
mwa28
cm262
cm272
mwa116
cm246
cm211
cm162
cm202
cm263
cm60
probable migraine with aura1
probable migraine with aura18

mwa9
cm33
cm26
mwa26
cm146
mwa77
mwa53
cm127
cm214
mwa84
cm171
cm270
probable migraine with aura75
mwa62
probable migraine with aura13
cm192
cm174
mwa43
probable migraine with aura57
probable migraine with aura144
cm63
cm253
probable migraine with aura15
cm102
mwa49
mwa4
cm203
cm91
cm278
cm66
cm67
mwa81
cm96
probable migraine with aura47
mwa103
probable migraine with aura98
cm48
cm53
probable migraine with aura141
mwa2
cm117
cm144
cm108
cm55
cm228
cm104
probable migraine with aura55
cm32
cm186
cm40

mwa86
cm275
cm138
probable migraine with aura138
cm125
mwa30
probable migraine with aura12
mwa94
cm39
cm49
cm280
mwa83
mwa39
probable migraine with aura132
cm257
cm206
probable migraine with aura45
probable migraine with aura131
mwa58
probable migraine with aura133
mwa36
probable migraine with aura130
mwa67
mwa118
cm185
cm34
mwa52
mwa115
probable migraine with aura26
probable migraine with aura32
probable migraine with aura66
probable migraine with aura121
cm232
mwa48
cm170
probable migraine with aura3
cm271
mwa85
mwa46
cm58
cm234
cm51
cm88
probable migraine with aura42
probable migraine with aura73
probable migraine with aura137
mwa31
cm201
cm113
mwa7

mwa108
cm229
probable migraine with aura67
probable migraine with aura34
probable migraine with aura97
cm142
probable migraine with aura24
mwa55
mwa11
probable migraine with aura93
probable migraine with aura50
probable migraine with aura146
probable migraine with aura27
cm112
probable migraine with aura46
cm179
cm167
probable migraine with aura81
probable migraine with aura49
probable migraine with aura48
mwa40
probable migraine with aura102
mwa110
mwa92
probable migraine with aura63
probable migraine with aura134
probable migraine with aura122
mwa12
probable migraine with aura33
probable migraine with aura86
probable migraine with aura28
probable migraine with aura77
cm184
probable migraine with aura112

cluster_53
photophobia

cluster_47
no miosis

cluster_51
no rhinorrhea

cluster_45
no forehead and facial sweating

cluster_44
no eyelid edema

cluster_50
no ptosis

cluster_37
lacrimation

cluster_23
sudden
provoke by valsalva
provoke by cough
between 1 second to 2 hours

cluster_22
aggravated by physical activity

cluster_36
irregular frequency

cluster_20
2 to 30 minutes

cluster_34
max within 1 minute
severe
greater than 5 minutes

cluster_35
indomethacin responsive

cluster_21
4 to 72 hours

cluster_25
brought on by exercise
less than 48 hours

cluster_31
every other day to 8 per day

cluster_19
fixed in size and shape
1–6 cm in diameter
round or elliptical
sharply contoured

cluster_18
15 to 180 minutes

cluster_30
developing only during sleep and causing wakening

cluster_24
greater than 2 episodes
brought on by cold stimuli
resolve within 30 min after removal of cold

cluster_32
unilateral
eyelid edema

cluster_26
brought on within 1 hour of compression
resolve within 1 hour after removal of compression
maximal at site of compression

cluster_27
maximal at site of traction
brought on within 1 hour of traction
resolve within 1 hour after removal of traction

cluster_33
forehead and facial sweating

Parameter exploration for inflation = 1.5 and expansion = 9

cluster_0
migraine w/o aura17
probable migraine with aura21
Probable Infrequent Episodic Tension Type Headache42
Probable Chronic Tension Type Headache39
probable migraine with aura79
Probable Frequent Episodic Tension Type Headache28
mwa108
cm73
chronic tension type headache22
mwa99
infrequent tension type headache6
cm175
chronic tension type headache40
Probable Frequent Episodic Tension Type Headache34
cm214
Probable Infrequent Episodic Tension Type Headache49
Probable Chronic Tension Type Headache92
infrequent tension type headache12
Probable Chronic Tension Type Headache51
Probable Infrequent Episodic Tension Type Headache8
cm65

cm53
probable migraine without aura47
chronic tension type headache27
cm140
mwa82
mwa13
mwa68
hypnic9
Probable Chronic Tension Type Headache81
cm209
chronic tension type headache35
probable migraine with aura98
Probable Frequent Episodic Tension Type Headache64
mwa85
hc4
mwa41
cm172
mwa62
cm1
sun4
mwa4
cm136
probable migraine with aura5
Probable Infrequent Episodic Tension Type Headache20
cm207
Probable Frequent Episodic Tension Type Headache55
Probable Chronic Tension Type Headache16
mwa77
cm96
cm118
probable migraine with aura115
Probable Chronic Tension Type Headache102
Probable Chronic Tension Type Headache128
chronic tension type headache23
migraine w/o aura5
probable migraine without aura1
probable migraine without aura34
cm156
Probable Chronic Tension Type Headache89
probable migraine without aura36
probable migraine without aura32
Probable Cluster Headache5
mwa6
cm202
mwa29
Probable Frequent Episodic Tension Type Headache62
Probable Chronic Tension Type Headache48
cm7
mwa42
cm62

probable migraine with aura2
cm24
mwa20
Probable Chronic Tension Type Headache69
chronic tension type headache24
 migraine w/o aura20
cm107
probable migraine without aura39
mwa8
cm135
hypnic6
Probable Chronic Tension Type Headache91
Probable Chronic Tension Type Headache129
cm226
probable migraine without aura14
Probable Chronic Tension Type Headache120
probable migraine with aura31
probable migraine with aura143
Probable Frequent Episodic Tension Type Headache66
mwa32
Probable Hemicrania Continua8
mwa78
cm162
probable migraine with aura110
probable migraine with aura111
hypnic7
cm157
probable migraine with aura55
cm13
mwa73
probable migraine without aura26
probable migraine with aura57
probable migraine with aura61
Probable Infrequent Episodic Tension Type Headache12
Probable Frequent Episodic Tension Type Headache69
Probable Infrequent Episodic Tension Type Headache7
probable migraine without aura63
cm206
Probable Hemicrania Continua18
mwa53
cm240
Probable Chronic Tension Type Headache123
cm203
probable migraine without aura15
Probable Infrequent Episodic Tension Type Headache55
cm211
mwa109
cm182
cm213
frequent tension type headache13

probable migraine with aura140
Probable Frequent Episodic Tension Type Headache53
chronic tension type headache30
probable migraine with aura13
probable migraine with aura49
Probable Hemicrania Continua14
Probable Chronic Tension Type Headache117
cm92
cm254
Probable Frequent Episodic Tension Type Headache19
probable migraine with aura67
Probable Chronic Tension Type Headache119
cm152
stabbing2
probable migraine without aura45
stabbing1
cm122
Probable Cluster Headache8
cm271
Probable Chronic Tension Type Headache26
Probable Infrequent Episodic Tension Type Headache4
Probable Hemicrania Continua23
probable migraine with aura48
probable migraine with aura19
Probable Hemicrania Continua15
probable migraine without aura51
frequent tension type headache20
Probable Chronic Tension Type Headache43
Probable Chronic Tension Type Headache60
cm238
Probable Hemicrania Continua7
probable migraine with aura119
probable migraine without aura59
probable migraine without aura31
Probable Infrequent Episodic Tension Type Headache21
Probable Infrequent Episodic Tension Type Headache57
probable migraine with aura96
mwa88
cm11
Probable Chronic Tension Type Headache54
Probable Cluster Headache36
mwa2
cm45
probable migraine with aura144
probable migraine with aura62
cm144
Probable Chronic Tension Type Headache11
cm20
mwa94
infrequent tension type headache17

Probable Infrequent Episodic Tension Type Headache39
cm126
cm218
cm282
Probable Cluster Headache34
probable migraine with aura87
Probable Chronic Tension Type Headache13
frequent tension type headache8
probable migraine with aura103
frequent tension type headache3
sun6
Probable Frequent Episodic Tension Type Headache3
Probable Frequent Episodic Tension Type Headache16
cm40
Probable Frequent Episodic Tension Type Headache43
sun3
infrequent tension type headache15
probable migraine with aura59
probable migraine with aura82
Probable Hemicrania Continua1
migraine w/o aura2
Probable Chronic Tension Type Headache36
mwa47
probable migraine with aura56
Probable Hemicrania Continua2
mwa70
cm76
mwa34
cm37
cm167
mwa26
probable migraine without aura41
Probable Cluster Headache19
Probable Cluster Headache16
cm246
Probable Chronic Tension Type Headache31
Probable Chronic Tension Type Headache70
Probable Frequent Episodic Tension Type Headache59
probable migraine with aura113
cm194
chronic tension type headache15
exercise1
Probable Infrequent Episodic Tension Type Headache28
cm66
probable migraine without aura20
mwa55
Probable Chronic Tension Type Headache3
mwa35
infrequent tension type headache19
probable migraine with aura43

chronic tension type headache38
cm54
mwa86
cm183
probable migraine with aura137
cm128
Probable Frequent Episodic Tension Type Headache5
probable migraine with aura83
cm146
Probable Frequent Episodic Tension Type Headache9
Probable Chronic Tension Type Headache79
cm115
cm187
probable migraine with aura99
Probable Infrequent Episodic Tension Type Headache68
Probable Cluster Headache37
chronic tension type headache13
probable migraine with aura80
sun7
mwa28
Probable Chronic Tension Type Headache121
mwa76
stabbing8
infrequent tension type headache22
cm200
ph5
cm169
Probable Infrequent Episodic Tension Type Headache11
cm68
Probable Frequent Episodic Tension Type Headache29
Probable Chronic Tension Type Headache23
mwa38
mwa67
mwa96
cm99
Probable Chronic Tension Type Headache63
Probable Cluster Headache7
chronic tension type headache37
Probable Frequent Episodic Tension Type Headache60
Probable Infrequent Episodic Tension Type Headache38
Probable Chronic Tension Type Headache101
Probable Cluster Headache17
Probable Chronic Tension Type Headache130
cm90
cm39
probable migraine without aura75
Probable Infrequent Episodic Tension Type Headache14
Probable Chronic Tension Type Headache100
cm80
probable migraine without aura8

probable migraine with aura131
probable migraine with aura64
cluster7
Probable Frequent Episodic Tension Type Headache57
cm171
ph2
probable migraine with aura9
probable migraine with aura128
probable migraine with aura129
cm112
mwa103
mwa84
infrequent tension type headache4
cm185
Probable Infrequent Episodic Tension Type Headache30
Probable Cluster Headache21
mwa14
Probable Chronic Tension Type Headache77
Probable Hemicrania Continua4
probable migraine without aura69
Probable Infrequent Episodic Tension Type Headache64
mwa60
cm278
Probable Cluster Headache2
frequent tension type headache21
probable migraine with aura45
Probable Chronic Tension Type Headache5
Probable Frequent Episodic Tension Type Headache26
frequent tension type headache5
probable migraine with aura24
probable migraine without aura30
probable migraine with aura30
Probable Frequent Episodic Tension Type Headache7
Probable Frequent Episodic Tension Type Headache33
Probable Chronic Tension Type Headache1
chronic tension type headache21
Probable Frequent Episodic Tension Type Headache38
Probable Frequent Episodic Tension Type Headache36
Probable Frequent Episodic Tension Type Headache70
cm268
Probable Chronic Tension Type Headache94
probable migraine with aura53
mwa71
cm264
cm110
probable migraine with aura97
probable migraine with aura142
cm252
Probable Chronic Tension Type Headache74
cm55

mwa16
probable migraine with aura122
Probable Frequent Episodic Tension Type Headache10
cm199
Probable Chronic Tension Type Headache103
 migraine w/o aura14
chronic tension type headache9
cm179
Probable Chronic Tension Type Headache104
cm56
chronic tension type headache25
chronic tension type headache29
Probable Chronic Tension Type Headache114
compression1
cm257
mwa107
cm262
cm253
probable migraine with aura106
infrequent tension type headache3
hc8
probable migraine with aura125
probable migraine without aura17
Probable Frequent Episodic Tension Type Headache41
cm6
Probable Infrequent Episodic Tension Type Headache24
Probable Infrequent Episodic Tension Type Headache17
Probable Hemicrania Continua10
cm247
ph7
probable migraine with aura117
probable migraine with aura124
frequent tension type headache11
chronic tension type headache32
probable migraine with aura146
Probable Chronic Tension Type Headache15
mwa25
frequent tension type headache12
cm191
probable migraine without aura71
cm105
probable migraine with aura90
Probable Chronic Tension Type Headache41
probable migraine without aura66
cm25
Probable Chronic Tension Type Headache53
probable migraine without aura61
Probable Hemicrania Continua17
probable migraine with aura77
probable migraine with aura38

hypnic5
cm145
cm81
hc1
infrequent tension type headache2
probable migraine with aura101
cm260
probable migraine without aura9
Probable Chronic Tension Type Headache40
probable migraine with aura66
Probable Chronic Tension Type Headache67
Probable Chronic Tension Type Headache75
Probable Chronic Tension Type Headache132
probable migraine without aura24
cm36
cm154
probable migraine with aura37
Probable Cluster Headache30
cm60
chronic tension type headache43
cm163
cm208
cm186
cm119
cm277
Probable Frequent Episodic Tension Type Headache71
cm47
Probable Chronic Tension Type Headache72
cm279
sun1
mwa64
cm274
mwa120
probable migraine with aura93
Probable Infrequent Episodic Tension Type Headache70
mwa79
cm212
probable migraine with aura95
cm63
chronic tension type headache42
Probable Chronic Tension Type Headache73
Probable Chronic Tension Type Headache88
chronic tension type headache5
mwa81
cm283
Probable Frequent Episodic Tension Type Headache15
cm265
Probable Hemicrania Continua16
Probable Infrequent Episodic Tension Type Headache33
cm231

migraine w/o aura10
chronic tension type headache11
probable migraine without aura48
mwa1
Probable Hemicrania Continua6
cm29
Probable Chronic Tension Type Headache93
hc7
probable migraine with aura41
stabbing4
cm255
cough2
mwa24
cm129
ph3
probable migraine with aura100
Probable Infrequent Episodic Tension Type Headache22
mwa113
cm51
infrequent tension type headache9
Probable Infrequent Episodic Tension Type Headache47
Probable Chronic Tension Type Headache10
Probable Chronic Tension Type Headache96
Probable Cluster Headache15
probable migraine with aura29
probable migraine with aura51
cm109
Probable Chronic Tension Type Headache98
Probable Infrequent Episodic Tension Type Headache77
Probable Chronic Tension Type Headache49
Probable Frequent Episodic Tension Type Headache39
cm19
cm26
cm86
Probable Infrequent Episodic Tension Type Headache35
cm228
Probable Chronic Tension Type Headache21
Probable Chronic Tension Type Headache113
Probable Infrequent Episodic Tension Type Headache34
cm215
probable migraine without aura70
Probable Cluster Headache26
mwa91
probable migraine with aura74
probable migraine with aura40
probable migraine with aura65
Probable Chronic Tension Type Headache6
mwa49
chronic tension type headache31
Probable Hemicrania Continua9

infrequent tension type headache11
Probable Cluster Headache1
cm83
cm150
 migraine w/o aura9
cm259
Probable Infrequent Episodic Tension Type Headache52
Probable Frequent Episodic Tension Type Headache49
probable migraine without aura44
hypnic2
ph4
mwa33
chronic tension type headache41
Probable Frequent Episodic Tension Type Headache14
cm177
probable migraine with aura54
cm116
hypnic3
mwa21
probable migraine without aura55
probable migraine without aura67
probable migraine without aura43
Probable Infrequent Episodic Tension Type Headache65
Probable Hemicrania Continua11
cm42
infrequent tension type headache8
Probable Chronic Tension Type Headache7
cm181
Probable Frequent Episodic Tension Type Headache76
Probable Infrequent Episodic Tension Type Headache25
hc2
mwa114
cm121
cm141
probable migraine without aura4
probable migraine without aura12
mwa17
mwa27
mwa18
Probable Cluster Headache35
Probable Chronic Tension Type Headache19
cm155
cm159
mwa74
cm3
Probable Frequent Episodic Tension Type Headache1
Probable Frequent Episodic Tension Type Headache13
cm204
Probable Frequent Episodic Tension Type Headache47
Probable Infrequent Episodic Tension Type Headache50

Probable Frequent Episodic Tension Type Headache11
migraine w/o aura19
ph6
cm250
Probable Frequent Episodic Tension Type Headache48
cm241
Probable Infrequent Episodic Tension Type Headache15
chronic tension type headache3
cm176
cm124
mwa19
Probable Frequent Episodic Tension Type Headache31
cluster5
cm148
cm188
Probable Frequent Episodic Tension Type Headache21
cm77
probable migraine without aura62
Probable Frequent Episodic Tension Type Headache30
cm69
probable migraine without aura76
cm30
Probable Frequent Episodic Tension Type Headache37
Probable Chronic Tension Type Headache55
Probable Cluster Headache28
hc9
Probable Chronic Tension Type Headache107
mwa80
cm114
ph1
thunderclap1
frequent tension type headache1
frequent tension type headache15
probable migraine without aura50
Probable Cluster Headache3
probable migraine without aura52
probable migraine with aura134
cm93
probable migraine with aura126
Probable Chronic Tension Type Headache110
probable migraine without aura46
cm210
mwa97
mwa116
mwa48
Probable Hemicrania Continua20
Probable Hemicrania Continua5
Probable Frequent Episodic Tension Type Headache40
probable migraine with aura89
cm44

mwa100
probable migraine with aura92
Probable Chronic Tension Type Headache112
Probable Hemicrania Continua25
Probable Infrequent Episodic Tension Type Headache67
cm160
cm74
mwa95
cm225
cm85
Probable Frequent Episodic Tension Type Headache51
Probable Cluster Headache4
probable migraine with aura58
Probable Infrequent Episodic Tension Type Headache18
Probable Cluster Headache33
probable migraine with aura44
Probable Chronic Tension Type Headache87
mwa65
cm153
Probable Infrequent Episodic Tension Type Headache36
probable migraine without aura6
Probable Chronic Tension Type Headache35
cm276
Probable Infrequent Episodic Tension Type Headache16
Probable Chronic Tension Type Headache50
mwa115
probable migraine with aura20
probable migraine without aura19
Probable Infrequent Episodic Tension Type Headache10
cm235
probable migraine with aura50
frequent tension type headache14
Probable Infrequent Episodic Tension Type Headache23
Probable Infrequent Episodic Tension Type Headache53
probable migraine with aura123
cm158
Probable Frequent Episodic Tension Type Headache6
mwa22
probable migraine with aura127
Probable Frequent Episodic Tension Type Headache61
Probable Frequent Episodic Tension Type Headache68
Probable Cluster Headache9
cm57
probable migraine with aura16
Probable Chronic Tension Type Headache122
frequent tension type headache19
cm21
Probable Infrequent Episodic Tension Type Headache66
Probable Chronic Tension Type Headache29
cm88

probable migraine with aura28
Probable Chronic Tension Type Headache90
cm75
hc6
chronic tension type headache2
probable migraine with aura118
stabbing5
mwa111
cm196
cm10
cm189
Probable Hemicrania Continua27
probable migraine without aura42
probable migraine with aura108
cm16
cm216
probable migraine without aura16
probable migraine with aura94
Probable Frequent Episodic Tension Type Headache35
probable migraine with aura112
probable migraine without aura13
probable migraine with aura139
Probable Chronic Tension Type Headache58
mwa87
chronic tension type headache36
Probable Chronic Tension Type Headache86
Probable Infrequent Episodic Tension Type Headache44
Probable Chronic Tension Type Headache106
Probable Cluster Headache13
chronic tension type headache19
Probable Cluster Headache10
cm14
Probable Chronic Tension Type Headache37
cm117
mwa57
mwa72
probable migraine with aura81
cm12
Probable Chronic Tension Type Headache42
cough1
cm165
probable migraine with aura138
probable migraine with aura39
Probable Chronic Tension Type Headache24
cm120
Probable Cluster Headache27
Probable Infrequent Episodic Tension Type Headache26
mwa54
Probable Infrequent Episodic Tension Type Headache75
probable migraine with aura104

probable migraine with aura120
mwa9
cm168
cm178
hypnic1
cm234
probable migraine without aura73
cm84
chronic tension type headache14
 migraine w/o aura22
probable migraine with aura133
cm192
sun8
Probable Infrequent Episodic Tension Type Headache29
Probable Frequent Episodic Tension Type Headache65
probable migraine without aura11
Probable Chronic Tension Type Headache71
Probable Chronic Tension Type Headache99
cm95
cm71
cm233
cm97
mwa3
mwa106
probable migraine without aura28
Probable Chronic Tension Type Headache44
cm2
cm275
cm9
cm205
mwa39
Probable Frequent Episodic Tension Type Headache63
probable migraine with aura68
probable migraine with aura70
Probable Infrequent Episodic Tension Type Headache9
chronic tension type headache17
Probable Infrequent Episodic Tension Type Headache74
probable migraine with aura1
probable migraine with aura11
Probable Chronic Tension Type Headache17
probable migraine with aura145
cm195
Probable Cluster Headache22
cluster3
cm70
cm134
Probable Frequent Episodic Tension Type Headache20
Probable Cluster Headache11
mwa69
probable migraine with aura35

cm173
 migraine w/o aura16
probable migraine with aura85
Probable Chronic Tension Type Headache9
Probable Hemicrania Continua22
cm8
Probable Chronic Tension Type Headache108
frequent tension type headache22
cm104
cm94
cm125
cm198
cm239
chronic tension type headache10
mwa31
probable migraine without aura58
Probable Infrequent Episodic Tension Type Headache45
Probable Frequent Episodic Tension Type Headache2
 migraine w/o aura1
mwa37
probable migraine without aura33
probable migraine with aura84
Probable Frequent Episodic Tension Type Headache24
ndph1
Probable Infrequent Episodic Tension Type Headache62
Probable Chronic Tension Type Headache111
cm103
cm170
Probable Chronic Tension Type Headache115
Probable Infrequent Episodic Tension Type Headache63
Probable Chronic Tension Type Headache30
 migraine w/o aura4
frequent tension type headache10
hypnic4
 migraine w/o aura18
Probable Chronic Tension Type Headache2
cm197
coldHA1
Probable Frequent Episodic Tension Type Headache52
probable migraine with aura130
probable migraine with aura52
mwa52
mwa58
mwa110
cm34
Probable Chronic Tension Type Headache47
Probable Frequent Episodic Tension Type Headache44
cm270
mwa102
mwa51

cm269
probable migraine without aura54
cm263
infrequent tension type headache16
chronic tension type headache28
infrequent tension type headache7
Probable Chronic Tension Type Headache127
mwa83
Probable Chronic Tension Type Headache65
Probable Chronic Tension Type Headache25
Probable Frequent Episodic Tension Type Headache22
cm166
infrequent tension type headache10
mwa63
probable migraine without aura35
Probable Chronic Tension Type Headache126
cm161
Probable Chronic Tension Type Headache4
cm23
probable migraine without aura5
sun5
probable migraine with aura76
probable migraine with aura47
cm31
Probable Frequent Episodic Tension Type Headache72
Probable Infrequent Episodic Tension Type Headache69
probable migraine with aura15
probable migraine without aura68
Probable Chronic Tension Type Headache52
cm245
chronic tension type headache16
Probable Frequent Episodic Tension Type Headache23
cm4
Probable Infrequent Episodic Tension Type Headache48
Probable Frequent Episodic Tension Type Headache73
mwa23
cm22
cm256
probable migraine without aura10
stabbing6
probable migraine with aura114
Probable Infrequent Episodic Tension Type Headache56
cm61
Probable Chronic Tension Type Headache59
Probable Cluster Headache25
probable migraine with aura132
sex2
probable migraine without aura64
probable migraine with aura135
cm130

Probable Frequent Episodic Tension Type Headache45
Probable Hemicrania Continua28
probable migraine with aura22
stabbing3
mwa92
cm5
cm219
cm267
cm89
mwa117
frequent tension type headache2
cm242
probable migraine with aura88
Probable Infrequent Episodic Tension Type Headache3
Probable Frequent Episodic Tension Type Headache17
cm72
cm137
cm284
probable migraine without aura53
probable migraine with aura71
Probable Chronic Tension Type Headache57
Probable Chronic Tension Type Headache105
Probable Hemicrania Continua13
Probable Hemicrania Continua3
infrequent tension type headache1
probable migraine with aura3
frequent tension type headache7
sex3
migraine w/o aura3
migraine w/o aura11
cluster2
mwa75
Probable Hemicrania Continua24
Probable Infrequent Episodic Tension Type Headache6
Probable Chronic Tension Type Headache124
Probable Frequent Episodic Tension Type Headache27
cm243
cm27
infrequent tension type headache21
cm123
Probable Infrequent Episodic Tension Type Headache41
chronic tension type headache6
Probable Frequent Episodic Tension Type Headache77
cm46
probable migraine without aura25
Probable Frequent Episodic Tension Type Headache74
mwa104
infrequent tension type headache5
cm102
cluster6

probable migraine without aura60
mwa43
cm266
mwa36
frequent tension type headache16
cm101
mwa45
cm131
Probable Frequent Episodic Tension Type Headache54
probable migraine with aura78
Probable Chronic Tension Type Headache28
Probable Infrequent Episodic Tension Type Headache31
probable migraine without aura3
probable migraine with aura6
Probable Chronic Tension Type Headache68
cm220
cm273
probable migraine without aura27
Probable Frequent Episodic Tension Type Headache25
cm281
cm222
cm164
probable migraine without aura40
probable migraine without aura57
migraine w/o aura6
cm223
Probable Infrequent Episodic Tension Type Headache71
cm217
cm201
cm190
chronic tension type headache4
cm151
probable migraine with aura116
mwa119
probable migraine without aura22
Probable Chronic Tension Type Headache45
cm50
cm132
cm127
chronic tension type headache7
chronic tension type headache34
Probable Chronic Tension Type Headache85
migraine w/o aura13
cm87
cm106
cm143
probable migraine with aura63
Probable Chronic Tension Type Headache83
cm113
cm272

cm98
Probable Chronic Tension Type Headache131
mwa61
probable migraine with aura8
Probable Frequent Episodic Tension Type Headache56
Probable Chronic Tension Type Headache116
Probable Infrequent Episodic Tension Type Headache43
cm180
cm78
probable migraine without aura49
Probable Chronic Tension Type Headache61
probable migraine without aura65
probable migraine without aura23
mwa93
cm193
probable migraine with aura12
probable migraine with aura141
cm138
cm147
sex1
probable migraine with aura34
Probable Chronic Tension Type Headache34
cm244
Probable Chronic Tension Type Headache32
cm28
cm67
Probable Frequent Episodic Tension Type Headache58
mwa101
cm33
probable migraine without aura56
ph8
cm251
probable migraine with aura136
cm108
probable migraine with aura23
Probable Infrequent Episodic Tension Type Headache58
Probable Chronic Tension Type Headache18
probable migraine with aura36
Probable Frequent Episodic Tension Type Headache8
Probable Chronic Tension Type Headache22
sun2
mwa66
cm149
Probable Cluster Headache20
Probable Cluster Headache32
chronic tension type headache12
Probable Hemicrania Continua26
Probable Chronic Tension Type Headache125
Probable Frequent Episodic Tension Type Headache18
cm248

hypnic8
mwa44
Probable Frequent Episodic Tension Type Headache12
cm15
 migraine w/o aura21
Probable Chronic Tension Type Headache8
probable migraine with aura60
Probable Frequent Episodic Tension Type Headache42
chronic tension type headache18
mwa7
cluster1
cm230
probable migraine with aura32
mwa56
Probable Frequent Episodic Tension Type Headache32
mwa118
Probable Cluster Headache24
mwa46
infrequent tension type headache13
Probable Frequent Episodic Tension Type Headache4
mwa112
cm139
Probable Infrequent Episodic Tension Type Headache54
Probable Chronic Tension Type Headache62
cm17
mwa105
cluster9
sex4
Probable Infrequent Episodic Tension Type Headache27
Probable Infrequent Episodic Tension Type Headache59
cm43
Probable Infrequent Episodic Tension Type Headache76
probable migraine with aura75
Probable Infrequent Episodic Tension Type Headache19
Probable Cluster Headache29
probable migraine with aura46
frequent tension type headache4
 migraine w/o aura8
Probable Infrequent Episodic Tension Type Headache60
chronic tension type headache44
Probable Chronic Tension Type Headache80
cm184
probable migraine without aura74
cm35
Probable Chronic Tension Type Headache27
Probable Chronic Tension Type Headache76
cm79
mwa10
chronic tension type headache1
hc5

cluster4
cm18
cm232
cm58
cm258
frequent tension type headache6
cm133
probable migraine without aura37
Probable Chronic Tension Type Headache109
Probable Infrequent Episodic Tension Type Headache5
cm261
Probable Infrequent Episodic Tension Type Headache2
frequent tension type headache17
Probable Infrequent Episodic Tension Type Headache37
Probable Frequent Episodic Tension Type Headache50
probable migraine without aura21
frequent tension type headache18
mwa40
Probable Frequent Episodic Tension Type Headache75
Probable Chronic Tension Type Headache46
cm59
cm237
Probable Cluster Headache6
Probable Infrequent Episodic Tension Type Headache40
stabbing7
mwa59
probable migraine with aura25
chronic tension type headache33
cm221
cm111
Probable Chronic Tension Type Headache33
Probable Hemicrania Continua12
chronic tension type headache20
probable migraine with aura27
cm229
probable migraine with aura7
traction1
Probable Infrequent Episodic Tension Type Headache46
probable migraine without aura29
probable migraine with aura121
mwa98
Probable Infrequent Episodic Tension Type Headache61
frequent tension type headache9
cm174
probable migraine without aura38
chronic tension type headache39
Probable Cluster Headache23
probable migraine with aura4
mwa11
mwa15

migraine w/o aura15
Probable Chronic Tension Type Headache12
Probable Infrequent Episodic Tension Type Headache72
Probable Hemicrania Continua21
cm49
Probable Infrequent Episodic Tension Type Headache51
Probable Cluster Headache18
cm142
probable migraine with aura18
Probable Chronic Tension Type Headache95
Probable Frequent Episodic Tension Type Headache46
cm38
cm41
probable migraine with aura14
Probable Infrequent Episodic Tension Type Headache32
probable migraine without aura7
cm82
Probable Chronic Tension Type Headache82
mwa50
cm91
probable migraine with aura109
mwa5
probable migraine without aura2
probable migraine with aura105
chronic tension type headache26
Probable Frequent Episodic Tension Type Headache67
Probable Chronic Tension Type Headache64
ph9
mwa30
infrequent tension type headache18
cluster8
probable migraine with aura72
Probable Chronic Tension Type Headache56
Probable Chronic Tension Type Headache66
probable migraine without aura18
cm227
probable migraine with aura33
cm32
migraine w/o aura7
cm224
probable migraine without aura72
probable migraine with aura69
Probable Chronic Tension Type Headache84
Probable Chronic Tension Type Headache14
Probable Chronic Tension Type Headache118
probable migraine with aura102
cm280
cm48
Probable Chronic Tension Type Headache97
cm52

Probable Chronic Tension Type Headache78
mwa12
Probable Cluster Headache14
mwa90
probable migraine with aura42
probable migraine with aura86
cm100
Probable Chronic Tension Type Headache38
infrequent tension type headache14
probable migraine with aura26
mwa89
cm64
probable migraine with aura17
Probable Cluster Headache12
probable migraine with aura107
Probable Hemicrania Continua19
probable migraine with aura10
cm236
chronic tension type headache8
Probable Infrequent Episodic Tension Type Headache13
Probable Infrequent Episodic Tension Type Headache1
migraine w/o aura12
probable migraine with aura91
Probable Infrequent Episodic Tension Type Headache73
probable migraine without aura77
Probable Cluster Headache31
cm249
Probable Chronic Tension Type Headache20
probable migraine with aura73
infrequent tension type headache20
hc3
aggravated by physical activity
at least one aura symptom is unilateral
photophobia
greater than 8 days per month
miosis
unremitting within 24 hours
retinal aura
lacrimation
conjunctival injection
unilateral
pulsating
orbital or supraorbital or temporal pain
sensory aura
constant
brought on within 1 hour of compression
15 to 180 minutes
brought on by cold stimuli
nasal congestion
1 minute to 72 hours with severe

developing only during sleep and causing wakening
indomethacin responsive
no forehead and facial sweating
provoke by valsalva
up to few seconds
nonpulsating
maximal at site of compression
up to 72 hours with mild
at least one aura symptom spreads gradually over 5 minutes
less than 48 hours
less than 12 days per year
30 min to 7 days in duration
brought on within 1 hour of traction
greater than 5 minutes
no nasal congestion
no photophobia
1 to 14 days per month
no ptosis
no restless
two or more aura symptoms occur in succession
greater than 5 episodes
bilateral location
ptosis
no rhinorrhea
eyelid edema
resolve within 30 min after removal of cold
more than 10 episodes
4 to 72 hours
speech and/or language aura
between 1 second to 2 hours
brought on by exercise
no conjunctival injection
fully reversible
phonophobia
hours to days
severe
no nausea/vomiting
resolve within 1 hour after removal of traction
greater than 2 episodes
max within 1 minute
nausea/vomiting
15 minutes up to four hours after waking
relieve by triptan or ergot
restless
each individual aura symptom lasts 5–60 minutes
irregular frequency
rhinorrhea
the aura is accompanied, or followed within 60 minutes, by headache
more than 3 months
no eyelid edema

brainstem aura
clearly remembered onset
greater than 20 episodes
no phonophobia
maximal at site of traction
forehead and facial sweating
moderate to severe
greater than 15 days per month
single or series of stabs
sudden
abrupt explosive intensity just before or with orgasm
at least one aura symptom is positive
every other day to 8 per day
1 to 600 seconds
more than 10 days per month
greater than 1 per day
not aggrevated by activity
resolve within 1 hour after removal of compression
increasing in intensity with increasing sexual excitement
no miosis
brought on by sex
mild to moderate pain
greater than 5 per day
visual aura
no lacrimation
motor aura
2 to 30 minutes
provoke by cough

cluster_1
nummular1
sharply contoured
1–6 cm in diameter
fixed in size and shape
round or elliptical

cluster_3
no orbital or supraorbital or temporal pain

cluster_2
more than 1 episode per day

Parameter explorations for inflation = 3.3 and expansion = 2

cluster_210
visual aura

cluster_204
sensory aura

cluster_199
pulsating

cluster_16
Probable Cluster Headache28
cluster9
Probable Cluster Headache25

cluster_166
max within 1 minute
severe
orbital or supraorbital or temporal pain
greater than 5 minutes

cluster_172
lacrimation

cluster_173
more than 10 episodes
less than 12 days per year

cluster_167
greater than 5 per day

cluster_17
Probable Cluster Headache10
ph1
Probable Cluster Headache13
Probable Cluster Headache11

cluster_198
ptosis

cluster_205
speech and/or language aura

cluster_207
two or more aura symptoms occur in succession

cluster_29
sun4
Probable Hemicrania Continua17
Probable Hemicrania Continua16

cluster_15
Probable Cluster Headache27
cluster8

Probable Cluster Headache24

cluster_159
conjunctival injection

cluster_171
single or series of stabs
irregular frequency
up to few seconds

cluster_165
greater than 20 episodes

cluster_164
greater than 1 per day

cluster_170
indomethacin responsive

cluster_158
clearly remembered onset
no nausea/vomiting
constant
unremitting within 24 hours

cluster_14
Probable Cluster Headache26
cluster7
Probable Cluster Headache23

cluster_28
Probable Hemicrania Continua14
Probable Hemicrania Continua13
sun3

cluster_206
the aura is accompanied, or followed within 60 minutes, by headache

cluster_202
retinal aura

cluster_10
Probable Cluster Headache16
cluster3
Probable Cluster Headache12

cluster_38
Probable Hemicrania Continua5
Probable Cluster Headache5
hc5

Probable Hemicrania Continua24
Probable Hemicrania Continua14

cluster_174
mild to moderate pain

cluster_160
each individual aura symptom lasts 5–60 minutes

cluster_148
at least one aura symptom is unilateral

cluster_149
at least one aura symptom spreads gradually over 5 minutes

cluster_161
every other day to 8 per day

cluster_175
miosis

cluster_39
Probable Chronic Tension Type Headache23
Probable Hemicrania Continua22
Probable Chronic Tension Type Headache1
Probable Chronic Tension Type Headache15
Probable Hemicrania Continua25
Probable Chronic Tension Type Headache41
Probable Chronic Tension Type Headache2
Probable Chronic Tension Type Headache69
Probable Chronic Tension Type Headache95
Probable Chronic Tension Type Headache16
Probable Chronic Tension Type Headache3
Probable Cluster Headache6
Probable Hemicrania Continua6
hc6
Probable Chronic Tension Type Headache45
Probable Chronic Tension Type Headache42
Probable Hemicrania Continua16

cluster_11
cluster4
Probable Cluster Headache20
Probable Cluster Headache17

cluster_203
rhinorrhea

cluster_201
restless

cluster_13
cluster6
Probable Cluster Headache22
Probable Cluster Headache19

cluster_188
no phonophobia

cluster_163
forehead and facial sweating

cluster_177
more than 1 episode per day

cluster_176
moderate to severe

cluster_162
eyelid edema

cluster_189
no photophobia

cluster_12
Probable Cluster Headache21
cluster5
Probable Cluster Headache18

cluster_200
relieve by triptan or ergot

cluster_49
cm53
cm35
cm56
cm54
cm190
cm95
cm132
cm137
cm138
cm189
cm193
cm34
cm133
cm234
cm19
cm170
cm16

cm169
cm174
cm172
cm58
cm17
cm36
cm188
cm191
cm40
cm18
cm55
cm233
cm141
cm102
cm37
cm228
cm39
cm192
cm175
cm57
cm171
cm101
cm262

cluster_75
hypnic8

cluster_61
stabbing3

cluster_139
developing only during sleep and causing wakening
greater than 15 days per month
more than 3 months
more than 10 days per month
15 minutes up to four hours after waking

cluster_105
probable migraine with aura22

cluster_111
Probable Frequent Episodic Tension Type Headache46
Probable Infrequent Episodic Tension Type Headache18
Probable Infrequent Episodic Tension Type Headache31
Probable Frequent Episodic Tension Type Headache14
Probable Frequent Episodic Tension Type Headache59
Probable Frequent Episodic Tension Type Headache25
Probable Frequent Episodic Tension Type Headache44
Probable Infrequent Episodic Tension Type Headache7
Probable Frequent Episodic Tension Type Headache75

Probable Frequent Episodic Tension Type Headache10
Probable Infrequent Episodic Tension Type Headache5
Probable Infrequent Episodic Tension Type Headache20
Probable Infrequent Episodic Tension Type Headache61
Probable Infrequent Episodic Tension Type Headache16
Probable Frequent Episodic Tension Type Headache23
Probable Infrequent Episodic Tension Type Headache9
Probable Infrequent Episodic Tension Type Headache27
Probable Infrequent Episodic Tension Type Headache29
Probable Frequent Episodic Tension Type Headache21
Probable Infrequent Episodic Tension Type Headache40
Probable Frequent Episodic Tension Type Headache12
Probable Frequent Episodic Tension Type Headache50

cluster_6
compression1

cluster_7
traction1

cluster_110
Probable Infrequent Episodic Tension Type Headache17
Probable Infrequent Episodic Tension Type Headache15
Probable Infrequent Episodic Tension Type Headache4
Probable Frequent Episodic Tension Type Headache58
Probable Frequent Episodic Tension Type Headache22
Probable Frequent Episodic Tension Type Headache20
Probable Frequent Episodic Tension Type Headache45
Probable Infrequent Episodic Tension Type Headache28
Probable Frequent Episodic Tension Type Headache24
Probable Infrequent Episodic Tension Type Headache26
Probable Infrequent Episodic Tension Type Headache30
Probable Frequent Episodic Tension Type Headache74
Probable Infrequent Episodic Tension Type Headache6
Probable Frequent Episodic Tension Type Headache49
Probable Infrequent Episodic Tension Type Headache60
Probable Frequent Episodic Tension Type Headache13
Probable Frequent Episodic Tension Type Headache11
Probable Infrequent Episodic Tension Type Headache19
Probable Infrequent Episodic Tension Type Headache39
Probable Frequent Episodic Tension Type Headache43
Probable Frequent Episodic Tension Type Headache9
Probable Infrequent Episodic Tension Type Headache8

cluster_104
probable migraine with aura21

cluster_138
1 to 600 seconds

cluster_60
stabbing2

cluster_74
hypnic7

cluster_48
probable migraine with aura92
mwa9
probable migraine with aura85
probable migraine with aura91
probable migraine with aura146
probable migraine with aura86
mwa112
mwa91
probable migraine with aura55
probable migraine with aura122
mwa59
mwa65
mwa60
probable migraine with aura117
mwa120
probable migraine with aura119
probable migraine with aura138
mwa30
probable migraine with aura7
probable migraine with aura141
mwa66
mwa27
mwa96
probable migraine with aura125
probable migraine with aura35
probable migraine with aura88
probable migraine with aura90
mwa29
probable migraine with aura140
mwa62
mwa114
probable migraine with aura124
mwa93
mwa98
mwa115
probable migraine with aura53
mwa97
mwa64
probable migraine with aura123
probable migraine with aura56
mwa99

cluster_62

stabbing4

cluster_76
hypnic9

cluster_89
probable migraine without aura13

cluster_112
Probable Frequent Episodic Tension Type Headache3
Probable Frequent Episodic Tension Type Headache28
Probable Frequent Episodic Tension Type Headache2
Probable Frequent Episodic Tension Type Headache5
Probable Frequent Episodic Tension Type Headache4
Probable Frequent Episodic Tension Type Headache8
Probable Frequent Episodic Tension Type Headache1
Probable Frequent Episodic Tension Type Headache53
Probable Frequent Episodic Tension Type Headache16
Probable Frequent Episodic Tension Type Headache15
Probable Frequent Episodic Tension Type Headache19

cluster_106
probable migraine with aura27

cluster_5
coldHA1

cluster_4
thunderclap1

cluster_107
probable migraine with aura28

cluster_113
Probable Chronic Tension Type Headache102
Probable Chronic Tension Type Headache4
Probable Chronic Tension Type Headache25
Probable Chronic Tension Type Headache5
Probable Chronic Tension Type Headache78
Probable Chronic Tension Type Headache46
Probable Chronic Tension Type Headache24
Probable Chronic Tension Type Headache28
Probable Chronic Tension Type Headache50
Probable Chronic Tension Type Headache6
Probable Chronic Tension Type Headache47

cluster_88
probable migraine without aura19
probable migraine without aura44
probable migraine without aura11

cluster_77
cough1

cluster_63
stabbing5

cluster_67
nummular1

cluster_73
hypnic6

cluster_117
Probable Cluster Headache1

cluster_103
probable migraine with aura20

cluster_98
probable migraine with aura15

cluster_0
probable migraine without aura35
 migraine w/o aura3
probable migraine without aura42
probable migraine without aura23
cm13
probable migraine without aura25
 migraine w/o aura17
cm92
cm12
 migraine w/o aura8
cm11
cm2
probable migraine without aura33
cm84
probable migraine without aura17
probable migraine without aura21
cm3
probable migraine without aura27
 migraine w/o aura6
probable migraine without aura1
cm4
cm93
cm5
cm6
cm8
cm153
 migraine w/o aura7

cm1
probable migraine without aura69
 migraine w/o aura13
probable migraine without aura20
probable migraine without aura55
 migraine w/o aura4
 migraine w/o aura5
probable migraine without aura45
probable migraine without aura7
cm20
probable migraine without aura63
cm109
probable migraine without aura3
 migraine w/o aura10
probable migraine without aura22
probable migraine without aura41
probable migraine without aura14
probable migraine without aura50
 migraine w/o aura1
probable migraine without aura12
 migraine w/o aura2
probable migraine without aura9
probable migraine without aura10
cm7
probable migraine without aura39
cm226
probable migraine without aura18
probable migraine without aura5
cm28
probable migraine without aura24
probable migraine without aura38
cm21
probable migraine without aura31
probable migraine without aura48
cm23
probable migraine without aura29
probable migraine without aura16
cm9
probable migraine without aura8

cluster_1
 migraine w/o aura22
probable migraine without aura40
 migraine w/o aura16
probable migraine without aura30
cm24
 migraine w/o aura14
cm31
probable migraine without aura75
probable migraine without aura49

probable migraine without aura53
probable migraine without aura61
cm235
cm114
cm94
cm103
probable migraine without aura34
cm15
cm157
migraine w/o aura18
migraine w/o aura21
probable migraine without aura46
probable migraine without aura47
cm236
probable migraine without aura64
migraine w/o aura11
cm259
cm25
migraine w/o aura12
probable migraine without aura74
probable migraine without aura51
probable migraine without aura65
probable migraine without aura70
probable migraine without aura52
probable migraine without aura58
probable migraine without aura43
cm128
probable migraine without aura77
cm10
migraine w/o aura15
probable migraine without aura36
probable migraine without aura72
cm166
cm156
probable migraine without aura62
probable migraine without aura57
probable migraine without aura56
probable migraine without aura68
cm279
cm227
cm97
cm155
probable migraine without aura59
probable migraine without aura76
cm14
cm130
migraine w/o aura9
probable migraine without aura54
cm284
migraine w/o aura19

migraine w/o aura20
probable migraine without aura73
probable migraine without aura67
probable migraine without aura15
probable migraine without aura37
probable migraine without aura66
probable migraine without aura71

cluster_99
probable migraine with aura16

cluster_102
probable migraine with aura19

cluster_116
Probable Cluster Headache19

cluster_72
hypnic5

cluster_66
stabbing8

cluster_70
hypnic3

cluster_64
stabbing6

cluster_58
sex4

cluster_100
probable migraine with aura17

cluster_114
Probable Chronic Tension Type Headache57
Probable Chronic Tension Type Headache111
Probable Chronic Tension Type Headache61
Probable Chronic Tension Type Headache113
Probable Chronic Tension Type Headache53
Probable Chronic Tension Type Headache83
Probable Chronic Tension Type Headache129
Probable Chronic Tension Type Headache89
Probable Chronic Tension Type Headache87
Probable Chronic Tension Type Headache131
Probable Chronic Tension Type Headache107

cluster_128
Probable Hemicrania Continua2

cluster_3
exercise1

cluster_2
ndph1

cluster_129
Probable Hemicrania Continua3

cluster_115
Probable Chronic Tension Type Headache130
Probable Chronic Tension Type Headache85
Probable Chronic Tension Type Headache114
Probable Chronic Tension Type Headache90
Probable Chronic Tension Type Headache109
Probable Chronic Tension Type Headache132
Probable Chronic Tension Type Headache55
Probable Chronic Tension Type Headache112
Probable Chronic Tension Type Headache62
Probable Chronic Tension Type Headache59
Probable Chronic Tension Type Headache88

cluster_101
probable migraine with aura18

cluster_59
stabbing1

cluster_65
stabbing7

cluster_71
hypnic4

cluster_68
hypnic1

cluster_54
cm82
cm164
cm81
cm221
cm260
cm150
cm219
cm87
cm276
cm127
cm148

cm187
cm126
cm274
cm223
cm88
cm165
cm151
cm283
cm49
cm123
cm186
cm85
cm89
cm52
cm252
cm184
cm51
cm222
cm216
cm277
cm121
cm261
cm254
cm131
cm217
cm30
cm258
cm257
cm129

cluster_40
Probable Hemicrania Continua26
Probable Hemicrania Continua19
Probable Cluster Headache7
Probable Hemicrania Continua7
hc7

cluster_118
Probable Cluster Headache2

cluster_97
probable migraine with aura14

cluster_124
Probable Cluster Headache8

cluster_130
Probable Hemicrania Continua4

cluster_83

chronic tension type headache16
Probable Chronic Tension Type Headache60
Probable Chronic Tension Type Headache116
Probable Chronic Tension Type Headache44
chronic tension type headache4
chronic tension type headache42
chronic tension type headache30
Probable Chronic Tension Type Headache56
chronic tension type headache26
chronic tension type headache14
Probable Chronic Tension Type Headache12
Probable Chronic Tension Type Headache94
Probable Chronic Tension Type Headache8
chronic tension type headache28
Probable Chronic Tension Type Headache34
Probable Chronic Tension Type Headache10
Probable Chronic Tension Type Headache71
Probable Chronic Tension Type Headache32
chronic tension type headache38
Probable Chronic Tension Type Headache20
Probable Chronic Tension Type Headache97
Probable Chronic Tension Type Headache66
Probable Chronic Tension Type Headache30
chronic tension type headache6
Probable Chronic Tension Type Headache120
Probable Chronic Tension Type Headache52
Probable Chronic Tension Type Headache86
Probable Chronic Tension Type Headache99
Probable Chronic Tension Type Headache75
Probable Chronic Tension Type Headache122
chronic tension type headache18
Probable Chronic Tension Type Headache110
chronic tension type headache2

cluster_131
Probable Hemicrania Continua5

cluster_82
Probable Chronic Tension Type Headache93
Probable Chronic Tension Type Headache73
chronic tension type headache27
Probable Chronic Tension Type Headache31
chronic tension type headache17
Probable Chronic Tension Type Headache11
Probable Chronic Tension Type Headache54
chronic tension type headache29
Probable Chronic Tension Type Headache33
Probable Chronic Tension Type Headache70
Probable Chronic Tension Type Headache58
chronic tension type headache25

Probable Chronic Tension Type Headache18
Probable Chronic Tension Type Headache96
Probable Chronic Tension Type Headache7
chronic tension type headache13
Probable Chronic Tension Type Headache9
Probable Chronic Tension Type Headache43
Probable Chronic Tension Type Headache29
chronic tension type headache41
Probable Chronic Tension Type Headache108
Probable Chronic Tension Type Headache64
chronic tension type headache5
Probable Chronic Tension Type Headache84
chronic tension type headache1
chronic tension type headache37
Probable Chronic Tension Type Headache98
chronic tension type headache15
Probable Chronic Tension Type Headache121
Probable Chronic Tension Type Headache118
Probable Chronic Tension Type Headache115
Probable Chronic Tension Type Headache51
chronic tension type headache3

cluster_96
probable migraine with aura13

cluster_125
Probable Cluster Headache9

cluster_119
Probable Cluster Headache3

cluster_41
Probable Cluster Headache8
hc8
Probable Hemicrania Continua27
Probable Hemicrania Continua8
Probable Hemicrania Continua20

cluster_55
sex1

cluster_69
hypnic2

cluster_43
mwa32
probable migraine with aura38
mwa69
probable migraine with aura59
mwa31

mwa75
probable migraine with aura95
probable migraine with aura39
probable migraine with aura24
mwa17
mwa18
mwa35
mwa74
mwa14
probable migraine with aura44
probable migraine with aura41
probable migraine with aura26
mwa33
mwa2
mwa15
mwa4
probable migraine with aura43
probable migraine with aura40
probable migraine with aura58
probable migraine with aura25
mwa13
probable migraine with aura60
mwa100
mwa34
mwa36
probable migraine with aura101
probable migraine with aura61
probable migraine with aura62
probable migraine with aura57
mwa12
probable migraine with aura126
mwa1
probable migraine with aura1
mwa3
probable migraine with aura100
probable migraine with aura23

cluster_57
sex3

cluster_80
Probable Frequent Episodic Tension Type Headache64
Probable Frequent Episodic Tension Type Headache60
Probable Frequent Episodic Tension Type Headache31
frequent tension type headache11
frequent tension type headache7
frequent tension type headache5
Probable Frequent Episodic Tension Type Headache62
Probable Frequent Episodic Tension Type Headache41
frequent tension type headache9

Probable Frequent Episodic Tension Type Headache68
Probable Frequent Episodic Tension Type Headache39
Probable Frequent Episodic Tension Type Headache66
Probable Frequent Episodic Tension Type Headache76
Probable Frequent Episodic Tension Type Headache56
Probable Frequent Episodic Tension Type Headache6
frequent tension type headache13
Probable Frequent Episodic Tension Type Headache35
Probable Frequent Episodic Tension Type Headache37
Probable Frequent Episodic Tension Type Headache17
frequent tension type headache15
Probable Frequent Episodic Tension Type Headache29
Probable Frequent Episodic Tension Type Headache47
Probable Frequent Episodic Tension Type Headache70
Probable Frequent Episodic Tension Type Headache33
Probable Frequent Episodic Tension Type Headache51
frequent tension type headache19
frequent tension type headache3
frequent tension type headache17
frequent tension type headache21
Probable Frequent Episodic Tension Type Headache26
frequent tension type headache1
Probable Frequent Episodic Tension Type Headache54
Probable Frequent Episodic Tension Type Headache72

cluster_133
Probable Hemicrania Continua7

cluster_127
Probable Hemicrania Continua1

cluster_94
probable migraine with aura11

cluster_126
Probable Cluster Headache14

cluster_95
probable migraine with aura12

cluster_81
frequent tension type headache6
frequent tension type headache2
Probable Frequent Episodic Tension Type Headache55
frequent tension type headache10
Probable Frequent Episodic Tension Type Headache61
frequent tension type headache12
Probable Frequent Episodic Tension Type Headache40
Probable Frequent Episodic Tension Type Headache71
Probable Frequent Episodic Tension Type Headache30

Probable Frequent Episodic Tension Type Headache36
Probable Frequent Episodic Tension Type Headache57
Probable Frequent Episodic Tension Type Headache32
Probable Frequent Episodic Tension Type Headache67
Probable Frequent Episodic Tension Type Headache69
frequent tension type headache18
Probable Frequent Episodic Tension Type Headache38
frequent tension type headache22
Probable Frequent Episodic Tension Type Headache77
Probable Frequent Episodic Tension Type Headache63
frequent tension type headache20
Probable Frequent Episodic Tension Type Headache34
Probable Frequent Episodic Tension Type Headache42
Probable Frequent Episodic Tension Type Headache73
frequent tension type headache4
Probable Frequent Episodic Tension Type Headache18
frequent tension type headache14
Probable Frequent Episodic Tension Type Headache27
Probable Frequent Episodic Tension Type Headache7
Probable Frequent Episodic Tension Type Headache65
Probable Frequent Episodic Tension Type Headache48
Probable Frequent Episodic Tension Type Headache52
frequent tension type headache16
frequent tension type headache8

cluster_132
Probable Hemicrania Continua6

cluster_56
sex2

cluster_42
Probable Hemicrania Continua21
Probable Cluster Headache9
Probable Hemicrania Continua9
Probable Hemicrania Continua28
hc9

cluster_46
probable migraine with aura48
probable migraine with aura109
probable migraine with aura136
probable migraine with aura77
mwa48
probable migraine with aura144
probable migraine with aura72
probable migraine with aura111
mwa110
mwa22
mwa90

mwa118
probable migraine with aura74
mwa20
mwa46
probable migraine with aura4
mwa85
probable migraine with aura113
probable migraine with aura134
probable migraine with aura46
probable migraine with aura105
probable migraine with aura103
probable migraine with aura82
mwa83
probable migraine with aura84
mwa44
probable migraine with aura131
mwa87
mwa108
probable migraine with aura52
mwa7
probable migraine with aura70
mwa58
mwa51
mwa26
mwa56
mwa105
mwa77
mwa79
probable migraine with aura116
probable migraine with aura32

cluster_52
cm117
cm27
cm270
cm201
cm205
cm78
cm244
cm66
cm246
cm68
cm42
cm73
cm159
cm70
cm48
cm248
cm203
cm144

cm183
cm44
cm115
cm238
cm281
cm215
cm179
cm240
cm208
cm105
cm177
cm161
cm107
cm140
cm112
cm120
cm267
cm80
cm146
cm213
cm251
cm272

cluster_136
1 minute to 72 hours with severe

cluster_85
Probable Chronic Tension Type Headache106
chronic tension type headache24
Probable Chronic Tension Type Headache77
chronic tension type headache8
chronic tension type headache32
Probable Chronic Tension Type Headache22
Probable Chronic Tension Type Headache49
chronic tension type headache12
chronic tension type headache36
Probable Chronic Tension Type Headache19
Probable Chronic Tension Type Headache104
Probable Chronic Tension Type Headache92
Probable Chronic Tension Type Headache80
chronic tension type headache40
Probable Chronic Tension Type Headache119
Probable Chronic Tension Type Headache65
chronic tension type headache22
chronic tension type headache44
Probable Chronic Tension Type Headache14
Probable Chronic Tension Type Headache128
Probable Chronic Tension Type Headache74
Probable Chronic Tension Type Headache124
chronic tension type headache34

chronic tension type headache10
Probable Chronic Tension Type Headache27
Probable Chronic Tension Type Headache126
Probable Chronic Tension Type Headache82
Probable Chronic Tension Type Headache38
Probable Chronic Tension Type Headache36
Probable Chronic Tension Type Headache68
Probable Chronic Tension Type Headache101
chronic tension type headache20
Probable Chronic Tension Type Headache40

cluster_91
probable migraine with aura8

cluster_122
Probable Cluster Headache6

cluster_9
cluster2
Probable Cluster Headache15
Probable Cluster Headache11

cluster_8
cluster1
Probable Cluster Headache13
Probable Cluster Headache10

cluster_90
probable migraine with aura5

cluster_123
Probable Cluster Headache7

cluster_137
1 to 14 days per month

cluster_84
chronic tension type headache19
chronic tension type headache35
Probable Chronic Tension Type Headache103
Probable Chronic Tension Type Headache117
Probable Chronic Tension Type Headache100
Probable Chronic Tension Type Headache105
chronic tension type headache33
Probable Chronic Tension Type Headache21
Probable Chronic Tension Type Headache63
chronic tension type headache11
Probable Chronic Tension Type Headache13
chronic tension type headache23
Probable Chronic Tension Type Headache125

Probable Chronic Tension Type Headache91
Probable Chronic Tension Type Headache127
Probable Chronic Tension Type Headache35
Probable Chronic Tension Type Headache67
chronic tension type headache43
Probable Chronic Tension Type Headache79
chronic tension type headache9
chronic tension type headache31
Probable Chronic Tension Type Headache123
Probable Chronic Tension Type Headache72
Probable Chronic Tension Type Headache17
Probable Chronic Tension Type Headache26
Probable Chronic Tension Type Headache76
Probable Chronic Tension Type Headache48
Probable Chronic Tension Type Headache39
Probable Chronic Tension Type Headache37
chronic tension type headache21
Probable Chronic Tension Type Headache81
chronic tension type headache7
chronic tension type headache39

cluster_53
cm147
cm125
cm210
cm149
cm86
cm162
cm185
cm124
cm256
cm218
cm220
cm250
cm45
cm209
cm142
cm212
cm74
cm275
cm255
cm180
cm241
cm29
cm268
cm119
cm72
cm207
cm122
cm181

cm46
cm243
cm75
cm111
cm253
cm282
cm108
cm50
cm163
cm77
cm273
cm83

cluster_47
probable migraine with aura89
mwa23
probable migraine with aura137
mwa28
mwa95
probable migraine with aura121
mwa52
mwa55
probable migraine with aura34
probable migraine with aura78
probable migraine with aura87
mwa89
mwa24
probable migraine with aura6
probable migraine with aura79
mwa94
mwa113
mwa82
probable migraine with aura145
probable migraine with aura81
mwa50
probable migraine with aura50
probable migraine with aura139
probable migraine with aura120
probable migraine with aura76
probable migraine with aura108
mwa119
mwa63
probable migraine with aura49
probable migraine with aura132
probable migraine with aura106
mwa80
probable migraine with aura54
mwa8
mwa106
mwa111

probable migraine with aura118
probable migraine with aura115
mwa61
mwa53
mwa92

cluster_51
cm266
cm249
cm43
cm176
cm71
cm118
cm110
cm69
cm245
cm143
cm200
cm160
cm106
cm139
cm145
cm204
cm182
cm280
cm79
cm269
cm113
cm211
cm65
cm214
cm67
cm239
cm116
cm247
cm158
cm237
cm76
cm26
cm202
cm206
cm242
cm47
cm178
cm41
cm271
cm104

cluster_45
mwa43

probable migraine with aura69
mwa49
probable migraine with aura73
mwa25
probable migraine with aura47
probable migraine with aura83
mwa57
mwa6
probable migraine with aura135
mwa86
mwa109
mwa19
mwa104
mwa78
probable migraine with aura102
probable migraine with aura110
mwa45
mwa54
probable migraine with aura130
probable migraine with aura3
probable migraine with aura75
probable migraine with aura45
probable migraine with aura51
mwa107
probable migraine with aura114
probable migraine with aura104
mwa84
probable migraine with aura80
probable migraine with aura133
mwa81
probable migraine with aura143
mwa21
probable migraine with aura107
mwa47
probable migraine with aura71
probable migraine with aura31
mwa76
mwa88
probable migraine with aura112
mwa117

cluster_79
Probable Infrequent Episodic Tension Type Headache50
Probable Infrequent Episodic Tension Type Headache51
Probable Infrequent Episodic Tension Type Headache69
infrequent tension type headache9
Probable Infrequent Episodic Tension Type Headache55
Probable Infrequent Episodic Tension Type Headache68
Probable Infrequent Episodic Tension Type Headache14
Probable Infrequent Episodic Tension Type Headache63

infrequent tension type headache16
Probable Infrequent Episodic Tension Type Headache46
Probable Infrequent Episodic Tension Type Headache42
infrequent tension type headache5
Probable Infrequent Episodic Tension Type Headache41
Probable Infrequent Episodic Tension Type Headache59
Probable Infrequent Episodic Tension Type Headache70
Probable Infrequent Episodic Tension Type Headache56
Probable Infrequent Episodic Tension Type Headache73
infrequent tension type headache13
infrequent tension type headache6
infrequent tension type headache3
Probable Infrequent Episodic Tension Type Headache76
Probable Infrequent Episodic Tension Type Headache32
infrequent tension type headache15
infrequent tension type headache20
Probable Infrequent Episodic Tension Type Headache13
Probable Infrequent Episodic Tension Type Headache35
Probable Infrequent Episodic Tension Type Headache77
Probable Infrequent Episodic Tension Type Headache62
infrequent tension type headache4
Probable Infrequent Episodic Tension Type Headache37
Probable Infrequent Episodic Tension Type Headache72
Probable Infrequent Episodic Tension Type Headache53
Probable Infrequent Episodic Tension Type Headache38
Probable Infrequent Episodic Tension Type Headache21
Probable Infrequent Episodic Tension Type Headache3
infrequent tension type headache11
Probable Infrequent Episodic Tension Type Headache22
infrequent tension type headache19
infrequent tension type headache17
Probable Infrequent Episodic Tension Type Headache1
Probable Infrequent Episodic Tension Type Headache48
Probable Infrequent Episodic Tension Type Headache66
Probable Infrequent Episodic Tension Type Headache57
Probable Infrequent Episodic Tension Type Headache71
infrequent tension type headache22
infrequent tension type headache7
Probable Infrequent Episodic Tension Type Headache2
infrequent tension type headache14
Probable Infrequent Episodic Tension Type Headache43
Probable Infrequent Episodic Tension Type Headache52
Probable Infrequent Episodic Tension Type Headache75
infrequent tension type headache2
infrequent tension type headache10
Probable Infrequent Episodic Tension Type Headache11
Probable Infrequent Episodic Tension Type Headache74
Probable Infrequent Episodic Tension Type Headache45
Probable Infrequent Episodic Tension Type Headache34
Probable Infrequent Episodic Tension Type Headache24

Probable Infrequent Episodic Tension Type Headache58
infrequent tension type headache18
Probable Infrequent Episodic Tension Type Headache67
Probable Infrequent Episodic Tension Type Headache65
infrequent tension type headache1
Probable Infrequent Episodic Tension Type Headache33
Probable Infrequent Episodic Tension Type Headache23
Probable Infrequent Episodic Tension Type Headache10
infrequent tension type headache8
Probable Infrequent Episodic Tension Type Headache47
Probable Infrequent Episodic Tension Type Headache54
infrequent tension type headache21
Probable Infrequent Episodic Tension Type Headache49
infrequent tension type headache12
Probable Infrequent Episodic Tension Type Headache44
Probable Infrequent Episodic Tension Type Headache25
Probable Infrequent Episodic Tension Type Headache36
Probable Infrequent Episodic Tension Type Headache12
Probable Infrequent Episodic Tension Type Headache64

cluster_121
Probable Cluster Headache5

cluster_92
probable migraine with aura9

cluster_86
probable migraine without aura28
probable migraine without aura6
probable migraine without aura2

cluster_135
Probable Hemicrania Continua15

cluster_109
probable migraine with aura33

cluster_108
probable migraine with aura29

cluster_87
probable migraine without aura60
probable migraine without aura26
probable migraine without aura4
probable migraine without aura32

cluster_134
Probable Hemicrania Continua8

cluster_120

Probable Cluster Headache4

cluster_93
probable migraine with aura10

cluster_78
cough2

cluster_44
probable migraine with aura96
mwa5
mwa70
probable migraine with aura142
mwa72
mwa73
mwa10
mwa41
probable migraine with aura94
probable migraine with aura30
probable migraine with aura67
mwa40
probable migraine with aura93
mwa68
mwa37
probable migraine with aura98
mwa102
probable migraine with aura127
mwa116
probable migraine with aura68
mwa39
mwa67
mwa101
probable migraine with aura129
probable migraine with aura37
mwa11
mwa42
probable migraine with aura64
probable migraine with aura42
probable migraine with aura66
probable migraine with aura65
probable migraine with aura128
mwa103
probable migraine with aura36
probable migraine with aura97
probable migraine with aura2
mwa71
probable migraine with aura99
mwa16
probable migraine with aura63
mwa38

cluster_50
cm195
cm91
cm64
cm224
cm90
cm22
cm168
cm136
cm152
cm33
cm198
cm38
cm96
cm167
cm134
cm63
cm154
cm99
cm32
cm197
cm229
cm231
cm61
cm135
cm230
cm225
cm264
cm199
cm62
cm60
cm265
cm196
cm173
cm232
cm278
cm194
cm100
cm59
cm263
cm98

cluster_184
no lacrimation

cluster_37
hc4
Probable Hemicrania Continua4
Probable Hemicrania Continua23

Probable Hemicrania Continua13
Probable Cluster Headache4

cluster_23
Probable Cluster Headache32
ph7
Probable Cluster Headache29
Probable Cluster Headache35

cluster_190
no ptosis

cluster_147
at least one aura symptom is positive

cluster_153
brought on by cold stimuli
resolve within 30 min after removal of cold
greater than 2 episodes
fully reversible

cluster_152
brainstem aura

cluster_146
aggravated by physical activity

cluster_22
Probable Cluster Headache28
Probable Cluster Headache27
ph6
Probable Cluster Headache25

cluster_191
no restless

cluster_185
no miosis

cluster_36
Probable Hemicrania Continua18
Probable Hemicrania Continua3
Probable Hemicrania Continua12
Probable Cluster Headache3
hc3

cluster_193
nonpulsating

cluster_20

Probable Cluster Headache21
Probable Cluster Headache20
ph4
Probable Cluster Headache23

cluster_34
Probable Cluster Headache1
Probable Hemicrania Continua10
Probable Hemicrania Continua1
Probable Hemicrania Continua15
hc1

cluster_187
no orbital or supraorbital or temporal pain

cluster_178
motor aura

cluster_150
between 1 second to 2 hours
sudden

cluster_144
greater than 8 days per month
greater than 5 episodes
4 to 72 hours

cluster_145
abrupt explosive intensity just before or with orgasm

cluster_151
bilateral location

cluster_179
nasal congestion

cluster_35
Probable Hemicrania Continua17
Probable Cluster Headache2
hc2
Probable Hemicrania Continua2
Probable Hemicrania Continua11

cluster_186
no nasal congestion

cluster_192
no rhinorrhea

cluster_21

Probable Cluster Headache26
ph5
Probable Cluster Headache24
Probable Cluster Headache22

cluster_25
Probable Cluster Headache37
Probable Cluster Headache34
ph9
Probable Cluster Headache31

cluster_196
provoke by cough

cluster_182
no eyelid edema

cluster_31
Probable Hemicrania Continua21
Probable Hemicrania Continua20
sun6

cluster_19
Probable Cluster Headache18
Probable Cluster Headache15
ph3
Probable Cluster Headache17

cluster_155
brought on by sex

cluster_141
sharply contoured
1–6 cm in diameter
fixed in size and shape
round or elliptical

cluster_169
increasing in intensity with increasing sexual excitement

cluster_168
hours to days

cluster_140
15 to 180 minutes

cluster_154
less than 48 hours
brought on by exercise

cluster_18
Probable Cluster Headache16
Probable Cluster Headache12
Probable Cluster Headache14
ph2

cluster_183
no forehead and facial sweating

cluster_30
sun5
Probable Hemicrania Continua18
Probable Hemicrania Continua19

cluster_24
Probable Cluster Headache30
ph8
Probable Cluster Headache36
Probable Cluster Headache33

cluster_197
provoke by valsalva

cluster_208
unilateral

cluster_32
Probable Hemicrania Continua24
Probable Hemicrania Continua22
sun7

cluster_181
no conjunctival injection

cluster_195
phonophobia
photophobia

cluster_26
sun1
Probable Hemicrania Continua9
Probable Hemicrania Continua10

cluster_142
2 to 30 minutes

cluster_156
resolve within 1 hour after removal of compression
maximal at site of compression
brought on within 1 hour of compression

cluster_157
resolve within 1 hour after removal of traction
maximal at site of traction
brought on within 1 hour of traction

cluster_143
30 min to 7 days in duration

cluster_194
not aggrevated by activity

cluster_27
Probable Hemicrania Continua12
sun2
Probable Hemicrania Continua11

cluster_33
sun8
Probable Hemicrania Continua25
Probable Hemicrania Continua23

cluster_180
nausea/vomiting

cluster_209
up to 72 hours with mild

Parameter exploration for inflation = 3.3 and expansion = 9

cluster_5
no orbital or supraorbital or temporal pain

cluster_4
more than 1 episode per day

cluster_0
probable migraine with aura87
cm24
ph4
probable migraine with aura85
mwa26
probable migraine without aura18
probable migraine with aura21
probable migraine with aura54
probable migraine with aura27

probable migraine with aura17
cm25
cm248
mwa68
Probable Hemicrania Continua18
cm78
cm150
probable migraine with aura8
mwa34
cm233
probable migraine with aura52
mwa37
cm192
cm68
probable migraine with aura124
Probable Cluster Headache4
probable migraine without aura60
cm66
cm242
probable migraine with aura11
cluster8
probable migraine with aura64
cm147
cm176
cm54
cm116
cm185
Probable Cluster Headache29
mwa86
cm179
Probable Cluster Headache5
cm112
cm114
cm98
probable migraine with aura51
probable migraine with aura39
Probable Hemicrania Continua21
cm189
traction1
ph9
probable migraine with aura16
mwa99
cm23
probable migraine with aura67
probable migraine with aura9
mwa57
mwa65
cm57
cluster4
cm232

probable migraine without aura49
cm167
sun5
probable migraine without aura33
mwa104
Probable Hemicrania Continua8
mwa14
cm47
Probable Hemicrania Continua22
probable migraine without aura56
cm219
migraine w/o aura20
probable migraine with aura140
probable migraine with aura103
hc7
cm60
probable migraine without aura50
cm121
probable migraine without aura51
cm262
probable migraine without aura62
cm141
probable migraine with aura131
mwa97
probable migraine with aura123
migraine w/o aura2
cm71
cm267
mwa40
probable migraine with aura108
probable migraine with aura15
migraine w/o aura19
cm148
probable migraine without aura77
cm140
hc8
cm203
cm278
mwa94
probable migraine with aura10
Probable Hemicrania Continua27
probable migraine with aura90
thunderclap1
cm169
cm241
probable migraine with aura19
probable migraine with aura122
probable migraine without aura70
cm155
cm103

sun7
probable migraine with aura33
probable migraine without aura40
cm265
probable migraine without aura42
cluster1
probable migraine with aura106
cm44
mwa31
cm106
probable migraine without aura16
cm183
probable migraine with aura130
mwa20
cluster3
cm105
cm79
mwa98
sun3
cm161
cm175
cm37
cm284
probable migraine with aura25
mwa115
probable migraine with aura31
mwa67
mwa22
cm217
cm8
cm138
cm220
probable migraine without aura11
cm281
cm80
cm52
cm113
cm228
Probable Cluster Headache1
mwa63
probable migraine with aura56
cm260
cm89
probable migraine with aura59
cm166
cm223
cm198
probable migraine with aura29
cm61
cm206

cm152
Probable Cluster Headache6
cm144
mwa119
cm225
cm197
cm221
mwa107
probable migraine with aura110
cm28
probable migraine with aura1
probable migraine with aura47
probable migraine with aura79
mwa103
cm277
probable migraine without aura9
mwa102
hc6
cm177
cm276
migraine w/o aura17
cm137
Probable Hemicrania Continua28
cm227
probable migraine with aura113
mwa39
cm31
probable migraine with aura61
probable migraine without aura25
cm82
probable migraine with aura49
probable migraine with aura104
Probable Hemicrania Continua12
probable migraine with aura5
cm17
cm157
mwa53
probable migraine without aura22
mwa110
probable migraine without aura27
sex1
mwa1
mwa109
cm168
Probable Hemicrania Continua11
cm90
cough2
probable migraine with aura76
mwa23
mwa72

cm85
probable migraine with aura94
cm83
probable migraine without aura21
probable migraine with aura144
mwa90
cm261
mwa117
cm50
sex2
cm190
mwa6
probable migraine with aura114
cm193
probable migraine with aura32
hc9
cm4
probable migraine with aura84
probable migraine with aura12
probable migraine without aura53
cm59
cm246
Probable Cluster Headache37
cm257
cm56
cm16
mwa24
cm69
mwa3
mwa12
cm72
mwa89
cm48
cluster7
cm258
probable migraine with aura50
cm39
cm64
mwa8
Probable Cluster Headache14
cm270
cm92
cm5
cm124
Probable Cluster Headache33
cm251
probable migraine with aura23
cm86
cm222
cm41

cm245
cm156
cm239
cm250
cm275
probable migraine with aura38
hc5
probable migraine with aura107
mwa13
probable migraine with aura60
cm102
 migraine w/o aura4
probable migraine without aura39
cm118
cm127
cm280
probable migraine without aura8
mwa19
cm218
mwa74
probable migraine with aura129
ph8
cm164
cm201
probable migraine with aura24
mwa120
ph1
cm6
cm123
cm95
cm49
cm272
cm117
cm200
cluster5
cm283
mwa28
cm151
Probable Hemicrania Continua9
Probable Hemicrania Continua10
mwa25
sex3
probable migraine with aura7
probable migraine with aura134
probable migraine without aura37
cm186
probable migraine without aura58
cm204
probable migraine without aura1
cm62

cm11
cm235
cm26
cm22
probable migraine without aura13
probable migraine with aura98
probable migraine with aura142
mwa105
 migraine w/o aura12
cm40
cm178
 migraine w/o aura18
sun4
cm231
probable migraine with aura36
probable migraine with aura72
Probable Cluster Headache22
probable migraine with aura4
Probable Hemicrania Continua26
probable migraine without aura29
mwa81
ph7
mwa95
cm126
mwa29
cm253
probable migraine without aura63
probable migraine without aura72
probable migraine without aura76
probable migraine with aura132
probable migraine with aura40
probable migraine without aura10
cm128
probable migraine with aura3
cm259
probable migraine with aura28
mwa59
cm129
cm274
 migraine w/o aura22
probable migraine with aura75
mwa46
mwa10
Probable Hemicrania Continua23
cm3
Probable Cluster Headache16
probable migraine with aura119
mwa2
cm15
probable migraine without aura59

ph6
probable migraine with aura57
probable migraine without aura54
cm63
probable migraine with aura133
cm224
cm135
probable migraine with aura20
mwa71
Probable Hemicrania Continua2
Probable Hemicrania Continua5
mwa61
mwa96
cm207
probable migraine without aura26
probable migraine with aura70
Probable Cluster Headache7
Probable Cluster Headache27
cm208
mwa41
Probable Cluster Headache32
cm87
probable migraine with aura42
cm199
mwa18
probable migraine with aura46
probable migraine with aura73
probable migraine with aura135
cm165
probable migraine without aura5
Probable Hemicrania Continua14
exercise1
cm268
cm266
Probable Cluster Headache24
mwa79
probable migraine with aura137
mwa106
mwa30
cm101
mwa43
migraine w/o aura6
probable migraine with aura83
probable migraine with aura55
mwa11
mwa75
sex4
probable migraine without aura17
mwa52
cm2

probable migraine without aura52
probable migraine with aura95
Probable Cluster Headache3
cm58
migraine w/o aura16
probable migraine with aura13
probable migraine with aura116
mwa4
mwa47
Probable Hemicrania Continua6
cm216
cm171
cm131
cm172
Probable Cluster Headache8
mwa60
Probable Hemicrania Continua1
probable migraine without aura35
Probable Hemicrania Continua25
cm215
cm99
mwa7
cm34
probable migraine without aura2
cm211
probable migraine without aura31
cm182
cm213
mwa45
migraine w/o aura14
Probable Cluster Headache17
mwa83
cm76
probable migraine without aura55
migraine w/o aura3
cm279
probable migraine without aura7
probable migraine without aura74
mwa80
probable migraine without aura34
cm73
cm27
probable migraine with aura2
mwa56
Probable Cluster Headache2
Probable Cluster Headache12
migraine w/o aura10
mwa48
cm51
migraine w/o aura5

Probable Cluster Headache11
probable migraine with aura81
cm110
cm84
cm20
cluster9
cm96
cm230
Probable Cluster Headache13
Probable Cluster Headache35
probable migraine with aura41
mwa73
mwa76
cm154
probable migraine with aura121
cm249
probable migraine without aura57
cm65
cm122
cm53
cm196
probable migraine with aura6
probable migraine with aura30
probable migraine with aura62
mwa64
Probable Cluster Headache23
sun1
cm55
cm81
probable migraine with aura91
cm45
Probable Cluster Headache21
probable migraine with aura69
Probable Cluster Headache25
probable migraine without aura32
probable migraine with aura88
probable migraine without aura41
cm263
mwa49
probable migraine with aura111
probable migraine without aura28
probable migraine with aura105
cm146
cm36
hc4
probable migraine with aura71
Probable Cluster Headache31
cm94
cm9
cough1

cm93
probable migraine with aura109
ph5
mwa58
cm162
Probable Hemicrania Continua19
cm142
probable migraine without aura43
compression1
mwa16
mwa66
cm109
probable migraine with aura128
mwa51
probable migraine with aura63
mwa85
probable migraine without aura71
probable migraine without aura24
probable migraine with aura101
sun2
mwa78
mwa111
probable migraine with aura86
cm153
cm238
probable migraine without aura45
probable migraine without aura65
probable migraine with aura80
probable migraine without aura20
cm244
mwa35
probable migraine with aura92
mwa112
cm252
mwa27
Probable Hemicrania Continua3
mwa5
cm97
probable migraine with aura35
probable migraine without aura44
probable migraine with aura120
cluster2
cm19
cm234
cm149
cm264
probable migraine without aura61
cm143
mwa116
cm273

probable migraine without aura15
Probable Cluster Headache34
probable migraine with aura96
mwa87
probable migraine with aura45
mwa70
mwa114
probable migraine with aura22
Probable Hemicrania Continua16
mwa93
probable migraine without aura68
probable migraine with aura44
cm30
cm226
probable migraine with aura102
probable migraine with aura97
cm282
mwa92
cm174
probable migraine with aura93
hc2
Probable Cluster Headache10
cm163
migraine w/o aura8
cm136
cm91
cm214
probable migraine with aura141
probable migraine with aura37
probable migraine with aura99
probable migraine without aura48
probable migraine with aura126
Probable Hemicrania Continua24
probable migraine with aura14
cm100
probable migraine with aura145
mwa32
mwa77
probable migraine with aura18
cm247
mwa38
cm181
probable migraine without aura4
Probable Cluster Headache26
cm35
probable migraine with aura100
probable migraine with aura58
Probable Cluster Headache20
cm108
probable migraine with aura43

Probable Hemicrania Continua4
probable migraine without aura19
migraine w/o aura9
mwa17
cm132
migraine w/o aura21
probable migraine without aura64
mwa118
mwa113
probable migraine without aura38
probable migraine with aura68
probable migraine with aura138
Probable Cluster Headache30
migraine w/o aura1
cm187
cm159
cluster6
cm243
cm188
mwa42
mwa33
probable migraine without aura30
migraine w/o aura7
probable migraine with aura66
Probable Cluster Headache28
migraine w/o aura13
mwa69
Probable Cluster Headache18
probable migraine with aura146
Probable Hemicrania Continua20
mwa84
cm202
Probable Hemicrania Continua13
probable migraine with aura65
cm205
cm104
migraine w/o aura11
Probable Hemicrania Continua15
mwa55
mwa82
cm145
cm139
probable migraine without aura12
probable migraine without aura23
mwa50
cm120
sun6
cm254
cm43
cm111

probable migraine without aura14
probable migraine with aura53
cm21
mwa44
mwa21
cm170
cm237
cm256
hc3
probable migraine with aura78
hc1
cm88
cm46
Probable Cluster Headache19
cm125
cm158
cm10
cm236
probable migraine with aura48
Probable Cluster Headache15
cm130
mwa108
cm14
cm74
probable migraine without aura3
probable migraine with aura82
coldHA1
cm115
cm134
cm240
mwa100
probable migraine with aura139
cm119
mwa9
cm1
cm13
cm180
probable migraine without aura69
cm33
probable migraine without aura6
mwa88
probable migraine with aura115
migraine w/o aura15
cm77
cm194
probable migraine without aura67
mwa62
cm42
probable migraine with aura125
mwa15

probable migraine with aura74
cm67
Probable Hemicrania Continua7
cm195
cm210
probable migraine without aura75
cm209
probable migraine with aura117
cm160
cm269
probable migraine without aura73
probable migraine without aura36
cm12
cm32
cm70
mwa101
cm271
probable migraine without aura46
probable migraine without aura66
probable migraine without aura47
mwa36
cm212
cm7
cm107
probable migraine with aura143
cm255
cm133
probable migraine with aura136
ph2
cm173
cm38
Probable Cluster Headache9
cm191
Probable Hemicrania Continua17
cm18
cm29
mwa91
cm184
probable migraine with aura118
Probable Cluster Headache36
sun8
probable migraine with aura77
probable migraine with aura89
probable migraine with aura34
probable migraine with aura112
probable migraine with aura127
mwa54
cm75
probable migraine with aura26
cm229

ph3
resolve within 30 min after removal of cold
miosis
at least one aura symptom spreads gradually over 5 minutes
abrupt explosive intensity just before or with orgasm
brought on within 1 hour of compression
greater than 5 minutes
the aura is accompanied, or followed within 60 minutes, by headache
rhinorrhea
ptosis
brought on by exercise
4 to 72 hours
at least one aura symptom is unilateral
orbital or supraorbital or temporal pain
indomethacin responsive
retinal aura
greater than 2 episodes
eyelid edema
phonophobia
brought on by cold stimuli
each individual aura symptom lasts 5–60 minutes
restless
sudden
up to 72 hours with mild
at least one aura symptom is positive
forehead and facial sweating
greater than 8 days per month
severe
motor aura
brought on by sex
brought on within 1 hour of traction
greater than 5 episodes
lacrimation
photophobia
provoke by cough
resolve within 1 hour after removal of traction
nasal congestion
visual aura
1 minute to 72 hours with severe
maximal at site of compression
less than 48 hours
relieve by triptan or ergot
resolve within 1 hour after removal of compression
max within 1 minute
pulsating
sensory aura
increasing in intensity with increasing sexual excitement
two or more aura symptoms occur in succession
greater than 1 per day
unilateral

moderate to severe
nausea/vomiting
every other day to 8 per day
2 to 30 minutes
maximal at site of traction
conjunctival injection
1 to 600 seconds
greater than 15 days per month
speech and/or language aura
15 to 180 minutes
aggravated by physical activity
provoke by valsalva
greater than 20 episodes
between 1 second to 2 hours
fully reversible
greater than 5 per day
brainstem aura

cluster_1
ndph1

cluster_3
nummular1
sharply contoured
fixed in size and shape
round or elliptical
1–6 cm in diameter

cluster_2
Probable Chronic Tension Type Headache48
chronic tension type headache8
hypnic5
Probable Chronic Tension Type Headache67
Probable Infrequent Episodic Tension Type Headache73
Probable Frequent Episodic Tension Type Headache24
Probable Infrequent Episodic Tension Type Headache25
Probable Chronic Tension Type Headache108
Probable Chronic Tension Type Headache58
chronic tension type headache33
chronic tension type headache2
Probable Frequent Episodic Tension Type Headache56
Probable Chronic Tension Type Headache65
Probable Infrequent Episodic Tension Type Headache31
Probable Infrequent Episodic Tension Type Headache71
Probable Frequent Episodic Tension Type Headache48
stabbing2
Probable Infrequent Episodic Tension Type Headache65
Probable Chronic Tension Type Headache68
Probable Frequent Episodic Tension Type Headache73
Probable Chronic Tension Type Headache30

chronic tension type headache37
Probable Frequent Episodic Tension Type Headache40
Probable Chronic Tension Type Headache123
Probable Frequent Episodic Tension Type Headache14
Probable Chronic Tension Type Headache27
Probable Chronic Tension Type Headache23
frequent tension type headache8
infrequent tension type headache2
Probable Frequent Episodic Tension Type Headache20
Probable Chronic Tension Type Headache47
Probable Chronic Tension Type Headache86
Probable Infrequent Episodic Tension Type Headache46
frequent tension type headache10
infrequent tension type headache3
Probable Infrequent Episodic Tension Type Headache5
Probable Infrequent Episodic Tension Type Headache45
Probable Chronic Tension Type Headache129
Probable Chronic Tension Type Headache131
Probable Chronic Tension Type Headache34
Probable Infrequent Episodic Tension Type Headache2
Probable Chronic Tension Type Headache106
Probable Frequent Episodic Tension Type Headache42
Probable Chronic Tension Type Headache111
frequent tension type headache2
stabbing1
frequent tension type headache22
Probable Chronic Tension Type Headache117
Probable Chronic Tension Type Headache85
chronic tension type headache43
Probable Infrequent Episodic Tension Type Headache36
frequent tension type headache19
frequent tension type headache3
infrequent tension type headache22
Probable Chronic Tension Type Headache75
Probable Chronic Tension Type Headache55
Probable Frequent Episodic Tension Type Headache23
Probable Chronic Tension Type Headache98
Probable Chronic Tension Type Headache37
Probable Infrequent Episodic Tension Type Headache10
chronic tension type headache36
Probable Chronic Tension Type Headache36
chronic tension type headache10
chronic tension type headache17
Probable Chronic Tension Type Headache113
Probable Chronic Tension Type Headache32
Probable Infrequent Episodic Tension Type Headache11
Probable Chronic Tension Type Headache82
chronic tension type headache42
Probable Infrequent Episodic Tension Type Headache42
chronic tension type headache32

infrequent tension type headache17
Probable Infrequent Episodic Tension Type Headache1
Probable Frequent Episodic Tension Type Headache39
Probable Infrequent Episodic Tension Type Headache22
frequent tension type headache13
hypnic1
Probable Infrequent Episodic Tension Type Headache56
Probable Frequent Episodic Tension Type Headache45
Probable Chronic Tension Type Headache33
Probable Frequent Episodic Tension Type Headache27
Probable Frequent Episodic Tension Type Headache51
Probable Infrequent Episodic Tension Type Headache60
Probable Frequent Episodic Tension Type Headache11
Probable Chronic Tension Type Headache101
Probable Chronic Tension Type Headache105
Probable Chronic Tension Type Headache28
infrequent tension type headache14
Probable Frequent Episodic Tension Type Headache68
Probable Chronic Tension Type Headache41
Probable Infrequent Episodic Tension Type Headache64
Probable Chronic Tension Type Headache116
chronic tension type headache25
Probable Chronic Tension Type Headache19
Probable Infrequent Episodic Tension Type Headache4
Probable Frequent Episodic Tension Type Headache47
Probable Infrequent Episodic Tension Type Headache57
Probable Infrequent Episodic Tension Type Headache13
Probable Chronic Tension Type Headache22
hypnic2
Probable Frequent Episodic Tension Type Headache9
Probable Frequent Episodic Tension Type Headache54
Probable Chronic Tension Type Headache89
infrequent tension type headache18
Probable Frequent Episodic Tension Type Headache50
frequent tension type headache7
Probable Chronic Tension Type Headache12
Probable Chronic Tension Type Headache92
Probable Infrequent Episodic Tension Type Headache3
Probable Frequent Episodic Tension Type Headache69
Probable Frequent Episodic Tension Type Headache76
Probable Infrequent Episodic Tension Type Headache17
Probable Chronic Tension Type Headache53
Probable Infrequent Episodic Tension Type Headache24
Probable Chronic Tension Type Headache71
Probable Infrequent Episodic Tension Type Headache74
Probable Infrequent Episodic Tension Type Headache63
Probable Frequent Episodic Tension Type Headache74
Probable Infrequent Episodic Tension Type Headache38
Probable Frequent Episodic Tension Type Headache55
chronic tension type headache19

hypnic8
Probable Chronic Tension Type Headache94
Probable Infrequent Episodic Tension Type Headache40
chronic tension type headache21
Probable Chronic Tension Type Headache17
frequent tension type headache14
Probable Chronic Tension Type Headache5
Probable Infrequent Episodic Tension Type Headache29
chronic tension type headache7
Probable Chronic Tension Type Headache124
Probable Chronic Tension Type Headache100
chronic tension type headache4
infrequent tension type headache19
chronic tension type headache16
chronic tension type headache38
Probable Frequent Episodic Tension Type Headache32
Probable Infrequent Episodic Tension Type Headache30
hypnic3
chronic tension type headache14
Probable Frequent Episodic Tension Type Headache2
Probable Chronic Tension Type Headache13
Probable Infrequent Episodic Tension Type Headache59
Probable Frequent Episodic Tension Type Headache29
Probable Chronic Tension Type Headache31
Probable Chronic Tension Type Headache6
infrequent tension type headache5
Probable Frequent Episodic Tension Type Headache7
Probable Chronic Tension Type Headache126
Probable Infrequent Episodic Tension Type Headache72
frequent tension type headache9
chronic tension type headache24
Probable Chronic Tension Type Headache18
Probable Infrequent Episodic Tension Type Headache21
Probable Chronic Tension Type Headache20
Probable Chronic Tension Type Headache72
chronic tension type headache41
infrequent tension type headache16
Probable Infrequent Episodic Tension Type Headache50
Probable Infrequent Episodic Tension Type Headache70
frequent tension type headache11
Probable Infrequent Episodic Tension Type Headache39
Probable Frequent Episodic Tension Type Headache31
Probable Chronic Tension Type Headache56
chronic tension type headache27
Probable Chronic Tension Type Headache46
Probable Frequent Episodic Tension Type Headache25
Probable Chronic Tension Type Headache88
Probable Chronic Tension Type Headache59
Probable Frequent Episodic Tension Type Headache33
Probable Chronic Tension Type Headache84

Probable Infrequent Episodic Tension Type Headache6
Probable Chronic Tension Type Headache104
Probable Chronic Tension Type Headache103
Probable Chronic Tension Type Headache45
Probable Chronic Tension Type Headache50
Probable Chronic Tension Type Headache3
Probable Frequent Episodic Tension Type Headache30
frequent tension type headache5
Probable Frequent Episodic Tension Type Headache38
stabbing6
Probable Infrequent Episodic Tension Type Headache54
Probable Chronic Tension Type Headache2
Probable Chronic Tension Type Headache95
chronic tension type headache12
chronic tension type headache13
Probable Chronic Tension Type Headache115
infrequent tension type headache8
Probable Infrequent Episodic Tension Type Headache47
Probable Chronic Tension Type Headache60
Probable Chronic Tension Type Headache107
Probable Infrequent Episodic Tension Type Headache75
Probable Chronic Tension Type Headache78
frequent tension type headache6
frequent tension type headache17
Probable Chronic Tension Type Headache96
Probable Chronic Tension Type Headache4
Probable Chronic Tension Type Headache57
Probable Frequent Episodic Tension Type Headache72
Probable Chronic Tension Type Headache1
infrequent tension type headache20
Probable Frequent Episodic Tension Type Headache75
Probable Chronic Tension Type Headache42
chronic tension type headache6
Probable Frequent Episodic Tension Type Headache28
Probable Chronic Tension Type Headache90
frequent tension type headache21
chronic tension type headache1
chronic tension type headache9
Probable Infrequent Episodic Tension Type Headache16
Probable Frequent Episodic Tension Type Headache13
Probable Chronic Tension Type Headache61
Probable Chronic Tension Type Headache39
Probable Frequent Episodic Tension Type Headache4
Probable Chronic Tension Type Headache51
Probable Chronic Tension Type Headache76
Probable Chronic Tension Type Headache97
Probable Chronic Tension Type Headache121
chronic tension type headache20
Probable Infrequent Episodic Tension Type Headache12
Probable Chronic Tension Type Headache29

Probable Chronic Tension Type Headache127
Probable Chronic Tension Type Headache54
Probable Infrequent Episodic Tension Type Headache33
Probable Frequent Episodic Tension Type Headache19
Probable Frequent Episodic Tension Type Headache77
Probable Frequent Episodic Tension Type Headache71
Probable Chronic Tension Type Headache102
Probable Frequent Episodic Tension Type Headache3
Probable Frequent Episodic Tension Type Headache5
Probable Infrequent Episodic Tension Type Headache28
Probable Chronic Tension Type Headache44
Probable Chronic Tension Type Headache93
Probable Chronic Tension Type Headache64
Probable Frequent Episodic Tension Type Headache52
Probable Frequent Episodic Tension Type Headache49
chronic tension type headache35
Probable Infrequent Episodic Tension Type Headache68
Probable Frequent Episodic Tension Type Headache59
Probable Chronic Tension Type Headache132
hypnic7
frequent tension type headache1
infrequent tension type headache21
Probable Infrequent Episodic Tension Type Headache76
Probable Frequent Episodic Tension Type Headache43
frequent tension type headache18
chronic tension type headache31
Probable Frequent Episodic Tension Type Headache41
Probable Chronic Tension Type Headache91
Probable Frequent Episodic Tension Type Headache57
infrequent tension type headache1
Probable Chronic Tension Type Headache125
Probable Chronic Tension Type Headache122
Probable Frequent Episodic Tension Type Headache36
infrequent tension type headache12
Probable Frequent Episodic Tension Type Headache60
Probable Frequent Episodic Tension Type Headache70
Probable Infrequent Episodic Tension Type Headache53
Probable Chronic Tension Type Headache120
Probable Frequent Episodic Tension Type Headache34
hypnic6
Probable Chronic Tension Type Headache43
Probable Frequent Episodic Tension Type Headache1
hypnic9
stabbing7
Probable Infrequent Episodic Tension Type Headache52
Probable Infrequent Episodic Tension Type Headache20
chronic tension type headache23
Probable Infrequent Episodic Tension Type Headache51
hypnic4
frequent tension type headache16

Probable Frequent Episodic Tension Type Headache22
Probable Chronic Tension Type Headache38
Probable Frequent Episodic Tension Type Headache8
Probable Chronic Tension Type Headache83
Probable Frequent Episodic Tension Type Headache10
Probable Frequent Episodic Tension Type Headache17
infrequent tension type headache4
Probable Chronic Tension Type Headache40
chronic tension type headache28
Probable Infrequent Episodic Tension Type Headache8
stabbing8
chronic tension type headache34
chronic tension type headache3
Probable Chronic Tension Type Headache52
infrequent tension type headache7
Probable Frequent Episodic Tension Type Headache26
Probable Chronic Tension Type Headache81
Probable Chronic Tension Type Headache74
Probable Chronic Tension Type Headache8
Probable Frequent Episodic Tension Type Headache16
Probable Infrequent Episodic Tension Type Headache66
Probable Chronic Tension Type Headache24
Probable Infrequent Episodic Tension Type Headache34
Probable Frequent Episodic Tension Type Headache35
Probable Chronic Tension Type Headache114
Probable Infrequent Episodic Tension Type Headache69
Probable Frequent Episodic Tension Type Headache65
Probable Frequent Episodic Tension Type Headache58
Probable Chronic Tension Type Headache7
Probable Chronic Tension Type Headache35
Probable Infrequent Episodic Tension Type Headache35
Probable Frequent Episodic Tension Type Headache53
Probable Frequent Episodic Tension Type Headache67
infrequent tension type headache9
Probable Infrequent Episodic Tension Type Headache26
Probable Chronic Tension Type Headache9
Probable Frequent Episodic Tension Type Headache15
Probable Chronic Tension Type Headache128
Probable Frequent Episodic Tension Type Headache62
Probable Frequent Episodic Tension Type Headache21
stabbing4
frequent tension type headache20
Probable Infrequent Episodic Tension Type Headache37
Probable Infrequent Episodic Tension Type Headache43
Probable Frequent Episodic Tension Type Headache64
Probable Chronic Tension Type Headache118
infrequent tension type headache10
Probable Chronic Tension Type Headache69
chronic tension type headache44
Probable Infrequent Episodic Tension Type Headache27

Probable Frequent Episodic Tension Type Headache63
Probable Chronic Tension Type Headache15
Probable Chronic Tension Type Headache21
Probable Chronic Tension Type Headache10
Probable Infrequent Episodic Tension Type Headache44
Probable Chronic Tension Type Headache66
chronic tension type headache40
Probable Infrequent Episodic Tension Type Headache18
infrequent tension type headache11
Probable Infrequent Episodic Tension Type Headache41
infrequent tension type headache6
Probable Chronic Tension Type Headache11
Probable Infrequent Episodic Tension Type Headache67
Probable Chronic Tension Type Headache73
chronic tension type headache22
chronic tension type headache26
Probable Infrequent Episodic Tension Type Headache48
Probable Frequent Episodic Tension Type Headache46
Probable Chronic Tension Type Headache26
Probable Infrequent Episodic Tension Type Headache77
Probable Chronic Tension Type Headache49
Probable Chronic Tension Type Headache109
Probable Chronic Tension Type Headache130
Probable Frequent Episodic Tension Type Headache12
Probable Frequent Episodic Tension Type Headache18
Probable Infrequent Episodic Tension Type Headache23
chronic tension type headache29
chronic tension type headache30
Probable Infrequent Episodic Tension Type Headache62
infrequent tension type headache15
Probable Chronic Tension Type Headache77
Probable Chronic Tension Type Headache87
stabbing3
Probable Chronic Tension Type Headache119
stabbing5
Probable Chronic Tension Type Headache112
frequent tension type headache15
Probable Infrequent Episodic Tension Type Headache14
Probable Chronic Tension Type Headache16
Probable Infrequent Episodic Tension Type Headache15
frequent tension type headache12
Probable Infrequent Episodic Tension Type Headache55
infrequent tension type headache13
chronic tension type headache11
Probable Frequent Episodic Tension Type Headache37
Probable Chronic Tension Type Headache79
Probable Infrequent Episodic Tension Type Headache9
Probable Chronic Tension Type Headache14
Probable Infrequent Episodic Tension Type Headache61
Probable Infrequent Episodic Tension Type Headache7
