# Supplementary material for: Matrix and graphical representation of the primary headache syndromes in the International Classification of Headache Disorders (ICHD3): a basis for automated diagnosis and analysis of criteria
Source: Front Neurol. 2026 May 11;17:1812996. doi: 10.3389/fneur.2026.1812996 (PMC13200560; doi:10.3389/fneur.2026.1812996)
Supplement: Supplementary file 12 [file Data_Sheet_12.pdf]

Proofs for automated diagnosis using matrix representation of ICHD3

Pengfei Zhang, MD

December 22, 2025

1 Introduction

We propose that the International Classification of Headache Disorders (ICHD3) can be translated into mathematical data in the form of matrix. We will demonstrate that not only can this interpretation allow us to automate diagnosis of headache disorders, but this technique also allows us to efficiently screen out those headache phenotypes that can be diagnosed by the international classification.

2 Automated Diagnosis using ICHD3 Matrix

In the international criteria, specific headache disorders are defined by a constellation of characteristics. Each unique combination of characteristics forms what we call a “headache phenotype”.

Definition 2.1. *A matrix representation of ICHD3, denoted in this document as R, is defined as a matrix where each row, i, represents a phenotype in the ICHD3 and each column, j, represents a characteristics. The element of the matrix R_{ij} takes on the value of 1 if the phenotype i contains the characteristics j. Otherwise R_{ij} takes on the value of 0. We further call the ith phenotype in this matrix R_i.*

This is the ”diagnosis matrix” in our poster.

Definition 2.2. *A vector representation of headache phenotype, M, is defined as a vector where each element, i, represents the presence or absence of a characteristics. This element takes on the value of 1 if the characteristic is present; otherwise it takes on the value of 0. We demand that the ordering of characteristics in M corresponds to the ordering of phenotype in the matrix R.*

A phenotype M is considered diagnosable by a specific R_k, if for every element of R_k that is 1, the corresponding element in M is also 1. That is,

Definition 2.3. *M is diagnosable by R_k if $\forall R_{ki} = 1$, $M_i = 1$,*

This is a simple way of saying that if every characteristic that is present in a diagnostic criteria is in a patient’s phenotype, then the patient’s phenotype can be diagnosed with that diagnostic criteria. Notice that the converse of the above need not be true.

Theorem 2.1. *M is diagnosable by R_k if and only if $\forall i, R_{ki} * M_i = R_{ki}$.*

Proof. We will first prove the backward direction:
Let $R_{ki} * M_i = R_{ki}$ for all i. By definition 2.3, we simply need to prove that $M_i = 1$ whenever $R_{ki} = 1$. Therefore let $R_{ki} = 1$ then $R_{ki} * M_i = 1 * M_i = R_{ki} = 1$.
Now we will prove the forward direction:
Let M be diagnosable by R_k. Then by definition 2.3, $\forall R_{ki} = 1$, $M_i = 1$. So $\forall i, R_{ki} * M_i = 1 * 1 = R_{ki}$ □

Now we can make the following claim:

Theorem 2.2. *Let S_k be the row sum of R_k, then assuming M is not the zero vector, then $\sum_i R_{ki} * M_i = S_k$ if and only if M can be diagnosed by R_k.*

Proof. The proof will require both the forward and the backward direction. We will first prove the backward direction:

Assume M is diagnosed with the R_k. Then by theorem 2.1, $R_{ki} * M_i = R_{ki}, \forall i$. Write out both sides term by term, where r is an element of R_k and m is an element of M

$$[r_1 * m_1, r_2 * m_2, \dots .m_n] = [r_1, r_2, r_3..r_n]$$

This implies that,

$$r_1 * m_1 + r_2 * m_2 + \dots .m_n = r_1 + r_2 + r_3 + \dots r_n$$

This proves the backward part of the claim since $r_1 + r_2 + r_3 + \dots r_n = S_k$ by definition.

We will now prove the forward component:

Assume $r_1 * m_1 + r_2 * m_2 + \dots + r_j * m_j \dots . + r_n * m_n = r_1 + r_2 + r_3 + \dots + r_j \dots + r_n$ Subtract the right side from the left: $r_1 * (m_1 - 1) + r_2 * (m_2 - 1) + \dots + r_j * (m_j - 1) \dots . + r_n * (m_n – 1) = 0$

Since each of $m_1, m_2, \dots m_n$ as well as $r_1, r_2 \dots r_n$ are either 0 or 1, then the maximum value of each term above is 0. So in order for the above equation to be true, whenever $m_j = 0$, that corresponding r_j must also equal 0. (Otherwise the equation would be negative on the left side if even one term is negative.) So $m_j = 0$ implies $r_j = 0$. Since r only takes on 0 or 1, the contrapositive is also true: i.e. whenever $r_j = 1$, it implies $m_j = 1$.

So then:
Case 1: if $r_j = 0$, then $r_j * m_j = 0$, so $r_j * m_j = r_j$.
Case 2: if $r_j = 1$, then $m_j = 1$ by the above, then $r_j * m_j = 1$, then $r_j * m_j = r_j$.
Case 3: if $m_j = 0$, then by above, $r_j = 0$, this implies $r_j * m_j = 0$, so $r_j * m_j = r_j$.
Case 4: if $m_j = 1$, then $r_j * m_j = r_j * 1 = r_j$.

This implies $r_j * m_j = r_j$ for all cases. This proves the forward component. □

The following observation is immediately obvious:

Theorem 2.3. *M is diagnosable by the row(s) in R where the corresponding row(s) in $R \cdot M = S_k$.*

Proof. Given a M and R and call V the result of $R \cdot M$. If the row sum of kth row of R, S_k, is equal to the k row of V. Then by definition of dot product, this is equivalent to $S_k = \sum_i R_{ki} * M_i$. By theorem 2.2, the kth row is diagnosable. □

Using the above observation, we can characterize whether a specific M is diagnosable by ICHD3 through the following:

Definition 2.4. *Let S be a vector where each ith element represents the ith row sum of R.*

Theorem 2.4. *Let V be the result of $R \cdot M$ and let $V - S = V'$. Then if M is diagnosable by R, $\prod_i V' = 0$.*

Proof. By theorem 2.3, each row of V that is diagnosable by its corresponding R is S_k. So that corresponding row of $V - S$ is 0. As a result, if M is diagnosable then there is a 0 in V'. So $\prod_i V' = 0$. □

The contrapositive of the last theorem produces the following:

Theorem 2.5. *If $\prod_i V'$ does not equal 0 then M is not diagnosable by R.*

3 Extending Automated Diagnosis to Multiple Phenotypes

Theorem 2.3 allow us to automate diagnosis of any given vector representation of headache phenotype by checking a dot product and a row sum. Intuitively, this methodology does not need to be limited to only one headache phenotype. This is of particular importance since our project involves a large set of phenotype.

Definition 3.1. *Similar to the "diagnosis matrix" we define a "phenotype matrix", P, as a matrix where each row, i, represents a phenotype and each column, j, represents a characteristics. The element of the matrix R_{ij} takes on the value of 1 if the phenotype i contains the characteristics j. Otherwise R_{ij} takes on the value of 0. We will also call P' the transpose of (P).*

Each row/phenotype in P may take on any arbitrary list of values, unlike R, which is restricted to those phenotypes represented in the ICHD3. Althought this is a more intuitive and convenient definition of phenotype matrix, in order to use the dot product observation developed in the last section, it is more convenient to use P' since then each column of P' corresponds to a vector M in the last section.

Theorem 3.1. *Calculate $I = R \cdot P' - S$. All columns of I, I_j, where $\prod_j I_j$ does not equal 0 are not diagnosable.*

Proof. This proceeds immediately from theorem 2.5 and definition of matrix multiplication. □
