# Supplementary material for: Matrix and graphical representation of the primary headache syndromes in the International Classification of Headache Disorders (ICHD3): a basis for automated diagnosis and analysis of criteria
Source: Front Neurol. 2026 May 11;17:1812996. doi: 10.3389/fneur.2026.1812996 (PMC13200560; doi:10.3389/fneur.2026.1812996)
Supplement: Supplementary file 13 [file Data_Sheet_13.pdf]

TAC:

cluster_8
ph1

cluster_9
ph2

cluster_10
ph3

cluster_11
ph4

cluster_12
ph5

cluster_13
ph6

cluster_14
ph7

cluster_15
ph8

cluster_16
ph9

cluster_17
sun6
Probable Hemicrania Continua13
Probable Hemicrania Continua16
sun1
Probable Hemicrania Continua11
sun2
Probable Hemicrania Continua18
sun4
Probable Hemicrania Continua15
sun5
Probable Hemicrania Continua20
sun8
sun7
Probable Hemicrania Continua22
Probable Hemicrania Continua9
Probable Hemicrania Continua23
sun3
probable migraine without aura13
greater than 1 per day
1 to 600 seconds

cluster_7
cluster8

cluster_3
Probable Cluster Headache14
Probable Cluster Headache18
thunderclap1
cluster1
cluster6
cluster2
Probable Cluster Headache19
cluster3
Probable Cluster Headache25
Probable Cluster Headache23
cluster4
Probable Cluster Headache12
Probable Cluster Headache10
Probable Cluster Headache11
cluster9
cluster5
Probable Cluster Headache17
cluster7
max within 1 minute
greater than 5 minutes

cluster_22
hc5
Probable Cluster Headache24
Probable Hemicrania Continua19
Probable Cluster Headache21
Probable Hemicrania Continua14
Probable Cluster Headache5
Probable Hemicrania Continua5
Probable Hemicrania Continua24
greater than 5 per day
miosis

cluster_23
Probable Hemicrania Continua16
hc6
Probable Cluster Headache22
Probable Hemicrania Continua25
Probable Hemicrania Continua21
Probable Cluster Headache27
Probable Cluster Headache6
Probable Hemicrania Continua6

greater than 20 episodes
nasal congestion

cluster_20
Probable Hemicrania Continua3
Probable Hemicrania Continua18
hc3
Probable Hemicrania Continua14
Probable Hemicrania Continua12
Probable Cluster Headache16
Probable Cluster Headache17
Probable Cluster Headache3
forehead and facial sweating
indomethacin responsive

cluster_21
Probable Hemicrania Continua13
Probable Cluster Headache4
Probable Cluster Headache20
Probable Cluster Headache21
hc4
Probable Hemicrania Continua4
Probable Hemicrania Continua23
Probable Hemicrania Continua17
orbital or supraorbital or temporal pain
lacrimation

cluster_25
hc8
Probable Cluster Headache19
Probable Cluster Headache35
Probable Hemicrania Continua27
Probable Cluster Headache23
Probable Cluster Headache16
Probable Cluster Headache27
Probable Cluster Headache29
Probable Cluster Headache33
Probable Cluster Headache26
Probable Hemicrania Continua8
Probable Cluster Headache36
Probable Cluster Headache8
Probable Cluster Headache24
Probable Cluster Headache12
Probable Cluster Headache10

Probable Cluster Headache20
Probable Cluster Headache18
Probable Cluster Headache28
Probable Cluster Headache25
Probable Cluster Headache31
Probable Hemicrania Continua20
Probable Cluster Headache22
Probable Cluster Headache37
Probable Hemicrania Continua22
Probable Cluster Headache13
Probable Cluster Headache15
Probable Cluster Headache30
severe
every other day to 8 per day
15 to 180 minutes
restless

cluster_19
Probable Cluster Headache14
Probable Hemicrania Continua11
Probable Hemicrania Continua2
Probable Cluster Headache2
Probable Hemicrania Continua12
Probable Cluster Headache15
hc2
Probable Hemicrania Continua17
unilateral
eyelid edema

cluster_18
Probable Cluster Headache1
Probable Hemicrania Continua15
hc1
Probable Cluster Headache11
Probable Cluster Headache13
Probable Hemicrania Continua10
Probable Hemicrania Continua1
2 to 30 minutes
conjunctival injection

cluster_26
Probable Cluster Headache9
Probable Cluster Headache28
Probable Cluster Headache34
hc9
Probable Hemicrania Continua25

Probable Hemicrania Continua8
Probable Hemicrania Continua21
Probable Hemicrania Continua9
Probable Hemicrania Continua28
rhinorrhea

cluster_24
Probable Hemicrania Continua19
Probable Cluster Headache7
hc7
Probable Hemicrania Continua7
Probable Cluster Headache32
Probable Cluster Headache26
Probable Hemicrania Continua24
Probable Hemicrania Continua26
ptosis

Tension Type Headaches:

cluster_1
Probable Frequent Episodic Tension Type Headache50
Probable Frequent Episodic Tension Type Headache28
chronic tension type headache38
Probable Infrequent Episodic Tension Type Headache19
Probable Frequent Episodic Tension Type Headache15
Probable Frequent Episodic Tension Type Headache52
Probable Frequent Episodic Tension Type Headache76
chronic tension type headache22
Probable Chronic Tension Type Headache114
Probable Chronic Tension Type Headache115
Probable Infrequent Episodic Tension Type Headache31
Probable Frequent Episodic Tension Type Headache68
Probable Chronic Tension Type Headache108
Probable Frequent Episodic Tension Type Headache12
chronic tension type headache1
chronic tension type headache32
Probable Chronic Tension Type Headache121
Probable Infrequent Episodic Tension Type Headache8
Probable Chronic Tension Type Headache51
frequent tension type headache4
Probable Frequent Episodic Tension Type Headache74
chronic tension type headache18
Probable Chronic Tension Type Headache99
chronic tension type headache35
Probable Frequent Episodic Tension Type Headache23

Probable Frequent Episodic Tension Type Headache29
Probable Frequent Episodic Tension Type Headache69
Probable Chronic Tension Type Headache44
ndph1
Probable Chronic Tension Type Headache94
chronic tension type headache40
Probable Frequent Episodic Tension Type Headache33
Probable Chronic Tension Type Headache77
Probable Chronic Tension Type Headache83
Probable Chronic Tension Type Headache72
Probable Frequent Episodic Tension Type Headache30
chronic tension type headache31
Probable Chronic Tension Type Headache67
Probable Frequent Episodic Tension Type Headache67
chronic tension type headache44
Probable Chronic Tension Type Headache93
Probable Chronic Tension Type Headache4
Probable Chronic Tension Type Headache124
Probable Frequent Episodic Tension Type Headache9
chronic tension type headache16
frequent tension type headache1
Probable Chronic Tension Type Headache68
Probable Chronic Tension Type Headache58
Probable Chronic Tension Type Headache8
chronic tension type headache8
Probable Chronic Tension Type Headache5
Probable Chronic Tension Type Headache61
frequent tension type headache11
Probable Infrequent Episodic Tension Type Headache60
Probable Frequent Episodic Tension Type Headache3
Probable Chronic Tension Type Headache127
Probable Infrequent Episodic Tension Type Headache18
Probable Infrequent Episodic Tension Type Headache61
frequent tension type headache21
Probable Infrequent Episodic Tension Type Headache7
Probable Frequent Episodic Tension Type Headache5
Probable Frequent Episodic Tension Type Headache13
frequent tension type headache15
Probable Frequent Episodic Tension Type Headache55
Probable Frequent Episodic Tension Type Headache48
Probable Frequent Episodic Tension Type Headache38
Probable Frequent Episodic Tension Type Headache58
Probable Frequent Episodic Tension Type Headache44
Probable Frequent Episodic Tension Type Headache27
Probable Frequent Episodic Tension Type Headache32
chronic tension type headache36
Probable Frequent Episodic Tension Type Headache43
Probable Frequent Episodic Tension Type Headache18
Probable Chronic Tension Type Headache11
frequent tension type headache19

Probable Chronic Tension Type Headache57
Probable Chronic Tension Type Headache103
chronic tension type headache11
Probable Infrequent Episodic Tension Type Headache9
Probable Chronic Tension Type Headache90
Probable Chronic Tension Type Headache28
chronic tension type headache43
Probable Chronic Tension Type Headache100
Probable Chronic Tension Type Headache113
Probable Chronic Tension Type Headache12
chronic tension type headache41
Probable Frequent Episodic Tension Type Headache4
Probable Chronic Tension Type Headache13
Probable Chronic Tension Type Headache59
Probable Infrequent Episodic Tension Type Headache4
Probable Frequent Episodic Tension Type Headache36
chronic tension type headache4
Probable Frequent Episodic Tension Type Headache20
Probable Chronic Tension Type Headache38
chronic tension type headache6
Probable Chronic Tension Type Headache84
chronic tension type headache42
Probable Chronic Tension Type Headache122
Probable Frequent Episodic Tension Type Headache49
Probable Chronic Tension Type Headache101
frequent tension type headache18
Probable Chronic Tension Type Headache97
Probable Chronic Tension Type Headache27
Probable Chronic Tension Type Headache34
Probable Chronic Tension Type Headache86
Probable Chronic Tension Type Headache37
chronic tension type headache37
Probable Chronic Tension Type Headache116
Probable Frequent Episodic Tension Type Headache16
frequent tension type headache9
Probable Chronic Tension Type Headache46
Probable Chronic Tension Type Headache109
Probable Chronic Tension Type Headache52
Probable Frequent Episodic Tension Type Headache46
Probable Chronic Tension Type Headache54
Probable Frequent Episodic Tension Type Headache63
Probable Chronic Tension Type Headache47
Probable Chronic Tension Type Headache55
Probable Chronic Tension Type Headache32
chronic tension type headache23
Probable Frequent Episodic Tension Type Headache66
Probable Chronic Tension Type Headache53
Probable Chronic Tension Type Headache85
Probable Frequent Episodic Tension Type Headache14
Probable Frequent Episodic Tension Type Headache51

Probable Frequent Episodic Tension Type Headache72
Probable Chronic Tension Type Headache43
Probable Chronic Tension Type Headache130
Probable Chronic Tension Type Headache131
Probable Frequent Episodic Tension Type Headache42
Probable Chronic Tension Type Headache132
Probable Chronic Tension Type Headache60
Probable Chronic Tension Type Headache88
Probable Infrequent Episodic Tension Type Headache15
chronic tension type headache13
Probable Infrequent Episodic Tension Type Headache28
Probable Chronic Tension Type Headache120
frequent tension type headache2
Probable Frequent Episodic Tension Type Headache54
Probable Frequent Episodic Tension Type Headache70
Probable Frequent Episodic Tension Type Headache53
Probable Chronic Tension Type Headache10
Probable Chronic Tension Type Headache17
frequent tension type headache16
Probable Frequent Episodic Tension Type Headache26
Probable Chronic Tension Type Headache29
Probable Frequent Episodic Tension Type Headache64
Probable Chronic Tension Type Headache81
Probable Frequent Episodic Tension Type Headache40
chronic tension type headache24
Probable Chronic Tension Type Headache19
Probable Chronic Tension Type Headache106
Probable Chronic Tension Type Headache118
Probable Chronic Tension Type Headache6
frequent tension type headache14
Probable Frequent Episodic Tension Type Headache61
Probable Frequent Episodic Tension Type Headache56
Probable Chronic Tension Type Headache69
Probable Chronic Tension Type Headache48
Probable Chronic Tension Type Headache73
Probable Frequent Episodic Tension Type Headache8
chronic tension type headache33
Probable Chronic Tension Type Headache89
Probable Chronic Tension Type Headache92
Probable Frequent Episodic Tension Type Headache24
Probable Frequent Episodic Tension Type Headache75
Probable Chronic Tension Type Headache49
Probable Chronic Tension Type Headache63
Probable Infrequent Episodic Tension Type Headache16
Probable Chronic Tension Type Headache102
Probable Chronic Tension Type Headache42
Probable Chronic Tension Type Headache96
Probable Chronic Tension Type Headache107
Probable Infrequent Episodic Tension Type Headache29
Probable Chronic Tension Type Headache50

frequent tension type headache10
Probable Chronic Tension Type Headache105
Probable Frequent Episodic Tension Type Headache25
Probable Frequent Episodic Tension Type Headache22
Probable Chronic Tension Type Headache22
Probable Frequent Episodic Tension Type Headache21
Probable Frequent Episodic Tension Type Headache71
Probable Chronic Tension Type Headache14
Probable Infrequent Episodic Tension Type Headache20
frequent tension type headache12
Probable Infrequent Episodic Tension Type Headache27
Probable Frequent Episodic Tension Type Headache34
Probable Chronic Tension Type Headache39
Probable Frequent Episodic Tension Type Headache39
chronic tension type headache3
chronic tension type headache39
frequent tension type headache6
chronic tension type headache14
chronic tension type headache15
Probable Chronic Tension Type Headache64
frequent tension type headache5
Probable Chronic Tension Type Headache25
Probable Chronic Tension Type Headache21
Probable Chronic Tension Type Headache125
Probable Infrequent Episodic Tension Type Headache17
Probable Chronic Tension Type Headache98
chronic tension type headache21
Probable Chronic Tension Type Headache74
Probable Chronic Tension Type Headache129
Probable Frequent Episodic Tension Type Headache31
chronic tension type headache17
Probable Frequent Episodic Tension Type Headache19
Probable Chronic Tension Type Headache82
frequent tension type headache8
Probable Chronic Tension Type Headache104
Probable Chronic Tension Type Headache35
Probable Infrequent Episodic Tension Type Headache26
Probable Infrequent Episodic Tension Type Headache39
Probable Chronic Tension Type Headache30
Probable Frequent Episodic Tension Type Headache35
Probable Chronic Tension Type Headache70
Probable Chronic Tension Type Headache45
Probable Chronic Tension Type Headache24
Probable Chronic Tension Type Headache62
Probable Chronic Tension Type Headache33
chronic tension type headache7
Probable Chronic Tension Type Headache78
Probable Frequent Episodic Tension Type Headache11
frequent tension type headache3
Probable Chronic Tension Type Headache66

chronic tension type headache26
Probable Frequent Episodic Tension Type Headache2
Probable Chronic Tension Type Headache40
Probable Chronic Tension Type Headache128
Probable Frequent Episodic Tension Type Headache77
Probable Frequent Episodic Tension Type Headache10
Probable Chronic Tension Type Headache126
Probable Frequent Episodic Tension Type Headache60
chronic tension type headache29
Probable Chronic Tension Type Headache111
chronic tension type headache2
frequent tension type headache20
Probable Chronic Tension Type Headache80
Probable Chronic Tension Type Headache112
Probable Chronic Tension Type Headache117
Probable Infrequent Episodic Tension Type Headache5
Probable Frequent Episodic Tension Type Headache6
Probable Chronic Tension Type Headache56
chronic tension type headache28
chronic tension type headache27
Probable Infrequent Episodic Tension Type Headache30
Probable Chronic Tension Type Headache36
frequent tension type headache22
Probable Chronic Tension Type Headache110
Probable Frequent Episodic Tension Type Headache57
Probable Chronic Tension Type Headache95
Probable Frequent Episodic Tension Type Headache17
chronic tension type headache19
Probable Chronic Tension Type Headache91
Probable Frequent Episodic Tension Type Headache1
Probable Chronic Tension Type Headache71
Probable Frequent Episodic Tension Type Headache41
chronic tension type headache34
Probable Chronic Tension Type Headache31
chronic tension type headache30
Probable Chronic Tension Type Headache9
chronic tension type headache9
Probable Chronic Tension Type Headache79
frequent tension type headache13
chronic tension type headache25
chronic tension type headache12
Probable Frequent Episodic Tension Type Headache65
Probable Frequent Episodic Tension Type Headache45
Probable Frequent Episodic Tension Type Headache73
Probable Chronic Tension Type Headache41
Probable Frequent Episodic Tension Type Headache47
Probable Chronic Tension Type Headache119
Probable Frequent Episodic Tension Type Headache37
frequent tension type headache17
Probable Infrequent Episodic Tension Type Headache6

chronic tension type headache20
Probable Chronic Tension Type Headache123
Probable Frequent Episodic Tension Type Headache7
Probable Chronic Tension Type Headache26
Probable Chronic Tension Type Headache7
Probable Chronic Tension Type Headache65
Probable Chronic Tension Type Headache75
Probable Infrequent Episodic Tension Type Headache40
Probable Frequent Episodic Tension Type Headache62
chronic tension type headache5
Probable Frequent Episodic Tension Type Headache59
chronic tension type headache10
Probable Chronic Tension Type Headache87
Probable Chronic Tension Type Headache76
frequent tension type headache7
not aggrevated by activity
constant
mild to moderate pain
no photophobia
clearly remembered onset
no phonophobia
no nausea/vomiting
30 min to 7 days in duration
1 to 14 days per month
more than 3 months
nonpulsating
unremitting within 24 hours
hours to days
bilateral location

cluster_39
infrequent tension type headache1

cluster_40
infrequent tension type headache2

cluster_41
infrequent tension type headache3

cluster_43
infrequent tension type headache5

cluster_42
infrequent tension type headache4

cluster_44
infrequent tension type headache6

cluster_45
infrequent tension type headache7

cluster_46
infrequent tension type headache8

cluster_47
infrequent tension type headache9

cluster_48
infrequent tension type headache10

cluster_49
infrequent tension type headache11

cluster_50
infrequent tension type headache12

cluster_51
infrequent tension type headache13

cluster_52
infrequent tension type headache14

cluster_53
infrequent tension type headache15

cluster_54
infrequent tension type headache16

cluster_55
infrequent tension type headache17

cluster_56
infrequent tension type headache18

cluster_57
infrequent tension type headache19

cluster_58
infrequent tension type headache20

cluster_59
infrequent tension type headache21

cluster_60
infrequent tension type headache22

cluster_61
Probable Infrequent Episodic Tension Type Headache76
Probable Infrequent Episodic Tension Type Headache74
Probable Infrequent Episodic Tension Type Headache25
Probable Infrequent Episodic Tension Type Headache2
Probable Infrequent Episodic Tension Type Headache41
Probable Infrequent Episodic Tension Type Headache53
Probable Infrequent Episodic Tension Type Headache43
Probable Infrequent Episodic Tension Type Headache45
Probable Infrequent Episodic Tension Type Headache36
Probable Infrequent Episodic Tension Type Headache11
Probable Infrequent Episodic Tension Type Headache58
Probable Infrequent Episodic Tension Type Headache47
Probable Infrequent Episodic Tension Type Headache23
Probable Infrequent Episodic Tension Type Headache66
Probable Infrequent Episodic Tension Type Headache64
Probable Infrequent Episodic Tension Type Headache24
Probable Infrequent Episodic Tension Type Headache44
Probable Infrequent Episodic Tension Type Headache1
Probable Infrequent Episodic Tension Type Headache75
Probable Infrequent Episodic Tension Type Headache49
Probable Infrequent Episodic Tension Type Headache33
Probable Infrequent Episodic Tension Type Headache37
Probable Infrequent Episodic Tension Type Headache46
Probable Infrequent Episodic Tension Type Headache42
Probable Infrequent Episodic Tension Type Headache54
Probable Infrequent Episodic Tension Type Headache56
Probable Infrequent Episodic Tension Type Headache21
Probable Infrequent Episodic Tension Type Headache52
Probable Infrequent Episodic Tension Type Headache69
Probable Infrequent Episodic Tension Type Headache38
Probable Infrequent Episodic Tension Type Headache65
Probable Infrequent Episodic Tension Type Headache73
Probable Infrequent Episodic Tension Type Headache14
Probable Infrequent Episodic Tension Type Headache62
Probable Infrequent Episodic Tension Type Headache50
Probable Infrequent Episodic Tension Type Headache10
Probable Infrequent Episodic Tension Type Headache59
Probable Infrequent Episodic Tension Type Headache67
Probable Infrequent Episodic Tension Type Headache35
Probable Infrequent Episodic Tension Type Headache13
Probable Infrequent Episodic Tension Type Headache77
Probable Infrequent Episodic Tension Type Headache57
Probable Infrequent Episodic Tension Type Headache3
Probable Infrequent Episodic Tension Type Headache12

Probable Infrequent Episodic Tension Type Headache32
Probable Infrequent Episodic Tension Type Headache34
Probable Infrequent Episodic Tension Type Headache68
Probable Infrequent Episodic Tension Type Headache48
Probable Infrequent Episodic Tension Type Headache72
Probable Infrequent Episodic Tension Type Headache51
Probable Infrequent Episodic Tension Type Headache63
Probable Infrequent Episodic Tension Type Headache55
Probable Infrequent Episodic Tension Type Headache22
Probable Infrequent Episodic Tension Type Headache71
Probable Infrequent Episodic Tension Type Headache70
less than 12 days per year
more than 10 episodes

cluster_34
Probable Chronic Tension Type Headache16
Probable Chronic Tension Type Headache3
stabbing7
Probable Chronic Tension Type Headache2
hypnic7
Probable Chronic Tension Type Headache15
Probable Chronic Tension Type Headache18
Probable Chronic Tension Type Headache1
no ptosis

Migraine with and without aura

cluster_0
cm48
cm253
probable migraine with aura51
probable migraine with aura133
probable migraine with aura91
cm21
probable migraine without aura14
cm22
mwa69
probable migraine without aura70
mwa15
mwa88
migraine w/o aura14
probable migraine with aura76
probable migraine with aura113
mwa68
cm93

mwa95
mwa74
cm244
cm34
cm39
probable migraine with aura122
probable migraine without aura55
cm128
probable migraine with aura104
probable migraine with aura119
cm194
probable migraine with aura42
cm7
cm98
probable migraine with aura115
probable migraine with aura140
probable migraine with aura121
cm157
probable migraine without aura45
cm210
probable migraine with aura88
probable migraine without aura49
mwa19
cm67
cm41
cm6
cm199
probable migraine with aura89
cm221
mwa46
cm204
probable migraine with aura81
cm226
probable migraine without aura61
probable migraine with aura62
probable migraine with aura146
migraine w/o aura15
cm135
cm195
cm56
probable migraine without aura75
cm238
cm216
cm33
probable migraine with aura64
cm141
mwa12
probable migraine with aura142
mwa44
probable migraine with aura66

probable migraine with aura56
mwa116
cm36
mwa66
mwa26
migraine w/o aura12
mwa87
mwa99
cm198
cm2
mwa64
cm10
cm143
probable migraine with aura79
probable migraine with aura63
mwa82
mwa9
mwa55
cm94
migraine w/o aura1
cm40
probable migraine without aura41
probable migraine with aura53
cm20
migraine w/o aura21
cm78
mwa83
mwa36
probable migraine without aura9
cm179
probable migraine with aura61
probable migraine with aura128
cm3
cm190
cm110
cm32
probable migraine without aura38
cm239
cm72
cm58
probable migraine without aura35
mwa107
mwa51
cm107
cm250
mwa4
cm75
cm192
cm130
mwa18

migraine w/o aura8
probable migraine with aura57
cm163
probable migraine without aura72
cm230
cm241
probable migraine with aura108
cm178
mwa16
cm170
cm17
cm182
mwa17
cm234
probable migraine without aura1
migraine w/o aura19
cm196
probable migraine with aura68
cm200
cm245
cm203
cm145
cm134
cm69
cm219
cm108
probable migraine without aura23
mwa71
mwa59
mwa37
cm18
cm27
cm222
cm220
cm236
probable migraine without aura42
probable migraine with aura129
probable migraine with aura39
cm160
cm212
cm53
probable migraine without aura47
probable migraine with aura65
probable migraine with aura90
cm8
mwa101
cm81
cm174
probable migraine with aura120
mwa110

cm276
probable migraine with aura31
cm136
mwa11
cm83
cm138
cm91
cm258
probable migraine without aura69
mwa106
cm102
probable migraine without aura56
cm139
probable migraine without aura62
mwa75
cm270
mwa92
probable migraine without aura24
cm268
cm263
cm149
cm262
cm209
probable migraine without aura54
cm13
cm116
mwa104
probable migraine with aura55
probable migraine with aura70
mwa119
cm224
mwa61
probable migraine without aura51
probable migraine with aura77
migraine w/o aura17
probable migraine with aura48
cm28
probable migraine with aura47
cm158
cm187
cm146
migraine w/o aura13
cm105
mwa14
probable migraine with aura25
probable migraine without aura66
mwa63
mwa25
cm106
cm115

cm171
cm151
cm61
cm11
mwa42
cm85
cm62
probable migraine with aura78
cm4
cm16
probable migraine with aura35
probable migraine with aura94
 migraine w/o aura6
probable migraine without aura40
cm243
probable migraine without aura76
cm19
 migraine w/o aura9
probable migraine with aura43
cm101
cm186
cm189
cm120
cm95
cm50
probable migraine without aura4
probable migraine with aura58
probable migraine without aura6
cm87
mwa38
cm197
cm68
probable migraine without aura43
probable migraine without aura53
cm235
probable migraine with aura67
probable migraine with aura83
cm191
probable migraine with aura98
probable migraine with aura102
probable migraine without aura52
probable migraine without aura63
probable migraine with aura59
cm44
cm90
cm155
cm133
probable migraine with aura130
cm30
cm169

cm242
probable migraine without aura29
mwa70
cm46
cm150
mwa81
cm42
migraine w/o aura22
probable migraine with aura107
mwa8
mwa2
probable migraine with aura23
cm255
cm111
cm26
cm215
cm112
mwa103
mwa102
probable migraine without aura36
probable migraine with aura32
mwa1
probable migraine without aura22
probable migraine with aura126
mwa35
cm156
cm193
cm240
mwa91
cm225
probable migraine with aura60
probable migraine with aura114
probable migraine with aura71
cm88
probable migraine without aura59
cm152
probable migraine without aura11
cm228
cm188
cm259
mwa90
cm57
cm15
probable migraine with aura116
mwa57
cm172
cm148
probable migraine without aura30
probable migraine with aura82
cm45

cm113
cm122
 migraine w/o aura7
probable migraine with aura135
cm218
mwa65
cm229
mwa58
cm260
cm1
mwa117
probable migraine without aura21
probable migraine with aura118
mwa97
probable migraine with aura26
probable migraine without aura58
cm140
mwa108
mwa86
mwa72
cm104
cm272
cm162
mwa100
mwa105
cm247
probable migraine without aura39
cm14
probable migraine with aura139
mwa34
cm264
probable migraine with aura75
probable migraine without aura3
probable migraine without aura65
probable migraine with aura73
mwa62
mwa109
probable migraine with aura132
probable migraine with aura125
mwa30
cm279
mwa118
mwa73
cm267
probable migraine without aura31
probable migraine without aura73
probable migraine with aura36
cm284
probable migraine with aura105
cm124

cm233
probable migraine with aura24
probable migraine without aura74
probable migraine with aura103
mwa39
cm277
probable migraine without aura8
mwa6
mwa3
cm246
cm176
cm132
mwa93
cm201
probable migraine with aura143
mwa48
mwa114
mwa120
probable migraine with aura38
mwa33
cm47
probable migraine without aura44
probable migraine with aura30
mwa79
probable migraine without aura7
probable migraine without aura16
cm76
probable migraine with aura72
probable migraine with aura131
cm49
cm77
cm100
cm123
mwa60
cm217
cm70
migraine w/o aura18
mwa13
mwa111
probable migraine without aura12
cm82
migraine w/o aura16
probable migraine without aura2
probable migraine with aura85
cm79
cm208
probable migraine with aura111
mwa53
cm166
cm65

migraine w/o aura20
cm121
mwa84
mwa80
cm12
migraine w/o aura2
cm43
cm55
probable migraine with aura123
cm248
probable migraine with aura80
probable migraine with aura50
probable migraine without aura17
cm71
probable migraine with aura97
probable migraine without aura34
probable migraine without aura15
probable migraine with aura84
cm38
cm159
cm51
mwa45
cm142
mwa96
cm252
probable migraine with aura137
cm180
cm131
migraine w/o aura11
cm269
probable migraine without aura5
mwa43
mwa112
cm59
probable migraine with aura69
cm9
mwa47
mwa23
probable migraine without aura27
cm126
mwa49
cm92
cm86
cm96
cm144
mwa29
mwa56
cm206
probable migraine with aura44
cm117

cm66
probable migraine with aura96
probable migraine with aura52
probable migraine with aura95
probable migraine with aura46
cm114
mwa24
cm60
cm119
cm181
cm99
cm275
mwa31
probable migraine without aura20
cm167
probable migraine with aura109
cm29
cm109
probable migraine with aura101
probable migraine with aura40
cm168
probable migraine with aura124
probable migraine with aura145
cm37
cm183
probable migraine with aura34
probable migraine with aura49
probable migraine without aura60
probable migraine with aura54
cm125
probable migraine without aura37
cm231
probable migraine without aura32
mwa20
 migraine w/o aura4
cm254
probable migraine with aura112
cm177
cm282
mwa94
probable migraine with aura86
probable migraine with aura117
cm280
cm52
cm127
cm271
cm129
mwa76
probable migraine without aura46
probable migraine with aura45

probable migraine with aura110
cm266
mwa28
cm207
probable migraine without aura33
probable migraine with aura99
mwa32
cm5
cm154
cm214
mwa7
probable migraine without aura64
cm281
cm63
cm31
probable migraine without aura71
cm185
mwa67
probable migraine with aura144
cm103
mwa41
probable migraine without aura67
probable migraine without aura68
cm84
probable migraine with aura93
cm89
probable migraine without aura57
cm257
mwa21
cm73
mwa89
probable migraine with aura87
mwa22
cm23
migraine w/o aura5
cm184
probable migraine with aura134
probable migraine with aura106
mwa113
probable migraine with aura100
mwa27
cm213
cm237
migraine w/o aura3
cm175
probable migraine without aura18
cm256
cm273
cm165
cm232

probable migraine without aura10
mwa10
cm223
probable migraine without aura25
cm147
probable migraine with aura37
cm283
mwa52
cm261
probable migraine without aura48
probable migraine with aura127
cm97
cm249
probable migraine with aura138
mwa98
mwa5
cm164
cm137
mwa78
probable migraine with aura136
cm54
cm205
probable migraine without aura28
mwa115
mwa85
cm80
cm118
cm173
cm25
migraine w/o aura10
mwa54
cm227
probable migraine without aura77
probable migraine with aura74
cm24
cm35
probable migraine with aura141
cm161
mwa50
cm74
cm211
cm202
probable migraine with aura41
probable migraine without aura50
cm278
probable migraine with aura92
probable migraine without aura19
cm265
cm274
mwa40

cm64
mwa77
cm251
cm153
probable migraine without aura26
relieve by triptan or ergot
moderate to severe
at least one aura symptom is unilateral
visual aura
greater than 15 days per month
fully reversible
aggravated by physical activity
photophobia
motor aura
phonophobia
the aura is accompanied, or followed within 60 minutes, by headache
two or more aura symptoms occur in succession
pulsating
sensory aura
at least one aura symptom is positive
greater than 2 episodes
speech and/or language aura
nausea/vomiting
4 to 72 hours
each individual aura symptom lasts 5–60 minutes
greater than 5 episodes
at least one aura symptom spreads gradually over 5 minutes
brainstem aura
greater than 8 days per month
retinal aura

Primary sex headaches

cluster_27
probable migraine with aura22
probable migraine with aura18
probable migraine with aura3
probable migraine with aura2
probable migraine with aura17
probable migraine with aura27
probable migraine with aura33
probable migraine with aura13
probable migraine with aura20
probable migraine with aura21
probable migraine with aura28
sex1
probable migraine with aura8

probable migraine with aura9
probable migraine with aura11
probable migraine with aura14
probable migraine with aura4
probable migraine with aura12
probable migraine with aura6
probable migraine with aura10
sex4
probable migraine with aura29
probable migraine with aura7
sex3
probable migraine with aura5
probable migraine with aura15
probable migraine with aura16
probable migraine with aura19
sex2
probable migraine with aura1
abrupt explosive intensity just before or with orgasm
1 minute to 72 hours with severe
up to 72 hours with mild
increasing in intensity with increasing sexual excitement
brought on by sex

Primary stabbing and hypnic headache:

cluster_28
stabbing1
hypnic1
15 minutes up to four hours after waking
more than 10 days per month
developing only during sleep and causing wakening
no conjunctival injection

cluster_29
stabbing2
hypnic2
no eyelid edema

cluster_35
hypnic8
stabbing8
no rhinorrhea

cluster_31
stabbing4

Probable Chronic Tension Type Headache23
hypnic4
no lacrimation

cluster_30
hypnic3
stabbing3
Probable Chronic Tension Type Headache20
no forehead and facial sweating

cluster_32
stabbing5
hypnic5
single or series of stabs
no miosis
irregular frequency
up to few seconds

cluster_33
hypnic6
stabbing6
no nasal congestion

Cough and Valsalva headache:

cluster_37
cough1
sudden
between 1 second to 2 hours
provoke by cough

cluster_38
cough2
provoke by valsalva

____

Exercise headaches:

cluster_2
exercise1

brought on by exercise
less than 48 hours

Traction and compression headaches:

cluster_5
compression1
brought on within 1 hour of compression
maximal at site of compression
resolve within 1 hour after removal of compression

cluster_6
traction1
brought on within 1 hour of traction
maximal at site of traction
resolve within 1 hour after removal of traction

Cold induced headaches:

cluster_4
coldHA1
brought on by cold stimuli
resolve within 30 min after removal of cold

Nummular headache:

cluster_36
nummular1
fixed in size and shape
sharply contoured
1–6 cm in diameter
round or elliptical

Phenotypes only:

cluster_62
more than 1 episode per day

cluster_63
no orbital or supraorbital or temporal pain

cluster_64
no restless
